# Investigating Metal and Fluorophore Controlled Intracellular Localization in Noble Metal Thiosemicarbazone Complexes

**DOI:** 10.1002/chem.202502613

**Published:** 2025-11-05

**Authors:** Nandan Sheernaly, Axel Steinbrueck, Nicolas Krahn, Christoph Rumancev, Frank Peeters, Lejla Jusufagic, Jasmine Ochs, Jan Garrevoet, Gerald Falkenberg, Axel Rosenhahn, Nils Metzler‐Nolte

**Affiliations:** ^1^ Faculty of Chemistry and Biochemistry Inorganic Chemistry I – Bioinorganic Chemistry Ruhr University Bochum Universitaetsstrasse 150 Bochum 44801 Germany; ^2^ Faculty of Chemistry and Biochemistry Analytical Chemistry – Biointerfaces Ruhr University Bochum Universitaetsstrasse 150 Bochum 44801 Germany; ^3^ Faculty of Biology and Biotechnology Applied Microbiology Ruhr University Bochum Universitaetsstrasse 150 Bochum 44801 Germany; ^4^ Deutsches Elektronen‐Synchrotron DESY Notkestrasse 85 Hamburg 22607 Germany

**Keywords:** confocal microscopy, dp44mt, fluorophore conjugates, imaging, inorganic chemical biology, localization patterns, metal complexes, thiosemicarbazones, x‐ray fluorescence imaging

## Abstract

Transition metal complexes have been widely utilized as cellular imaging tools. To impart organelle specificity, ligand architecture is usually modified to modulate properties like overall charge and lipophilicity. In many such designs, the metal identity and its intrinsic properties are often ignored. To address this gap, in this study, we explored the effects of changing the metal center on the localization patterns of isostructural complexes. To this end, we employed the thiosemicarbazone Dp44mT to synthesize coumarin‐conjugated complexes of Au(III), Pt(II), and Pd(II). Although the metal centers in these compounds share a formal d^8^ configuration, they differ in properties such as ionic radius, charge density, and ligand exchange rates, which can affect their subcellular localization patterns. In addition, we synthesized a second set of analogous complexes using BODIPY as the conjugating fluorophore to assess the influence of using a different dye on the cellular distribution. Confocal imaging revealed that the complexes exhibited distinct intracellular distributions. For instance, while the coumarin‐conjugated Pt(II) complex localized specifically in lysosomes, the corresponding lipophilic Pd(II) complex lacked this specificity and instead followed a diffusely cytosolic distribution. Similarly, the more lipophilic BODIPY conjugated complexes were non‐specific in their cellular distribution as well. Overall, the findings of this study highlight the interplay of metal identity and lipophilicity in determining the localization patterns of Dp44mT‐based metal complexes, offering fresh insights into the design of new metal‐based imaging tools.

## Graphical abstract

Same ligand, same dye, different metal: Thiosemicarbazone conjugates of Pt and Pd show distinctly different localization patterns inside mammalian cells.

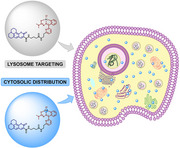

## 1 Introduction

Transition metal complexes have shown great promise as therapeutic and diagnostic agents.^[^ ^1^, ^2^, ^3^, ^4^, ^5^ ^]^ Despite the clinical success of metal complexes like cisplatin in treating cancer,^[^ ^6^ ^]^ undesired side effects remain a cause for concern.^[^ ^7^ ^]^ Over the years, metal complexes have been modified to selectively target organelles affected by their mode of action, thereby improving tumor specificity and minimizing systemic toxicity.^[^ ^8^, ^9^ ^]^ A particularly effective strategy has been to optimize ligand morphologies. For example, incorporating DNA‐intercalating ligands, such as acridine and anthracene, in the design of metal complexes promotes nuclear accumulation.^[^ ^10^, ^11^, ^12^ ^]^ Similarly, cationic hydrophobic moieties like triphenylphosphonium confer mitochondrial specificity to metal complexes.^[^ ^13^ ^]^ Hence, factors such as the overall charge of the metal complex and lipophilicity can be tuned to modulate subcellular distribution.^[^ ^8^ ^]^ While these ligand‐dependent factors are vital, the role of the metal center itself in influencing the subcellular distribution should not be overlooked. The identity of the metal and its intrinsic properties, such as ionic radius, charge density, and oxidation number, influence the metal‐ligand interactions, which in turn affect the metal complex's ability to interact with biomolecules, thereby directing its subcellular localization patterns.^[^ ^14^ ^]^ For instance, Zhang et al. demonstrated that complexing Zn to salen ligands caused the localization preference to change from mitochondria to lysosomes.^[^ ^15^ ^]^ Herein, we studied the effect of varying the metal center on the cellular localization patterns of complexes synthesized with di‐2‐pyridylketone‐4,4‐dimethyl‐3‐thiosemicarbazone (Dp44mT) as the coordinating ligand.

**Figure 1:**
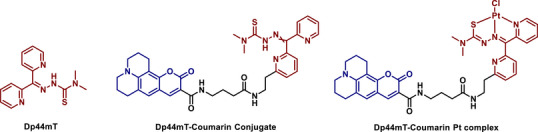
Structures of Dp44mT, its coumarin conjugate, and the corresponding Pt complex.

Dp44mT belongs to the di‐2‐pyridyl‐thiosemicarbazones (DpTs) class of chelators, known to target lysosomes specifically. DpTs exert anti‐cancer activity by employing their *N*,*N*,*S* donor motif to bind Cu(II) and Fe(II) in lysosomes, forming redox‐active complexes.^[^ ^16^ ^]^ These complexes generate reactive oxygen species (ROS), leading to lysosome membrane permeabilization (LMP) and eventually apoptosis.^[^ ^17^, ^18^ ^]^ Furthermore, the iron‐binding ability affects various cellular targets, such as the inhibition of ribonucleotide reductase,^[^ ^19^ ^]^ and upregulation of *N‐myc downstream regulated gene 1* (NDRG1).^[^ ^20^ ^]^ Among the many DpTs studied, Dp44mT emerged as the lead candidate in the first generation of compounds, demonstrating excellent activity both in vitro and in vivo.^[^ ^21^ ^]^ In our previous study, we reported a coumarin‐conjugated Dp44mT derivative as a theranostic agent, which confirmed that Dp44mT indeed localized in lysosomes. Furthermore, we showed that complexing Pt to the conjugate suppressed its cytotoxic activity in vitro while retaining the lysosomal targeting ability (Figure 1).^[^ ^22^ ^]^

Considering that the anti‐cancer activity of DpTs is reliant on Cu(II) and Fe(II) complexation and given their ability to form complexes with a variety of transition metals, we utilized our reported ligand system to explore the influence of varying the metal center on the cellular localization pattern.^[^ ^23^ ^]^ For this, we ensured that the complexes were isostructural, thereby allowing us to specifically isolate the effects imparted by the metal center. Accordingly, we synthesized square‐planar complexes of the Dp44mT‐coumarin conjugate with Au(III) and Pd(II) and compared their localization patterns to that of our reported Pt(II) complex. Despite the d^8^ configuration and identical geometries of these complexes, we envisioned that the differences in their intrinsic properties would influence their cellular behavior. Pd(II) with its smaller ionic radius and higher charge density as compared to Pt(II) demonstrates higher ligand exchange rates.^[^ ^24^ ^]^ On the other hand, the higher reduction potential of Au(III) has been known to influence complex stability in biological medium.^[^ ^25^ ^]^ Furthermore, we examined the influence of using a different dye on the localization patterns of these complexes by conjugating them with BODIPY. This increased the lipophilicity of the complexes, thereby altering the intracellular distribution, especially in the case of the Pt(II) complex. In the following sections, we describe the synthesis of the conjugates, evaluation of their cytotoxic activity, and exploration of their intracellular distributions using confocal microscopy.

## 2 Results and Discussion

### 2.1 Synthesis and Characterization

**Figure 2:**
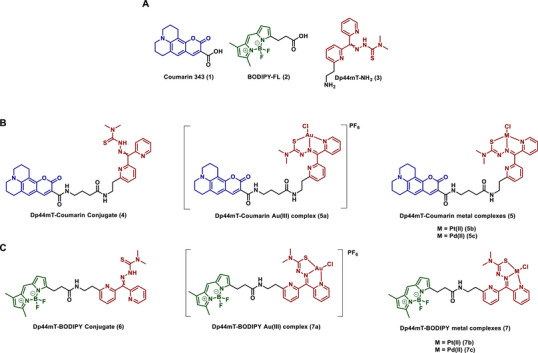
A): Conjugation fragments **1‒3**. B) and C): Dp44mT conjugates of Coumarin **4** and BODIPY **6**. Their respective Au(III), Pt(II), and Pd(II) complexes **5a–c** and **7a–c**.

The syntheses of the amine‐functionalized Dp44mT (**3**) and the coumarin conjugate (**4**) were performed as previously reported and were acquired as a 1:1 mixture of *E*/*Z* isomers (Figure 2A,B).^[^ ^22^, ^26^ ^]^ For the synthesis of the second fluorophore conjugate, we selected BODIPY‐FL (**2**) (λ_exc_/λ_em_: 502/512 nm). Unlike coumarin 343 (**1**), **2** possesses an intrinsic ethyl spacer, which eliminates the need to install an additional linker moiety. The conjugation was performed using standard peptide coupling conditions, affording the Dp44mT‐BODIPY conjugate (**6**) as a mixture of *E*/*Z* isomers in 65% yield (Figure 2C).

The ligands **4** and **6** were then complexed with Au(III), Pt(II), and Pd(II) using their respective metal salts to yield the desired metal complexes **5a–c** and **7a–c** (Figure 2B,C) (see Supporting Information for detailed synthetic schemes, procedures, and characterization data). Similar to the Pt(II) complex **5b**, all resulting complexes were isolated as diastereomerically pure compounds. Notably, mass spectrometric analysis of **5c, 7b**, and **7c** (Figures S26, S22, and S30) revealed that the predominant species resulted from chloride dissociation at the respective metal centers. However, in the case of **5b**, the predominant species retained chloride at the Pt(II) center.^[^ ^22^ ^]^

**Figure 3:**
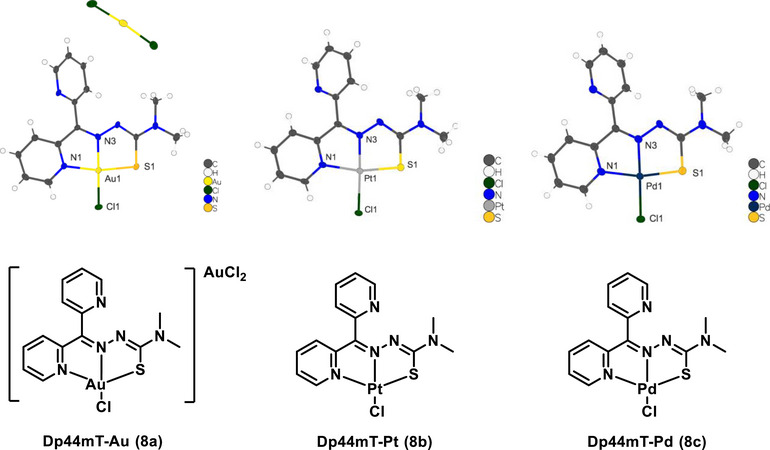
Structures of Pt(II), Pd(II), and Au(III) complexes of parent Dp44mT **8a**‒**c,** as determined by single crystal X‐ray diffraction. Ellipsoids are drawn at 50% probability.

To gain further insights into the geometries of **5** and **7**, we synthesized and grew single crystals of the three respective metal complexes of parent chelator Dp44mT (**8a**‒**c**) (Figure 3). X‐ray crystallographic analysis confirmed that Dp44mT coordinates with each metal ion in a 1:1 stoichiometry. The complexes crystallized in the monoclinic space group, adopting distorted square planar geometries, with N1‐M1‐N3 and N3‐M1‐S1 bond angles of ∼81° and ∼85°, respectively, consistent with analogous Au(III), ^[^ ^27^ ^]^ Pt(II), ^[^ ^28^, ^29^ ^]^ and Pd(II)^[^ ^28^, ^30^ ^]^ complexes. Furthermore, to assess deviations from ideal square‐planar geometry, the four‐coordinate geometry indices *τ*_4_ and *τ’*_4_, introduced by Houser et al.^[^ ^31^ ^]^ and Okuniewski et al.,^[^ ^32^ ^]^ respectively, were calculated: **8a** *τ*_4_ = 0.096, *τ’*_4 _= 0.062; **8b** *τ*_4 _= 0.113, *τ’*_4 _= 0.077; **8c** *τ’*_4 _= 0.108, *τ’*_4_ = 0.070. While a value of 0 corresponds to a perfect square plane, a value of 1 corresponds to an ideal tetrahedron. The *τ* parameters for all complexes in our case are close to 0, confirming that they adopt near square‐planar geometries.

### 2.2 Stability of the Complexes

**Figure 4:**
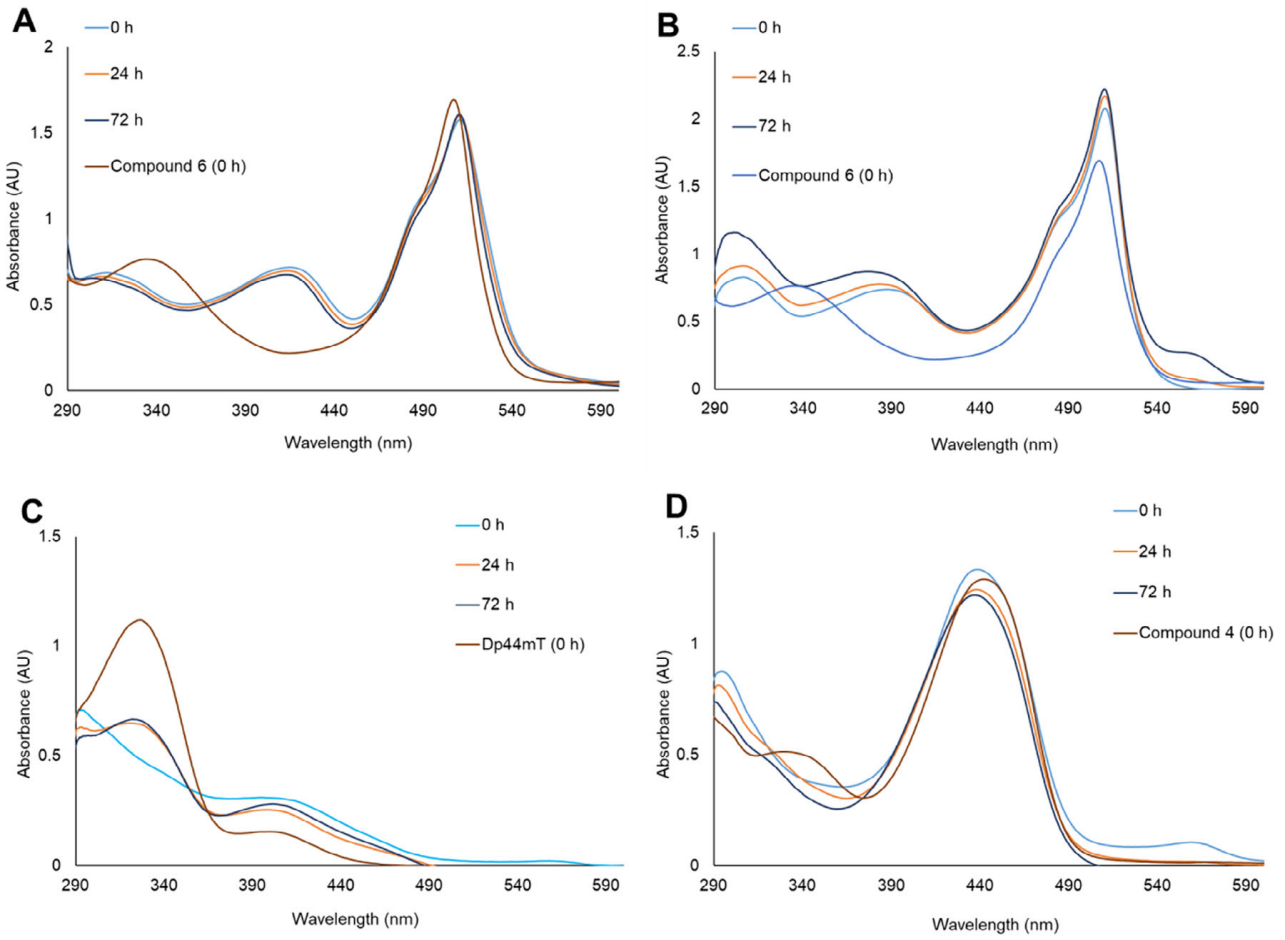
UV/Vis absorption spectra of 50 µM solutions of A) Pt(II) complex **7b,** B) Pd(II) complex **7c,** C) Au(III) complex **8a,** and D) Au(III) complex **5a** measured in cell culture medium. Absorption spectra of the corresponding ligands in cell culture medium have been included for reference. Spectra of the remaining compounds can be found in the Supporting Information.

Previously, we demonstrated via ^1^H NMR spectroscopy that the Dp44mT‐coumarin conjugate **4** and the Pt(II) complexes **5b** and **8b** were stable in DMSO‐d_6_ over 24 hours. Likewise, the stability of these complexes was shown via UV/Vis spectrophotometry in cell culture medium over 72 hours.^[^ ^22^ ^]^ The stability of the newly synthesized compounds was investigated similarly. While the Pt(II) and Pd(II) complexes were stable in DMSO‐d_6_ over 24 hours (Figures S41–S44), the Au(III) complexes showed signs of decomposition as new signals appeared in the ^1^H NMR spectrum after 24 hours. Consequently, spectra of the Au(III) complexes were recorded at shorter intervals, and the complexes were found to be stable for at least 4 hours (Figures S38–S40). Evaluation of stabilities in cell culture medium over 72 hours indicated that the Pt(II) complex **7b** (Figure 4A) remained stable up to this timepoint, a result consistent with the previously analyzed Pt(II) complexes **5b** and **8b**.^[^ ^22^ ^]^ However, the Pd(II) and Au(III) complexes exhibited the following discrepancies: in the case of the Pd(II) complexes, a blue shift in λ_max_ accompanied by a slight increase in absorbance was observed over time (Figure 4B shows absorbance profiles of **7c**, *cf*. Figure S45 for other Pd(II) complexes). However, the absorption profiles remained distinctly different from those of the corresponding free ligands, which was taken as evidence that the Pd(II) metal center remained chelated by the ligand. Interestingly, the blue shift was not observed when the absorption profiles were recorded in water alone, suggesting that unspecified components of the cell culture medium induced the phenomenon (*cf*. Figure S46).

In the case of the Au(III) complexes, both the BODIPY conjugated complex **7a** and the parent Dp44mT complex **8a** ostensibly decomposed in cell culture medium, as their absorption profiles in media lacked the characteristic charge transfer band at λ_max_ ∼440 nm observed in water. Moreover, the absorbance bands corresponding to the ligands emerged after 24 hours, suggesting metal dissociation (Figures 4C and S45, S46). In contrast, only mild degradation was observed in water after 72 hours, further indicating that the cell culture medium was detrimental to the stability of these complexes. The absorption profile of the Au(III) complex of the coumarin conjugate **5a**, on the other hand, did not appear to revert to that of its ligand **4** after 24 hours. However, a slight shift in the λ_max_ coupled with a decrease in absorbance was observed (Figure 4D). Given the instability of the other two Au(III) complexes, we inferred that **5a** had similarly decomposed in cell culture medium.

Additionally, the potency of DpT metal complexes can be influenced by their ability to undergo transmetallation with Cu(II) in lysosomal environments (pH 5.0).^[^ ^22^, ^30^ ^]^ Therefore, we assessed the stabilities of all complexes in acetate buffer (pH 5, 100 mM) in the presence of 5.0 equiv. of CuCl_2_ over 24 hours using UV/Vis spectroscopy (Figure S47). Gratifyingly, the absorption profiles remained unchanged for all complexes, indicating their stabilities toward Cu(II) in these conditions.

### 2.3 Lipophilicity

Having synthesized and assessed the stabilities of the complexes, we moved on to measure their lipophilicities at pH = 7.2 using the *n*‐octanol/PBS partition coefficient (Table 1).^[^ ^33^, ^34^ ^]^ While the complexes were all highly lipophilic, the BODIPY‐conjugated complexes were more lipophilic than their coumarin analogs. In both sets of conjugates, the log*D* values followed the trend Pd(II) > Pt(II) > Au(III), with the monocationic Au(III) complexes being the least lipophilic.

**Table 1:** Lipophilicity of metal complexes **5a‒c** and **7a‒c** displayed as log*D* values, determined from their *n*‐octanol/PBS distribution at pH 7.2. The values are presented as the mean of three independent measurements ± standard deviation (SD) values. See Supporting Information for further experimental details.

Lipophilicity		
Fluorophore	Compound	log*D*_7.2_
**Coumarin**	**5a** (Au(III))	0.99 ± 0.02
	**5b** (Pt(II))	1.47 ± 0.03
	**5c** (Pd(II))	1.67 ± 0.08
**BODIPY**	**7a** (Au(III))	1.53 ± 0.07
	**7b** (Pt(II))	1.80 ± 0.04
	**7c** (Pd(II))	2.46 ± 0.02

### 2.4 Antiproliferative Activity

We investigated the antiproliferative activities of compounds **4‒8** against HeLa cells using the standard 3‐(4,5‐dimethylthiazol‐2‐yl)‐2,5‐diphenyltetrazolium bromide (MTT) assay (Table 2). In our previous study, we confirmed that Dp44mT and related compounds (**4**, **5b**, **8b**) after 24 hours of incubation did not affect cell viability even at the highest tested concentration (15 µM). Consistent with these findings, the newly synthesized compounds also did not suppress cell viability after 24 hours of exposure.^[^ ^22^ ^]^

**Table 2:** In vitro activity of all compounds used in the study displayed as IC_50_ (µM) values determined by MTT assay. HeLa cells were incubated with the respective compounds for the indicated time period using Dp44mT as positive control and 0.5% DMSO as negative control. The values are presented as the mean of three independent measurements ± SD values. The individual dose‐response curves can be found in the Supporting Information (Figures S48‒S53).

IC_50_ [µM]			
Series	Compound	24 hours	72 hours
**Coumarin**	**4**	> 15	0.8 ± 0.1
	**5a**	> 15	3.1 ± 0.3
	**5b**	> 15	58.2 ± 4.3
	**5c**	> 15	31.6 ± 5.9
**BODIPY**	**6**	> 15	0.8 ± 0.1
	**7a**	> 15	0.6 ± 0.1
	**7b**	> 15	4.3 ± 0.3
	**7c**	> 15	> 15
**Parent**	**Dp44mT**	> 15	0.009 ± 0.0003
	**8a**	> 15	0.009 ± 0.0005
	**8b**	> 15	1.9 ± 0.3
	**8c**	> 15	2.6 ± 0.3

After 72 hours, the Dp44mT‐BODIPY conjugate **6** exhibited almost identical cytotoxicity as the coumarin conjugate **4** at 0.8 ± 0.1 µM, indicating that BODIPY conjugation was similarly detrimental to the potency of Dp44mT (IC_50_ = 9.0 ± 0.3 nM) as conjugation to coumarin. In contrast, the metal complexes displayed more diverse behaviors. Since the activity of Dp44mT depends on Cu(II) complexation, coordination with metals that do not undergo transmetallation with Cu(II) typically decreases its potency.^[^ ^30^ ^]^ Accordingly, Pt(II) and Pd(II) complexation diminished the cytotoxic effects with respect to their corresponding ligands. This decrease in activity exceeded a 50‐fold higher IC_50_ for the coumarin‐conjugated complexes (**5b**,**c**) and Dp44mT complexes (**8b**,**c**). Interestingly, for the BODIPY analogs, only the Pd(II) complex **7c** followed this trend, while the corresponding Pt(II) complex **7b** remained significantly more potent (IC_50_ = 4.3 ± 0.3 µM). Considering its inertness toward transmetallation with Cu(II), an alternative mode of action may be involved. On the other hand, the Au(III) complexes, which were unstable in cell culture medium, exhibited high cytotoxicity, which we attributed to the release of the cytotoxic free ligand. Thus, the in vitro activities of BODIPY‐Au(III) complex **7a** and parent Dp44mT‐Au(III) complex **8a** were comparable to their respective ligands. However, the coumarin‐conjugated complex **5a**, whose instability was less evident, demonstrated a fourfold lower activity, suggesting partial stability of the complex in cell culture medium. Overall, these findings suggest that the conjugated fluorophore and the resulting lipophilicity changes influence the in vitro activity of the metal complexes.

### 2.5 Uptake and Imaging Studies

Subsequently, we investigated the uptake of the conjugates **5** and **7** (at 10 µM) by incubating HeLa cells with the respective compounds for 24 hours and analyzing the final metal concentration inside the cells using inductively coupled plasma mass spectrometry (ICP‐MS). The presence of a significant amount of the respective metals was observed for each complex, suggesting that they had been successfully internalized into the cells (Table S3).

Before evaluating the cellular localization patterns of the compounds using confocal microscopy, we recorded their fluorescence excitation and emission spectra in water (10 µM) (Figures S54, S55). The coumarin‐conjugated compounds **4** and **5,** upon excitation at 450 nm, showed similar emission properties with λ_max_ at 492 nm. Likewise, the BODIPY‐conjugated compounds **7** and **8** showed emission maxima between 515 nm and 519 nm when excited at wavelengths between 502 nm and 510 nm. Notably, all metal complexes exhibited a seven to eightfold reduction in fluorescence intensity relative to their ligands, indicating that metal coordination induced fluorescence quenching. Nonetheless, the photophysical properties of the compounds were deemed sufficient to enable visualization of their subcellular localization patterns via confocal microscopy.

The subcellular behavior of the compounds was then assessed using a confocal microscope following incubation with HeLa cells (10 µM) for 6 hours and 24 hours, respectively (Figures S56, S57, and 5, 6). Moreover, we performed colocalization experiments with appropriate commercial organelle tracking dyes. As controls, we used the methyl and ethyl esters of **1** and **2, 1‐OMe** and **2‐OEt,** respectively. Consistent with prior observations with the coumarin conjugate **4**, the BODIPY conjugate **6** also localized in lysosomes, swelling them after 24 hours. Moreover, the Pearson Correlation Coefficients (PCC) between **6** and the commercial lysosome tracker (LysoTracker deep red) channel improved over time from 0.63 to 0.81. Additionally, the distribution was distinct from **2‐OEt**, corroborating that the Dp44mT scaffold directed the localization of the conjugates. Therefore, attaching BODIPY had no discernible effect on the lysosomal accumulation of Dp44mT.

**Figure 5:**
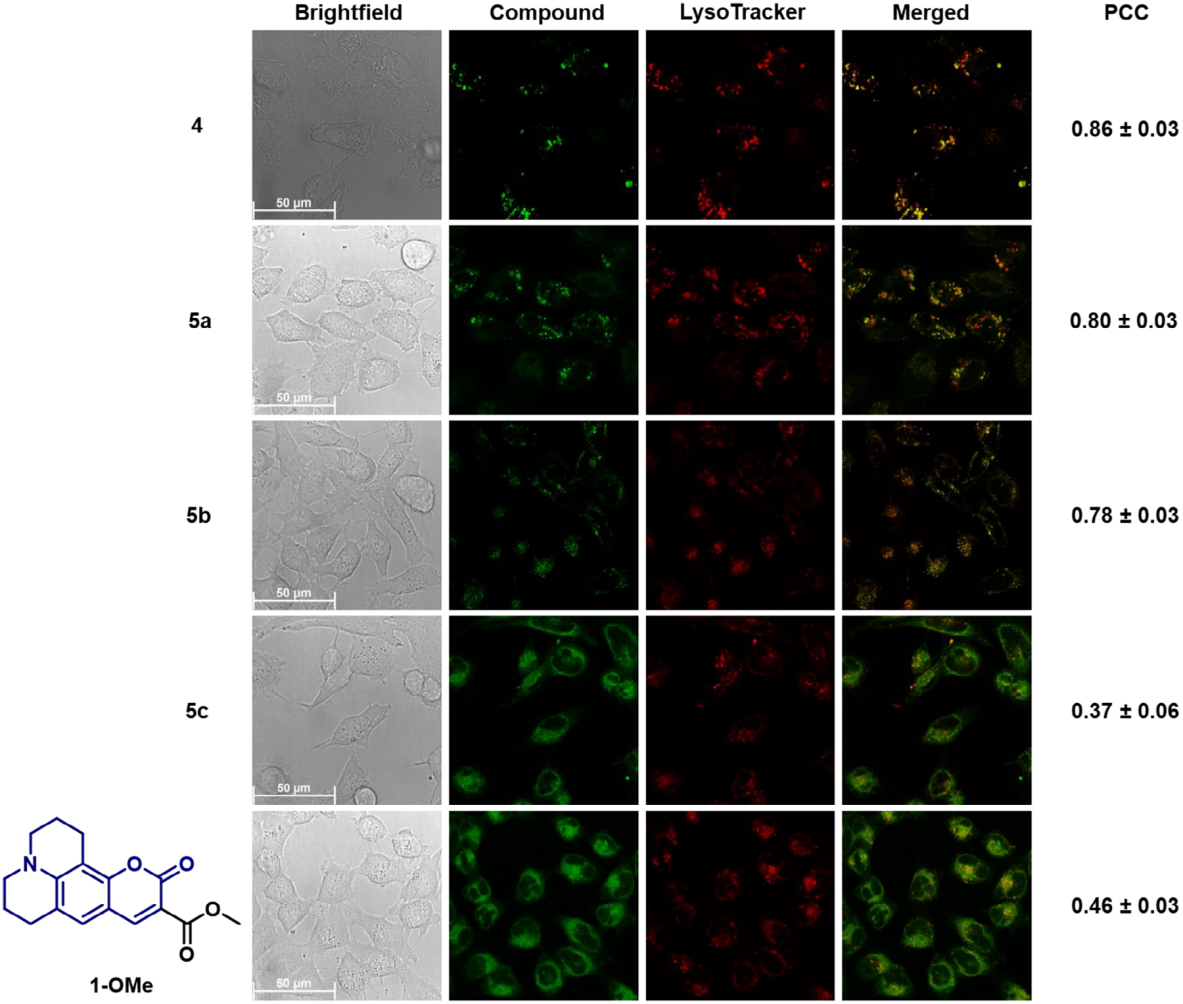
Live‐cell images acquired after incubating compounds **4**‒**5** and the control **1‐OMe** with HeLa cells for 24 hours, respectively, at 10 µM. The green channel shows the respective compound, and the red channel shows LysoTracker deep red. The merged channels and the corresponding Pearson Correlation Coefficients (PCC) ± standard deviation are shown on the right of each column. Further experimental details, images acquired after 6 hours incubation (Figure S56), and control images without tracker incubation can be found in the Supporting Information (Figures S58, S59).

**Figure 6:**
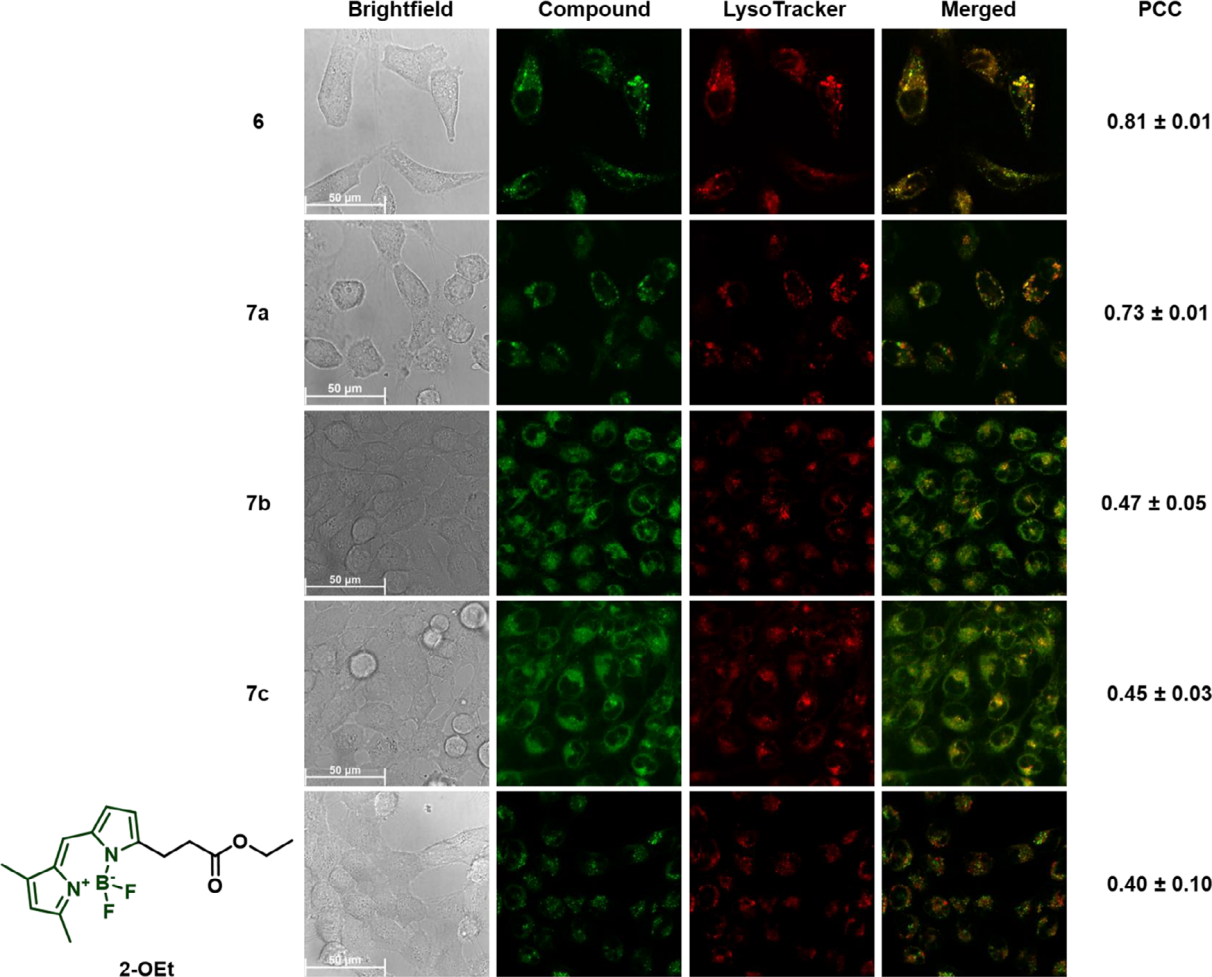
Live‐cell images acquired after incubating compounds **6**‒**7** and the control **2‐OEt** with HeLa cells for 24 hours, respectively, at 10 µM. The green channel shows the respective compound, and the red channel shows LysoTracker deep red. The merged channels and the corresponding Pearson Correlation Coefficients (PCC) ± standard deviation are shown on the right of each column. Further experimental details, images acquired after 6 hours incubation (Figure S57), and control images without tracker incubation can be found in the Supporting Information (Figures S58, S60).

A posteriori, we hypothesized that stable metal complexes of Dp44mT would localize in lysosomes without enlarging them after 24 hours.^[^ ^22^ ^]^ However, the Au(III) complexes **5a** and **7a**, due to their instability in cell culture medium, exhibited localization patterns mirroring those of their ligands at both time points. Notably, by 24 hours of exposure, swollen lysosomes were expectedly observed for both Au(III) complexes, corroborating that Au indeed dissociated from the ligand in cell culture medium. Interestingly, though, the coumarin‐conjugated complex **5a** demonstrated PCCs significantly superior to that of its ligand after 6 hours of incubation. Considering its instability in cell culture medium was not conclusively established and Au uptake was confirmed during uptake studies, the improved lysosomal localization of **5a** may be attributed to Au remaining associated with the ligand at the earlier 6‐hour exposure time point. This notion is further supported by the lower potency of **5a** compared to the metal‐free ligands and the Au(III) complex **7a**.

**Figure 7:**
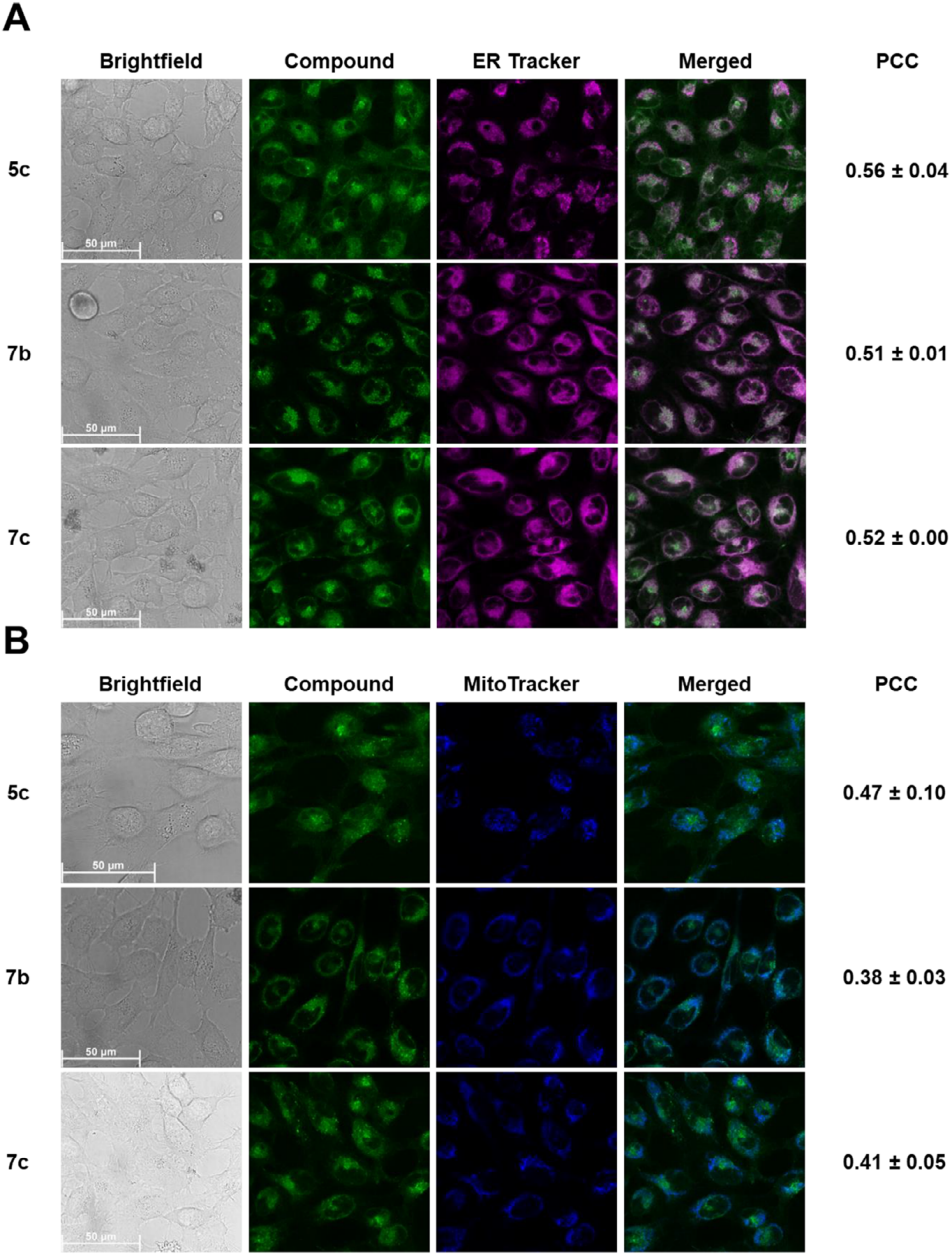
Live‐cell images acquired after incubating compounds **5c**, **7b**‒**c** with HeLa cells for 24 hours at 10 µM. Colocalization experiments were performed with A) ER Tracker (magenta channel) and B) MitoTracker (blue channel). The merged channels and the corresponding Pearson Correlation Coefficients (PCC) ± standard deviation have been shown.

On the other hand, despite their stability in cell culture medium, the Pt(II) and Pd(II) complexes showed different behaviors. As noted earlier, the coumarin‐conjugated Pt(II) complex **5b** preferentially localized within lysosomes at both time points without inducing lysosomal enlargement after 24 hours (Figure 5). In contrast, the coumarin‐conjugated Pd(II) complex **5c** and the two BODIPY‐conjugated complexes **7b** and **7c** lacked such a distinct preference for lysosomes, as their fluorescence appeared distributed throughout the cytoplasm (Figure 6). Although **7b** and **7c** displayed occasional punctate structures that partially colocalized with the LysoTracker at both time points, their fluorescence distribution was predominantly cytosolic (*cf*. Figure S60 for control images without tracker incubation). Consequently, to evaluate the cellular localization patterns of **5c, 7b**, and **7c** further, we performed colocalization studies with the commercial endoplasmic reticulum localizing dye (ER‐Tracker red) and mitochondria localizing dye (MitoTracker deep red) after 24 hours of incubation (Figure 7A,B). While PCCs with the ER‐Tracker were slightly higher than with the MitoTracker, all values were moderate, indicating no strong preference for either organelle. These results provide strong support that these complexes are spread out diffusely across the entire cell, including the nucleus, without specifically accumulating in any organelle.

**Figure 8:**
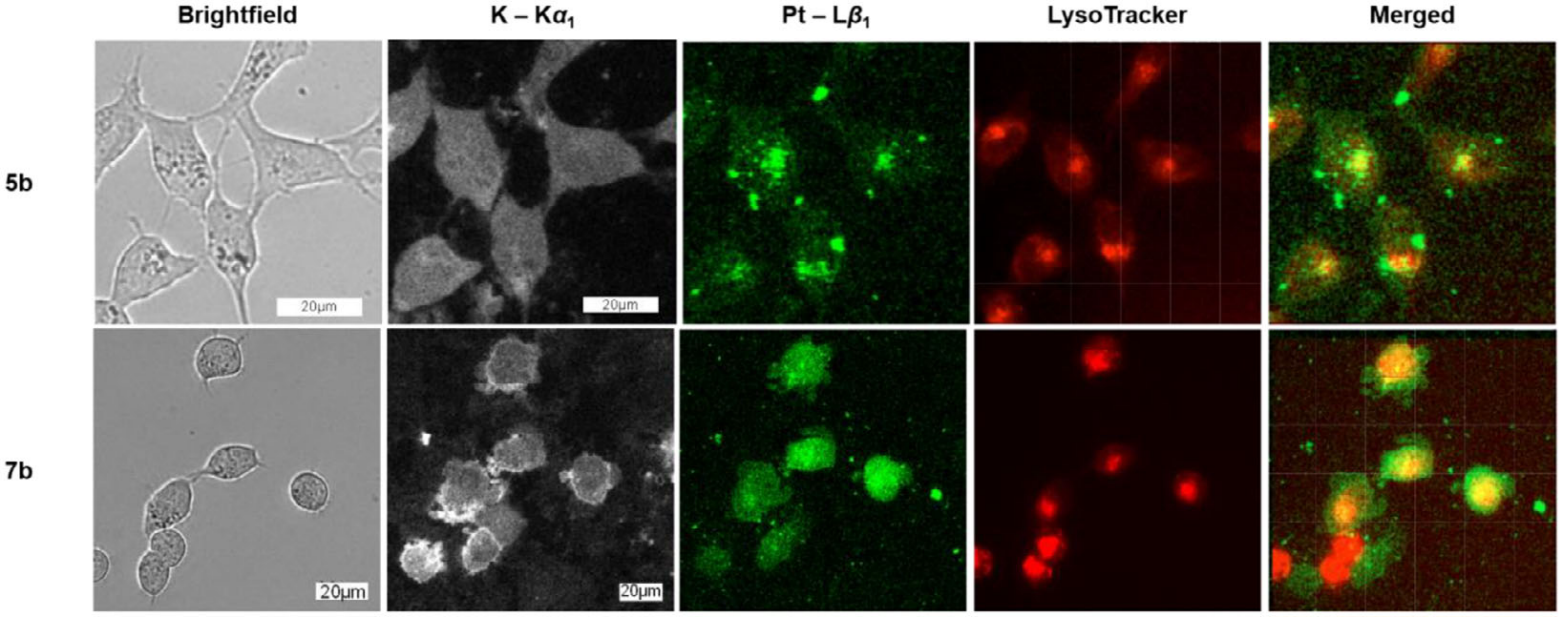
XRF imaging of HeLa cells incubated with complexes **5b** and **7b** at 30 µM for 24 hours. The images in brightfield and LysoTracker channels were acquired using a fluorescence microscope. Potassium (K – K*α*_1_) and Platinum (Pt – L*β*_1_) channels are spatially resolved XRF intensities showing the respective elemental distributions. The merged channel shows an overlap of the platinum (Pt – L*β*_1_) and LysoTracker channels. Further experimental details and untreated control images can be found in the Supporting Information (Figure S61).

To further validate the stark difference in the localization patterns between the coumarin‐conjugated Pt(II) complex **5b** and the remaining Pt(II) (**7b**) and Pd(II) complexes (**5c** and **7c**), we envisioned investigating their subcellular distributions via X‐ray fluorescence (XRF) imaging.^[^ ^35^ ^]^ While XRF has been used to determine subcellular distributions of Pt complexes,^[^ ^36^ ^]^ the technique has not been applied for the analysis of Pd complexes in cells, to the best of our knowledge. Nevertheless, we harnessed our experience and proceeded with the preparations for XRF samples for 2D imaging of HeLa cells (30 µM).^[^ ^37^ ^]^ As illustrated in Figure 8, the uptake of Pt complexes **5b** and **7b** was clearly detected, with the intracellular Pt amounts comparable to the amounts determined using ICP‐MS (*cf*. Figure S66 for the amounts of Pt taken up). Notably, the subcellular distribution of Pt for **5b** colocalized with the LysoTracker, whereas **7b** demonstrated a diffusely cytosolic distribution. These findings perfectly mirrored those from confocal microscopy and additionally confirmed that Pt remained bound to the respective ligands within the cells. However, we could not assess the uptake of the Pd(II) complexes due to the overlapping of the X‐ray emission energies of Pd (L*α*_1_) and Cl (K*β*_1_) (Figures S64, S65).

Therefore, the lysosomal preference of Dp44mT, which is retained by its coumarin‐conjugated Pt(II) complex **5b**, can be disrupted either by conjugation with BODIPY or via Pd complexation. Given that both of these modifications lead to complexes with superior log*D* values when compared to **5b**, the loss of lysosomal specificity can be attributed to increased lipophilicity. This is further supported by the lysosomal preference of the relatively lipophobic Au(III) complex **5a**, which accumulates in lysosomes at the 6‐hour time point. Although previous research has suggested that greater lipophilicity facilitates lysosomal accumulation, this effect is typically observed in molecules containing basic functional motifs that, upon entering lysosomes, become protonated in the acidic environment and get trapped.^[^ ^38^, ^39^ ^]^ In the context of this work, the herein described molecules that have their Lewis basic binding motifs blocked with metal ions lack the ability to be protonated and may therefore easily diffuse out of the lysosome even if they previously entered the organelle. By comparison, **5b**’s lower lipophilicity may contribute to its retention in lysosomes. Alternatively, the differences in chloride lability, as witnessed during the measurement of the mass spectra, could be influencing the subcellular distributions.

## 3 Conclusion

In summary, we explored the interplay of metal identity and fluorophore conjugation on the localization patterns of Dp44mT‐derived square‐planar metal complexes. To this end, we used our reported coumarin conjugate of Dp44mT as ligand to synthesize novel Au(III) and Pd(II) complexes and compared them to the lysosome‐localizing Pt(II) complex. Additionally, a second set of similar metal complexes was synthesized with BODIPY as the conjugated fluorophore to assess the influence of using a different dye on the intracellular localization. Notably, the BODIPY‐conjugated metal complexes demonstrated higher log*D* values than their coumarin analogues, with the Pd(II) complexes being the most lipophilic and the Au(III) complexes being the least, in both sets. While BODIPY conjugation, like coumarin, did not alter the lysosomal accumulation of Dp44mT, the corresponding Pt(II) complex lost the preference for lysosomes, displaying a diffuse cytosolic distribution and enhanced in vitro activity, which stands in contrast to the coumarin analog. Subsequent XRF imaging of the Pt(II) complexes further substantiated these findings. Furthermore, complexing Pd(II) had a similar effect on both conjugates, as they appeared distributed across the cytoplasm. By contrast, the Au(III) complexes, which were unstable in cell culture medium, exhibited intracellular behavior like the metal‐free ligands, localizing and enlarging the lysosomes after 24 hours. However, the coumarin‐conjugated Au(III) complex exhibited improved lysosomal targeting after a shorter incubation of only 6 hours, suggesting that the Au(III) might still be coordinated at this earlier timepoint. Overall, these observations highlight the impact of lipophilicity and the role of the metal center in determining the localization preference of a metal complex in cells. Although no consistent relationship between the metal identity and the localization preference was established, increased lipophilicity correlated with a loss of organelle specificity. Nevertheless, these results underscore the importance of screening fluorophores and metal centers when designing new metal‐based organelle‐targeting imaging tools. Further studies in our lab will expand on these findings with other ligands to understand metal‐dependent subcellular localization patterns.

## Supporting Information

Deposition numbers 2 464 448 (for **8a**), 2 427 619 (for **8b**), and 2 464 449 (for **8c**) contain the supplementary crystallographic data for this paper. These data are provided free of charge by the joint Cambridge Crystallographic Data Centre and Fachinformationszentrum Karlsruhe Access Structures service. The Supporting Information contains full experimental procedures, characterizing data for all compounds, NMR and HR‐MS spectra, crystallographic data, stability studies, cell culture details, and confocal microscopy, as well as details on the X‐ray fluorescence investigations. The authors have cited additional references within the Supporting Information.^[^ ^40^, ^41^, ^42^, ^43^, ^44^, ^45^, ^46^, ^47^, ^48^, ^49^, ^50^ ^]^

## Conflict of Interest

The authors declare no conflict of interest.

## Supporting information



Supporting Information

## Data Availability

The data that support the findings of this study are available in the supplementary material of this article.
