# Supplementary material for: Investigating Metal and Fluorophore Controlled Intracellular Localization in Noble Metal Thiosemicarbazone Complexes
Source: Chemistry. 2025 Nov 5;31(69):e02613. doi: 10.1002/chem.202502613 (PMC12699179; doi:10.1002/chem.202502613)
Supplement: Supplementary file 1 — Supporting Information [file CHEM-31-e02613-s001.pdf]

Supporting information

Influence of Metal Identity and Fluorophore Conjugation on the Intracellular Localization of Isostructural Noble Metal Complexes of the Thiosemicarbazone Dp44mT

Nandan Sheernaly,[a] Axel Steinbrueck,[a] Nicolas Krahn,[a] Christoph Rumancev,[b] Frank Peeters,[c] Lejla Jusufagic,[b] Jasmine Ochs,[a] Jan Garrevoet,[d] Gerald Falkenberg,[d] Axel Rosenhahn,[b] and Nils Metzler-Nolte*[a]

[a] Ruhr University Bochum, Faculty of Chemistry and Biochemistry, Inorganic Chemistry I – Bioinorganic Chemistry, Universitaetsstrasse 150, 44801 Bochum, Germany.

[b] Ruhr University Bochum, Faculty of Chemistry and Biochemistry, Analytical Chemistry – Biointerfaces, Universitaetsstrasse 150, 44801 Bochum, Germany

[c] Ruhr University Bochum, Applied Microbiology, Faculty of Biology and Biotechnology, Universitätsstrasse 150, 44780 Bochum, Germany

[d] Deutsches Elektronen-Synchrotron DESY, Notkestrasse 85, 22607 Hamburg, Germany

Email address:

nils.metzler-nolte@ruhr-uni-bochum.de

Contents

1. List of Abbreviations 2
2. General remarks 4
3. Chemical Syntheses 6
4. Spectroscopic Information 12
5. Crystallographic data 29
6. Stability Studies 31
7. Lipophilicity (log$D_{7.2}$) 38
8. Cell viability studies 38
9. Uptake assay 40
10. Fluorescence spectroscopy 41
11. Confocal microscopy 42
12. X-ray fluorescence imaging (HeLa) 46
13. References 50

1. List of Abbreviations

an.	anhydrous
aq.	aqueous
AcOH	acetic acid
BODIPY	boron-dipyrromethene
conc.	concentrated
calcd	calculated
DCM	dichloromethane
DIPEA	*N,N*-diisopropylethylamine
DMF	*N,N*-dimethylformamide
DMSO	dimethylsulfoxide
Dp44mT	di-2-pyridylketone-4,4-dimethyl-3-thiosemicarbazone
DpT	di-2-pyridyl-thiosemicarbazone
EDC·HCl	1-ethyl-3-(3-dimethylaminopropyl)carbodiimide hydrochloride
eq.	equivalent
ESI	electronspray ionisation
Et_2O	diethyl ether
EtOAc	ethyl acetate
EtOH	ethanol
HATU	azabenzotriazole tetramethyl uronium hexafluorophosphate
HOBt	hydroxybenzotriazole
HRMS	high-resolution mass spectrometry

IC_{50}	half maximal inhibitory concentration
ICP-MS	inductively coupled plasma mass spectrometry
m/z	mass-to-charge ratio
Me	methyl
MeCN	acetonitrile
MeOH	methanol
MTT	3-(4,5-dimethylthiazol-2-yl)-2,5-diphenyltetrazolium bromide
NMR	nuclear magnetic resonance spectroscopy
o/n	overnight
PBS	phosphate-buffered saline
PMT	photomultiplier
rt	room temperature
sat.	saturated
SD	standard deviation
SMD	single molecule detection
TEA	triethylamine
TFA	trifluoroacetic acid
THF	tetrahydrofuran
TLC	thin layered chromatography
TMS	tetramethylsilane
UV	ultra-violet
Vis	visible
WLL	white light laser
XRF	X-ray fluorescence

2. General remarks

Moisture sensitive reactions

All moisture-sensitive reactions were carried out in a heated glass apparatus and under a protective argon atmosphere. To ensure a constant protective atmosphere, conventional Schlenk techniques were employed: the apparatuses were sealed with rubber septa, and rubber balloons filled with argon were placed. Solvents and liquid reagents were added through a septum via argon-flushed disposable syringes and cannulas, while solids were introduced into the reaction under the counterflow of argon.

Solvents and Reagents

Reagents have been sourced from reputable suppliers at the highest commercially available quality and used without further purification unless specified otherwise. HATU, 3-(1H-pyrrol-2-yl)propanoate, 3,5-dimethylpyrrole-2-carboxaldehyde, coumarin-343, Na_2PdCl_4, and K_2PtCl_4 were obtained from BLD-Pharm; EDC·HCl from Carbolution, HOBt, DIPEA, and γ-aminobutyric acid from Merck; $HAuCl_4$ from Thermofischer; and triphenylphosphine from abcr GmbH. Dry solvents (THF, DCM) were taken from a solvent drying system from MBraun (SPS-800) and stored over molecular sieves (3 Å) under argon. DMF was dried by storing it over molecular sieves (3 Å). Solvents for column chromatography and moisture-insensitive reactions (MeOH, DCM, EtOAc, heptane, isopropanol) were sourced of analytical grade and used without further purification.

Thin film and column chromatography

The reaction progress was controlled by thin-film chromatography (TLC) on silica gel-coated aluminum plates (Merck, silica gel 60, F254). As TLC staining reagents, potassium permanganate solution (1.0 g $KMnO_4$, 5 g K_2CO_3, 2 mL 5% (w/v) aqueous NaOH solution, 150 mL H_2O) was used. Chromatographic purification of reaction products was performed using a Büchi Pure C-815 Flash system. For silica or alumina column chromatography, FlashPure EcoFlex (Büchi) columns in the size 4 g or CHROMABOND Flash columns (Macherey-Nagel) in the sizes 15 g, 25 g, and 40 g were used depending on the amount of substance.

NMR spectroscopy

The NMR spectroscopic examinations of the synthesized substances with respect to their ^{1}H and ^{13}C nuclei were carried out on the Bruker devices aviii-700 (700 MHz), neo-400 (400 MHz) or aviii-300 (300 MHz). Chloroform ($CDCl_3$), DMSO (DMSO-d_6), methanol (MeOD-d_4), and D_2O from Deutero GmbH were used as NMR solvents. The recorded spectra were calibrated relative to the residual solvent reference peak in addition to TMS being employed as the internal standard ($CDCl_3$: δ (^{1}H) = 7.26 ppm and δ (^{13}C) = 77.16 ppm; DMSO-d_6: δ (^{1}H) = 2.50 ppm and δ (^{13}C) = 39.52 ppm; MeOD-d_4: δ (^{1}H) = 3.31 ppm and δ (^{13}C) = 49.00 ppm; D_2O: δ (^{1}H) = 4.79 ppm). The program MestreNova 14.0 was used to process the spectra. The following abbreviations were used to describe signal multiplicity: s = singlet, d = doublet, t = triplet, q = quartet, p = pentet, st = sextet, m = multiplet, and appropriate combinations thereof. The chemical shifts are expressed in parts per million (ppm) and the coupling constants *J* in Hertz (Hz).

Mass spectrometry

HR-MS: A Vion IMS QToF instrument (Waters Corp.) with an ESI source in positive sensitivity mode was used to measure high-resolution mass spectra. Prior to mass spectrometry, samples were injected into an Acquity I-Class UPLC system using a Nucleodur ISIS C18 HPLC precolumn (Macherey Nagel) with a flow of 0.6 ml/min of 15% H_2O, 85% ACN, and 0.1% FA. Masses in a range of 50–2000 m/z were detected with 0.1 s per scan, and leucine enkephalin was injected as a reference mass every minute. The used parameters were as follows: capillary voltage: 0.8 kV, sample cone voltage: 40 V, source offset voltage: 80 V, cone gas flow: 50 L/h, desolvation gas flow: 1000 L/h, source temperature: 120 °C, desolvation temperature: 550 °C, collision gas: N_2, collision low energy:6 V, and collision high-energy ramp: 28–60 V.

UV/Vis spectroscopy

UV/Vis absorption spectra were recorded on a Jasco V-670 spectrophotometer operated at ambient temperature in single-use UV-cuvettes (1 cm; BRAND). Appropriate blank measurements of the respective solvents were recorded and subtracted from the resulting spectra.

Fluorescence spectroscopy

Fluorescence spectra were recorded on a Jasco FP-8300 spectrofluorometer. Emission and excitation slits were fixed at 5 nm, respectively, unless specified otherwise. Where applicable, respective blanks were subtracted from the spectra.

Crystal Structures

Single crystals suitable for diffraction experiments were analyzed using an XtaLAB SuperNova (Rigaku Oxford diffraction, Cu Ka I = 1.54184 Å) diffractometer. The crystals were handled in perfluorinated oil, mounted on fiber loops, and cooled by a flow of N_2 throughout the measurement. Data reduction was performed using the program package CrysAlis^{Pro} (Oxford diffraction). Structural solutions were computed using SHELXT[1] (intrinsic phasing) and refined against F using SHELXL.[2] The program package (OLEX)[3] served as graphical user interface.

Cell culture

Adherent HeLa cells were obtained from Leibnitz Institute Deutsche Sammlung von Mikroorganismen und Zellen (DSMZ) and maintained in Dulbecco's Modified Eagle's Medium (DMEM) containing 10% fetal bovine serum and 1% penicillin + streptomycin. Cells were kept under a humidified atmosphere of 10% CO_2 and 90% air and split when they reached 90% confluency.

3. Chemical Syntheses

Dp44mT-Fluorophore conjugates

Dp44mT-Coumarin conjugate (4)

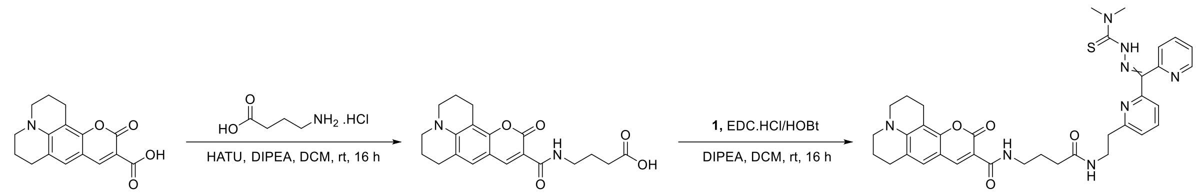

Coumarin 343 (1) **Dp44mT-Coumarin Conjugate (4)**

4 was synthesized according to our published procedure.[4]

Dp44mT-BODIPY conjugate (6)

BODIPY-FL (2) was synthesized according to a reported procedure.[5]

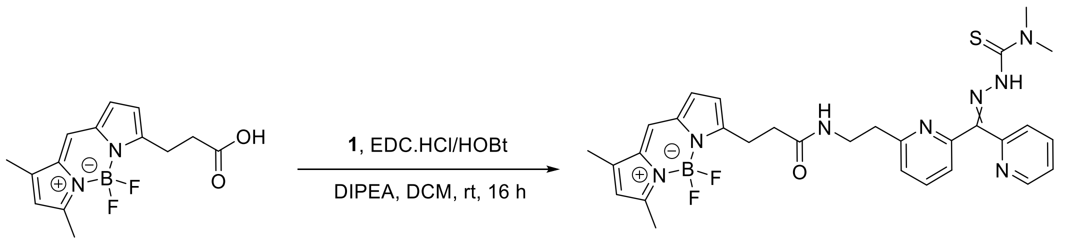

BODIPY-FL (2) **Dp44mT-BODIPY Conjugate (6)**

2 (0.50 g, 1.71 mmol, 1.0 eq.) was dissolved in dry DCM (40 mL), and EDC·HCl (0.66 g, 3.42 mmol, 2.0 eq.), HOBt (0.15 g, 3.42 mmol, 2.0 eq.) and DIPEA (0.92 mL, 5.14 mmol, 3.0 eq.) were sequentially added. The resulting reaction mixture was stirred at rt for 30 min before adding **1** (0.62 g, 1.88 mmol, 1.1 eq.), and the reaction was stirred overnight at rt. Subsequently, the reaction was diluted with sat. aq. NH_4Cl, and the organic phase was washed with water, sat. aq. NH_4Cl, and brine, dried over Na_2SO_4, and concentrated under reduced pressure. The crude residue was later purified via silica gel column chromatography (DCM:MeOH (100:0) → (95:5) v/v%) to obtain the desired product **6** as a red-colored solid (0.63 g, 1.04 mmol, 61% (Mixture of E/Z isomers (1:1)).

^{1}H NMR (400 MHz, $CDCl_3$): δ (ppm): 15.01 (s, 1H), 14.45 (s, 1H), 8.62 (dd, *J* = 5.0, 1.7 Hz, 1H), 8.56 (dd, *J* = 5.0, 1.7 Hz, 1H), 7.97 (d, *J* = 7.9 Hz, 1H), 7.88 (d, *J* = 7.9 Hz, 1H), 7.79 (tdd, *J* = 7.8, 3.7, 1.8 Hz, 2H), 7.70 – 7.58 (m, 3H), 7.39 (d, *J* = 8.0 Hz, 1H), 7.32 – 7.27 (m, 2H), 7.20 (d, *J* = 7.7 Hz, 1H), 7.11 – 7.04 (m, 3H), 6.81 (m, 2H), 6.50 (t, *J* = 5.7 Hz, 1H), 6.32 (t, *J* = 5.7 Hz, 1H), 6.18 (dd, *J* = 9.9, 4.1 Hz, 2H), 6.09 (d, *J* = 7.4 Hz, 2H), 3.69 (q, *J* = 6.5 Hz, 2H), 3.59 (p, *J* = 6.4 Hz, 2H), 3.43 (d, *J* = 14.3 Hz, 12H), 3.21 (t, *J* = 7.4 Hz, 2H), 3.16 (t, *J* = 7.4 Hz, 2H), 3.06 (t, *J* = 6.8 Hz, 2H), 2.91 (t, *J* = 6.2 Hz, 2H), 2.61 (t, *J* = 7.3 Hz, 2H), 2.52 (s, 6H), 2.37 (t, *J* = 7.5 Hz, 2H), 2.24 (s, 3H), 2.21 (s, 3H). **^{13}C NMR (101 MHz, $CDCl_3$)**: δ (ppm): 181.3, 181.2, 172.1, 171.6, 160.2, 160.0, 159.1, 158.1, 157.7, 157.6, 157.6, 156.6, 155.8, 152.1, 151.4, 148.4, 147.2, 143.9, 143.3, 142.7, 137.7, 137.6, 137.3, 137.2, 136.9, 135.1, 133.4, 128.5, 128.4, 127.0, 126.6, 126.4, 124.8, 124.4, 124.0, 124.0, 123.8, 123.4, 123.2, 122.7, 122.4, 120.4, 117.3, 117.2, 38.7, 38.5, 37.7, 37.6, 36.9, 36.5, 35.8, 35.5, 24.8, 24.7, 15.0, 11.5, 11.4. **HR-MS:** m/z found for $C_{30}H_{34}BF_2N_8OS$ $[M+H]^+$: 603.26254 (calcd m/z: 603.26374)

Functionalization of Dp44mT

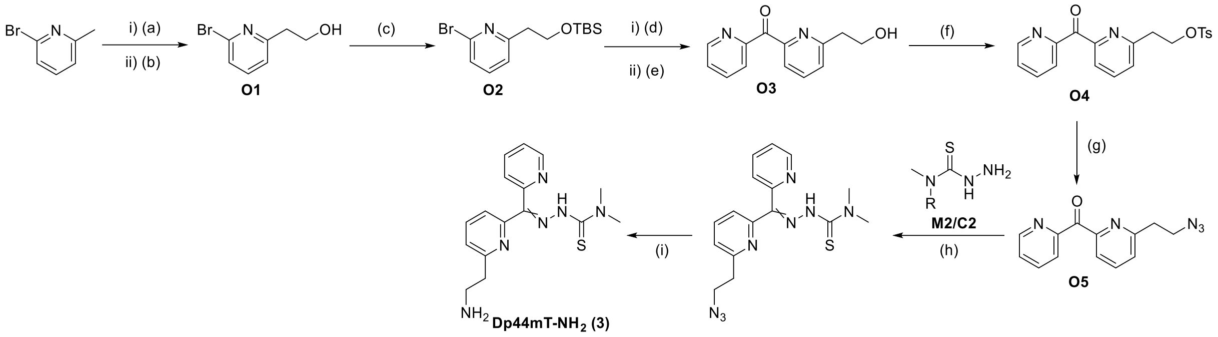

Scheme S1: Synthetic route leading to **Dp44mT-NH₂,** (a) LDA, DMF, THF, -78°C, 30 min; (b) acetic acid, MeOH, $NaBH_4$, rt, 16 h, 62%; (c) TBSCl, imidazole, DMF, rt, 16 h, 88%; (d) n-BuLi, 2-cyanopyridine, Et_2O, -78 °C, 30 min; (e) 1M HCl, 50 °C, 3 h, 42%; (f) TsCl, Et_3N, 3 h, 83%; (g) NaN_3, DMF, 80 °C, 16 h, 98%; (h) AcOH (few drops), EtOH, reflux, 1.5 h, 92%.(i) PPh_3, H_2O, THF, rt, 16 h, 68%.

Dp44mT-NH$_2$ (3) was synthesized according to our published synthetic route.[4,6] The subsequent steps are detailed below:

Metal complexes

Au complexes

Dp44mT-Coumarin Au complex (5a)

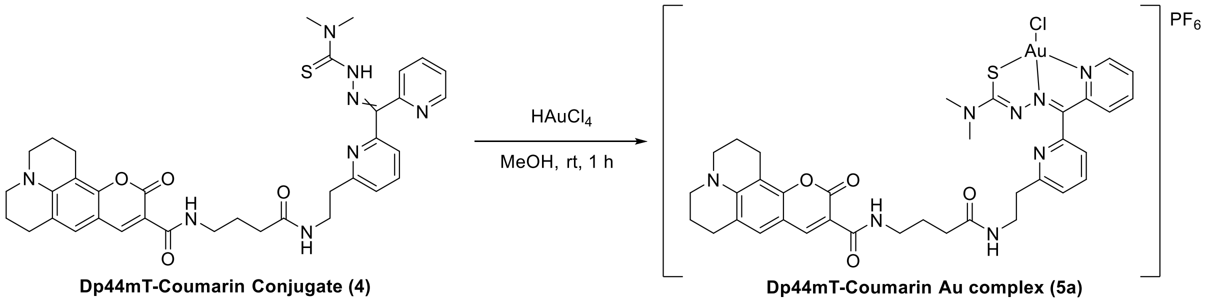

4 (75.0 mg, 110.16 µmol, 1.0 eq.) was dissolved in MeOH (10 mL), and a solution of tetrachloroauric(III) acid trihydrate (48.0 mg, 121.88 µmol, 1.1 eq.) in MeOH (2 mL) was added. The resulting reaction mixture was stirred at rt for 1 h. Subsequently, the precipitate formed was filtered and washed with copious amounts of cold methanol. The crude solid was then redissolved in MeOH (2 mL) and aq. NH_4PF_6 was added dropwise until the product started precipitating, and the resulting suspension was stirred for an hour. Subsequently, the precipitate was filtered, washed with water and methanol, and recrystallized from DCM/heptane to obtain the desired product **5a** as a yellow-colored solid. (16.2 mg, 15.32 µmol, 14%).

^{1}H NMR (400 MHz, DMSO-d$_6$): δ (ppm): 8.97 (d, *J* = 5.9 Hz, 1H), 8.62 – 8.53 (m, 1H), 8.47 (s, 1H), 8.41 (t, *J* = 8.0 Hz, 1H), 8.05 (d, *J* = 8.2 Hz, 1H), 7.97 (t, *J* = 7.0 Hz, 3H), 7.79 (d, *J* = 7.9 Hz, 1H), 7.50 (d, *J* = 7.7 Hz, 1H), 7.25 (s, 1H), 3.51 (q, *J* = 6.5 Hz, 2H), 3.33 (m, 4H), 3.26 (d, *J* = 20.6 Hz, 6H), 3.19 – 3.11 (m, 2H), 2.97 (t, *J* = 6.7 Hz, 2H), 2.72 (m, 4H), 2.05 (t, *J* = 7.5 Hz, 2H), 1.88 (m, 4H), 1.63 (p, *J* = 7.2 Hz, 2H). **^{13}C NMR (101 MHz, DMSO-d$_6$)**: δ (ppm): 206.5, 175.6, 171.6, 162.4, 161.8, 160.2, 157.4, 152.1, 151.1, 148.1, 147.4, 146.8, 145.3, 144.1, 137.4, 131.6, 127.9, 127.2, 125.9, 125.5, 119.5, 107.9,

107.3, 104.6, 54.9, 49.6, 49.0, 44.8, 38.4, 38.0, 37.4, 32.9, 31.3, 30.7, 28.4, 26.8, 25.4, 22.1, 20.5, 19.6, 14.0. **HR-MS:** m/z found for $C_{36}H_{39}AuClN_8O_4S$ [M-PF_6]$^+$: 911.21743 (calcd m/z: 911.216912)

Dp44mT-BODIPY Au complex (7a)

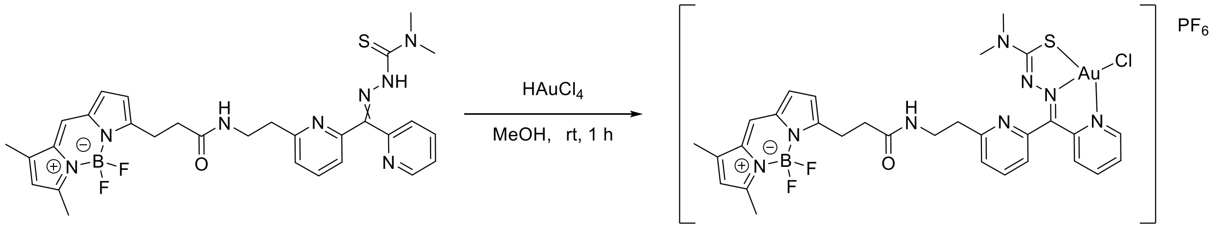

Dp44mT-BODIPY Conjugate (6) **Dp44mT-BODIPY Au complex (7a)**

6 (100.0 mg, 165.97 µmol, 1.0 eq.) was dissolved in MeOH (15 mL), and a solution of tetrachloroauric(III) acid trihydrate (72.0 mg, 182.82 µmol, 1.1 eq.) in MeOH (3 mL) was added. The resulting reaction mixture was stirred at rt for 1 h. Subsequently, the formed precipitate was filtered off and washed with copious amounts of cold methanol. The crude solid was then redissolved in MeOH (2 mL) and aq. NH_4PF_6 was added dropwise until the product started precipitating, and the resulting suspension was stirred for an hour. Subsequently, the precipitate was filtered and washed with water and methanol to obtain the desired product **7a** as a red-colored solid. (98.0 mg, 100.11 µmol, 63%).

^{1}H NMR (400 MHz, DMSO-d$_6$): δ (ppm): 8.94 (d, *J* = 5.6 Hz, 1H), 8.35 (t, *J* = 8.0 Hz, 1H), 8.05 (dd, *J* = 10.7, 5.0 Hz, 1H), 8.00 (d, *J* = 7.8 Hz, 1H), 7.94 (d, *J* = 8.1 Hz, 1H), 7.89 (t, *J* = 6.9 Hz, 1H), 7.81 (d, *J* = 7.8 Hz, 1H), 7.64 (s, 1H), 7.52 (d, *J* = 7.8 Hz, 1H), 6.99 (d, *J* = 4.0 Hz, 1H), 6.30 (s, 1H), 6.16 (d, *J* = 4.0 Hz, 1H), 3.59 – 3.51 (m, 2H), 3.27 (d, *J* = 17.6 Hz, 7H), 3.06 – 2.90 (m, 5H), 2.46 (s, 3H), 2.42 (t, *J* = 7.7 Hz, 2H), 2.25 (s, 3H). **^{13}C NMR (101 MHz, DMSO-d$_6$)**: δ (ppm): 175.6, 170.8, 160.1, 159.3, 157.5, 157.4, 151.1, 146.7, 145.4, 144.2, 144.0, 137.4, 134.5, 132.9, 131.4, 128.8, 127.7, 126.0, 125.5, 125.3, 120.4, 116.3, 44.8, 40.6, 38.1, 37.3, 33.7, 23.9, 14.5, 11.0. **HR-MS:** m/z found for $C_{30}H_{32}AuBClF_2N_8OS$ [M-PF_6]$^+$: 833.18516 (calcd m/z: 833.18350)

Dp44mT-Au complex (8a)

Dp44mT was synthesized according to our reported procedure.[6]

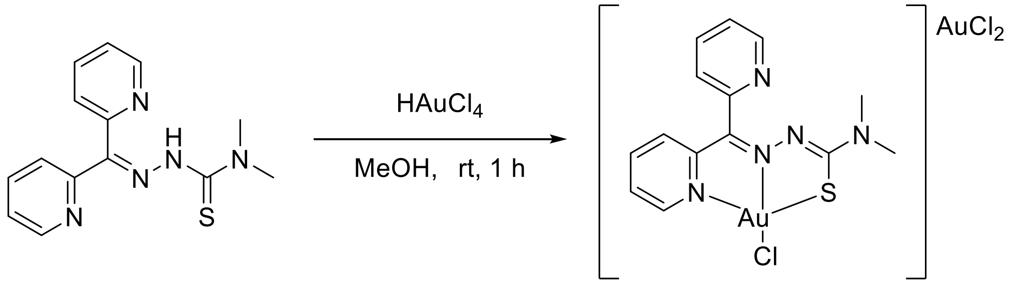

Dp44mT **Dp44mT-Au complex (8a)**

Dp44mT (100.0 mg, 350.42 µmol, 1.0 eq.) was dissolved in MeOH (12.5 mL), and a solution of tetrachloroauric(III) acid trihydrate (140.0 mg, 355.46 µmol, 1.1 eq.) in MeOH (3 mL) was added. The resulting reaction mixture was stirred at rt for 1 h. Subsequently, the formed precipitate was filtered off and washed with copious amounts of cold methanol to obtain the desired product **8a** as a brown-colored solid. (157.0 mg, 200.09 µmol, 57%).

^{1}H NMR (400 MHz, DMSO-d$_6$): δ (ppm): 9.02 (d, *J* = 5.7 Hz, 1H), 8.88 (d, *J* = 4.9 Hz, 1H), 8.45 – 8.36 (m, 1H), 8.12 (td, *J* = 7.8, 1.8 Hz, 1H), 8.03 – 7.96 (m, 2H), 7.92 (d, *J* = 8.1 Hz, 1H), 7.69 (dd, *J* = 7.6, 4.8 Hz, 1H), 3.28 (d, *J* = 14.7 Hz, 6H). **^{13}C NMR (101 MHz, DMSO-d$_6$)**: δ (ppm): 175.7, 157.3, 150.9, 150.1, 146.9, 146.0, 144.2, 137.3, 131.2, 127.9, 127.8, 126.2, 48.6, 44.8, 40.7. **HR-MS:** m/z found for $C_{14}H_{14}AuClN_5S$ $[M-AuCl_2]^+$: 516.03289 (calcd m/z: 516.03241)

Pt complexes

Dp44mT-Coumarin Pt complex (5b)

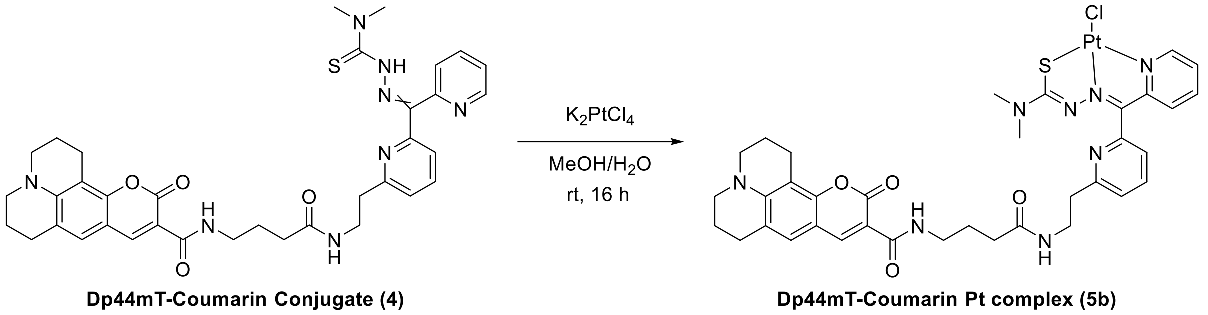

5b used in this study was synthesized previously.[4]

Dp44mT-BODIPY Pt complex (7b)

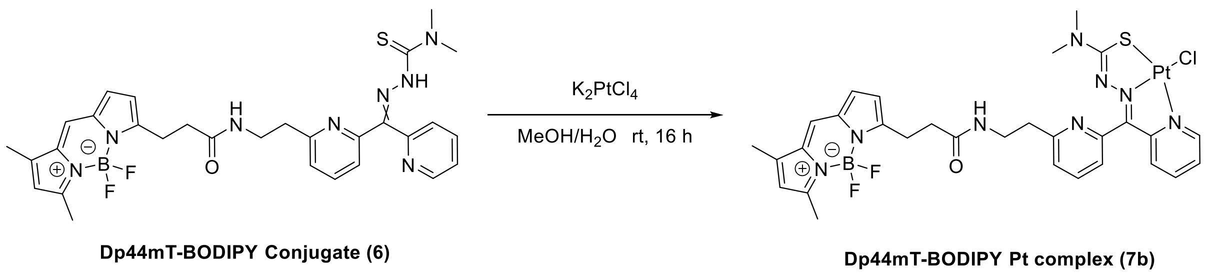

6 (100.0 mg, 165.97 µmol, 1.0 eq.) was dissolved in MeOH (16 mL), and a solution of potassium tetrachloroplatinate(II) (75.78 mg, 182.57 µmol, 1.1 eq.) in H_2O (4 mL) was added. The resulting reaction mixture was stirred at rt for 16 h. Subsequently, the formed precipitate was filtered off and washed with copious amounts of water and methanol to obtain the desired product as a dark red solid (55.0 mg, 66.10 µmol, 40%).

^{1}H NMR (700 MHz, DMSO-d$_6$): δ (ppm): 8.80 (d, *J* = 5.9 Hz, 1H), 8.06 (d, *J* = 6.2 Hz, 1H), 8.00 (t, *J* = 8.5 Hz, 1H), 7.88 (t, *J* = 8.2 Hz, 1H), 7.75 (d, *J* = 7.9 Hz, 1H), 7.62 (d, *J* = 10.3 Hz, 2H), 7.48 (d, *J* = 8.7 Hz, 1H), 7.37 (d, *J* = 8.3 Hz, 1H), 7.01 (d, *J* = 4.1 Hz, 1H), 6.28 (s, 1H), 6.20 (d, *J* = 4.2 Hz, 1H), 3.50 (q, *J* = 7.0 Hz, 2H), 3.27 (s, 3H), 3.20 (s, 3H), 3.03 (t, *J* = 8.2 Hz, 2H), 2.95 (t, *J* = 7.4 Hz, 2H), 2.46 (s, 3H), 2.43 (m, 2H), 2.25 (s, 3H). **^{13}C NMR (101 MHz, DMSO-d$_6$)**: δ (ppm): 185.4, 171.3, 159.9, 159.6, 159.4, 158.2, 146.9, 144.5, 140.7, 137.2, 134.9, 133.4, 129.4, 127.6, 126.4, 125.7, 124.6, 120.7, 116.9, 55.4, 42.6, 38.8, 37.7, 34.1, 31.7, 31.2, 24.4, 22.6, 15.0, 14.4, 11.5. **HR-MS:** m/z found for $C_{30}H_{33}BClF_2N_8OPtS$ $[M+H]^+$: 832.18993 (calcd m/z: 832.18955)

Dp44mT-Pt complex (8b)

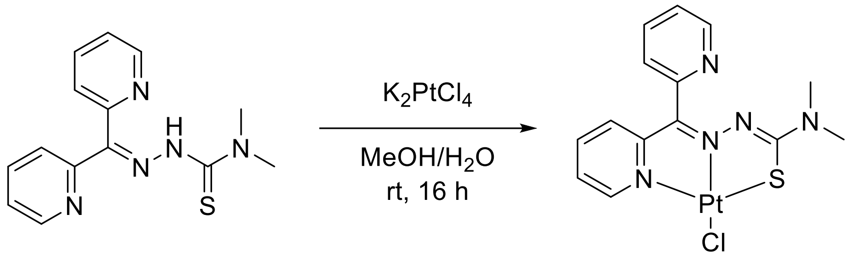

Dp44mT **Dp44mT-Pt Complex (8b)**

8b used in this study was synthesized previously.[4]

Pd complexes

Dp44mT-Coumarin Pd complex (5c)

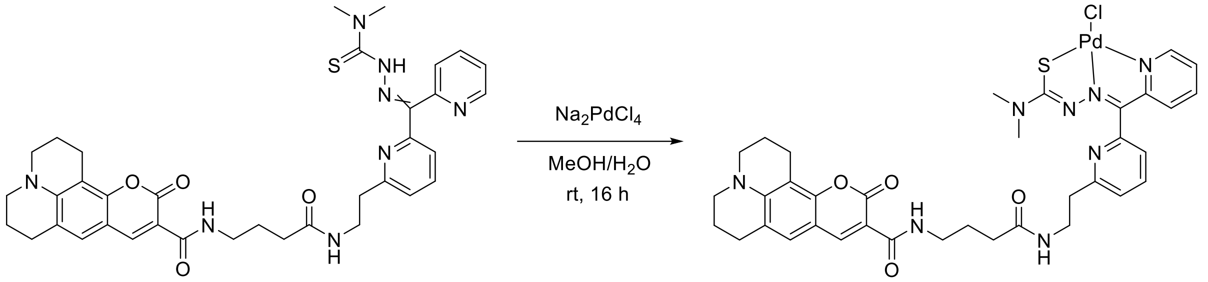

Dp44mT-Coumarin Conjugate (4) **Dp44mT-Coumarin Pd complex (5c)**

4 (100.0 mg, 146.88 µmol, 1.0 eq.) was dissolved in MeOH (15 mL), and a solution of sodium tetrachloropalladate(II) (56.0 mg, 160.82 µmol, 1.1 eq.) in H_2O (3 mL) was added. The resulting reaction mixture was stirred at rt for 16 h. Subsequently, the formed precipitate was filtered off and washed with copious amounts of water and methanol. The crude solid was then purified via alumina column chromatography (DCM:MeOH 100:0 v/v% → 95:5 v/v%) to obtain the desired product as an orange solid (10.0 mg, 12.19 µmol, 8%).

^{1}H NMR (400 MHz, DMSO-d$_6$): δ (ppm): 8.61 – 8.54 (m, 2H), 8.45 (s, 1H), 8.00 (td, *J* = 7.9, 1.7 Hz, 1H), 7.92 (t, *J* = 5.8 Hz, 1H), 7.85 (t, *J* = 7.9 Hz, 1H), 7.65 (d, *J* = 7.8 Hz, 1H), 7.58 (ddd, *J* = 7.4, 5.5, 1.3 Hz, 1H), 7.49 (d, *J* = 8.1 Hz, 1H), 7.34 (q, *J* = 7.5 Hz, 1H), 7.23 (s, 1H), 3.45 (q, *J* = 6.7 Hz, 2H), 3.20 (q, *J* = 6.7 Hz, 1H), 3.14 (s, 3H), 3.06 (s, 3H), 2.91 (t, *J* = 7.0 Hz, 2H), 2.72 (q, *J* = 5.8 Hz, 4H), 2.05 (t, *J* = 7.5 Hz, 2H), 1.88 (m, 4H), 1.65 (p, *J* = 7.3 Hz, 2H). **^{13}C NMR (101 MHz, DMSO-d$_6$)**: δ (ppm): 181.3, 171.6, 162.5, 161.8, 159.1, 158.3, 152.0, 150.1, 148.0, 148.0, 147.5, 147.4, 140.4, 136.5, 127.1, 126.7, 125.3, 125.2, 124.1, 119.5, 107.9, 107.4, 104.6, 54.9, 49.6, 49.0, 38.4, 38.2, 37.4, 32.9, 31.3, 28.4, 26.8, 25.4, 22.6, 22.1, 20.5, 19.6, 14.0. **HR-MS:** m/z found for $C_{36}H_{39}PdN_8O_4S$ [M-Cl]$^+$: 785.18573 (calcd m/z: 785.18497)

Dp44mT-BODIPY Pd complex (7c)

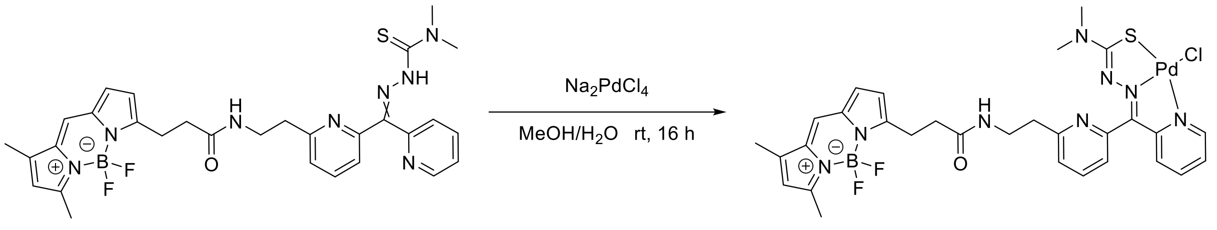

Dp44mT-BODIPY Conjugate (6) **Dp44mT-BODIPY Pd complex (7c)**

A procedure similar to that used for **5c** was employed for the synthesis of **7c**. Yield: 14.0 mg, 18.83 µmol, 11% (from 100.0 mg of **6**)

^{1}H NMR (400 MHz, DMSO-d$_6$): δ (ppm): 8.57 (dd, *J* = 5.4, 1.6 Hz, 1H), 8.05 – 7.92 (m, 2H), 7.87 (td, *J* = 7.8, 1.9 Hz, 1H), 7.70 (d, *J* = 7.7 Hz, 1H), 7.64 (s, 1H), 7.58 – 7.50 (m, 1H), 7.45 (d, *J* = 8.1 Hz, 1H), 7.35 (d, *J* = 7.7 Hz, 1H), 7.02 (d, *J* = 4.0 Hz, 1H), 6.29 (s, 1H), 6.21 (d, *J* = 4.0 Hz, 1H), 3.49 (q, *J* = 6.5 Hz, 2H), 3.15 (s, 3H), 3.07 (s, 3H), 3.03 (t, *J* = 7.8 Hz, 2H), 2.93 (t, *J* = 6.9 Hz, 2H), 2.46 (s, 4H), 2.42 (d, *J* = 7.6 Hz, 2H), 2.26 (s, 3H). **^{13}C NMR (101 MHz, DMSO-d$_6$)**: δ (ppm): 181.3, 170.7, 169.1, 159.1, 159.1, 158.4, 158.3, 157.7, 150.1, 148.1, 148.0, 147.5, 144.1, 140.4, 136.5, 134.4, 132.9, 128.9, 126.7, 125.3, 125.2, 124.2, 124.1, 120.3, 116.4, 54.9, 42.5, 38.3, 37.3, 33.7, 31.3, 23.9, 22.6, 22.1, 14.5, 14.0, 11.0. **HR-MS:** m/z found for $C_{30}H_{32}BF_2N_8OPdS$ $[M-Cl]^+$: 707.15234 (calcd m/z: 707.15157)

Dp44mT-BODIPY Pd complex (8c)

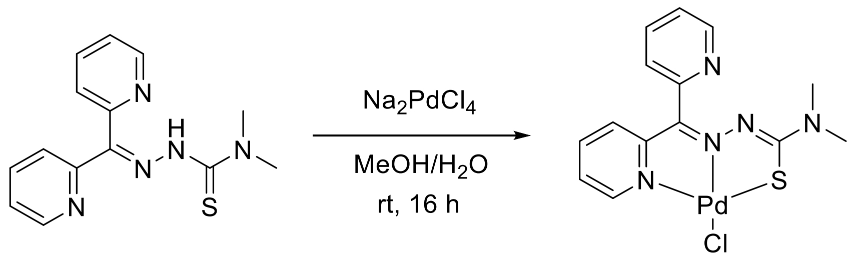

Dp44mT **Dp44mT-Pd Complex (8c)**

A procedure similar to that used for **5c** was employed for the synthesis of **8c**. Yield: 34.0 mg, 79.77 µmol, 46% (from 50.0 mg of **Dp44mT**)

^{1}H NMR (400 MHz, DMSO-d$_6$): δ (ppm): 8.76 (d, *J* = 4.8 Hz, 1H), 8.61 (d, *J* = 5.3 Hz, 1H), 8.03 (td, *J* = 8.0, 1.7 Hz, 1H), 7.97 (td, *J* = 7.8, 1.7 Hz, 1H), 7.85 (d, *J* = 7.9 Hz, 1H), 7.61 (dd, *J* = 7.7, 5.5 Hz, 1H), 7.53 (dd, *J* = 7.5, 4.9 Hz, 1H), 7.38 (d, *J* = 8.1 Hz, 1H), 3.16 (s, 3H), 3.06 (s, 3H). **^{13}C NMR (101 MHz, DMSO-d$_6$)**: δ (ppm): 181.4, 158.2, 149.9, 149.4, 148.2, 140.6, 136.4, 127.5, 126.4, 125.4, 124.7. **HR-MS:** m/z found for $C_{14}H_{14}PdN_5S$ $[M-Cl]^+$: 390.00114 (calcd m/z: 390.00047)

4. Spectroscopic Information

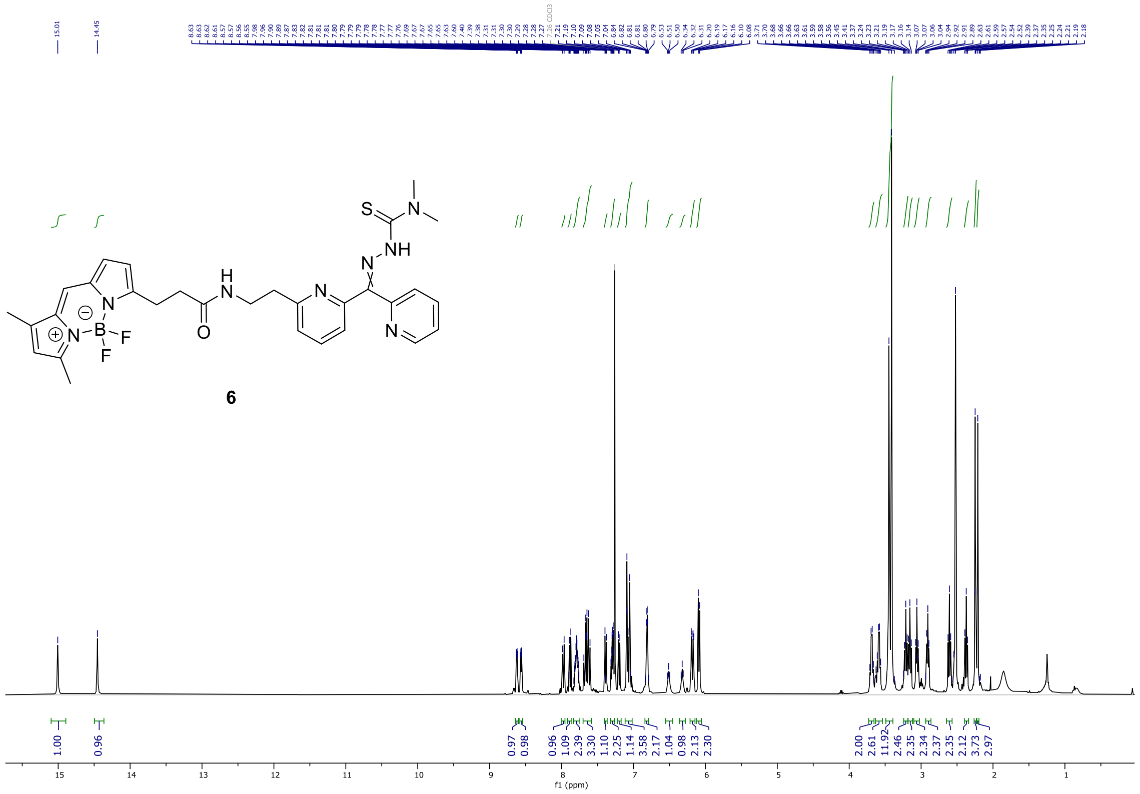

Figure S1: ^{1}H NMR Spectrum of **6.**

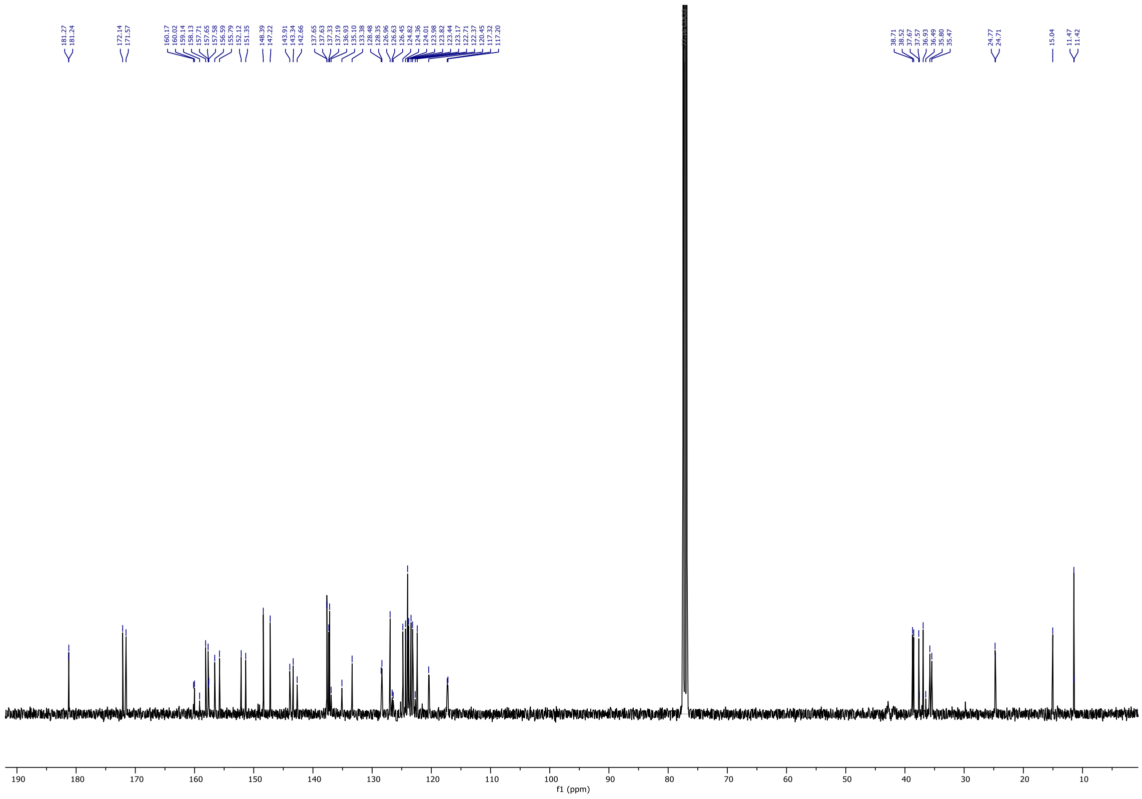

Figure S2: ^{13}C NMR Spectrum of **6.**

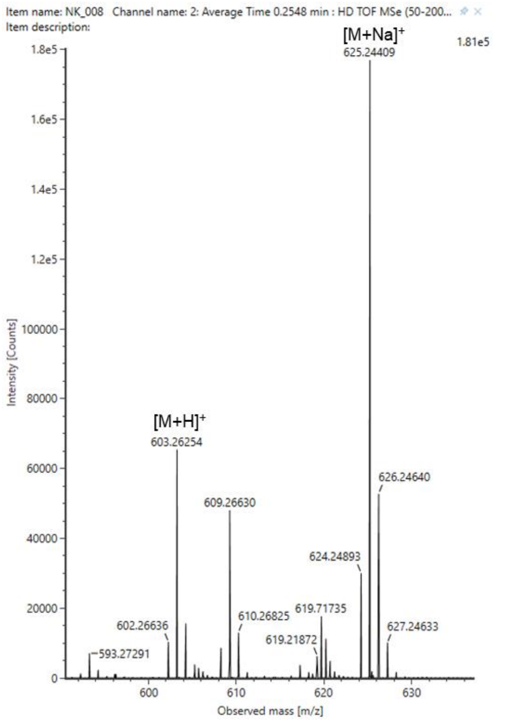

Figure S3: Zoomed-in HRMS of **6.**

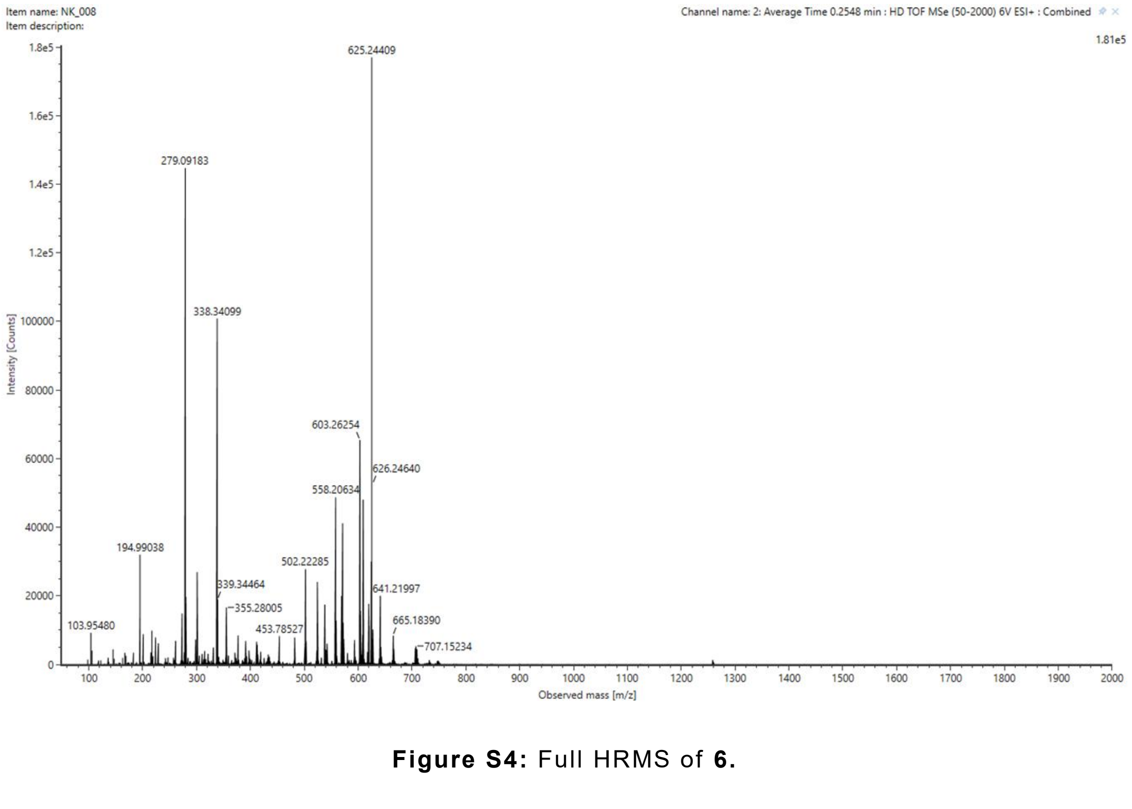

Figure S4: Full HRMS of **6.**

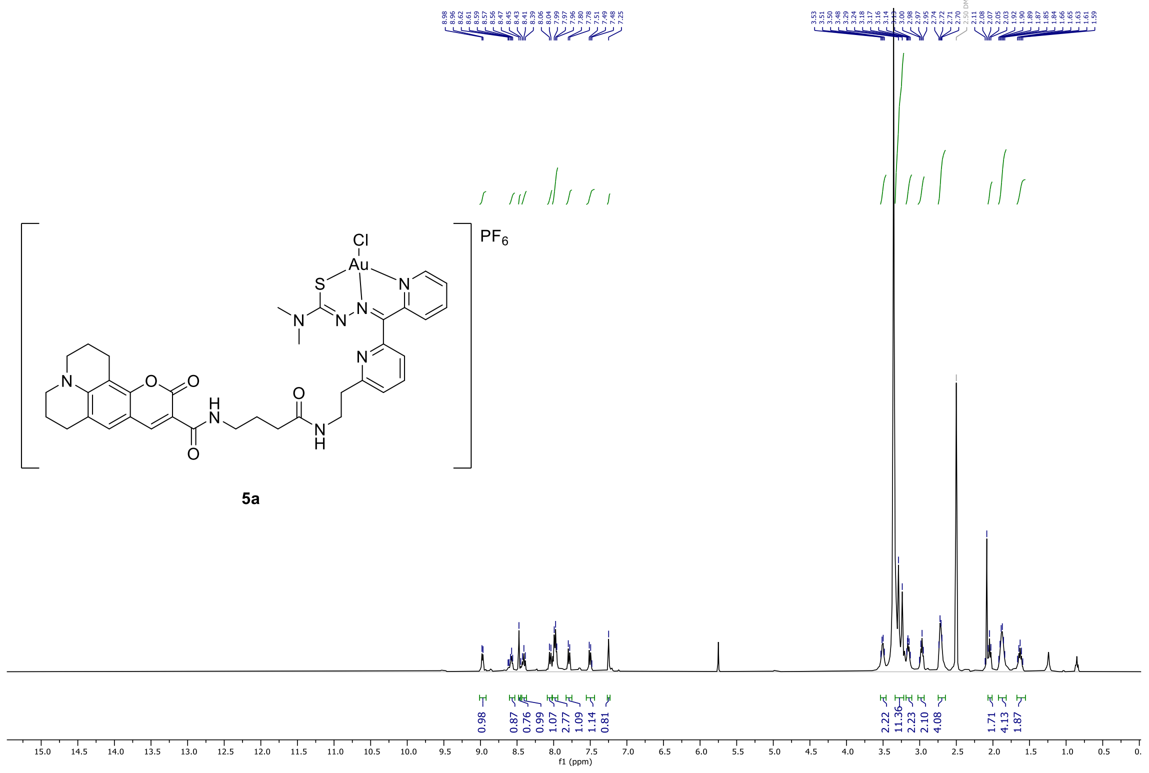

Figure S5: ^{1}H NMR Spectrum of **5a.**

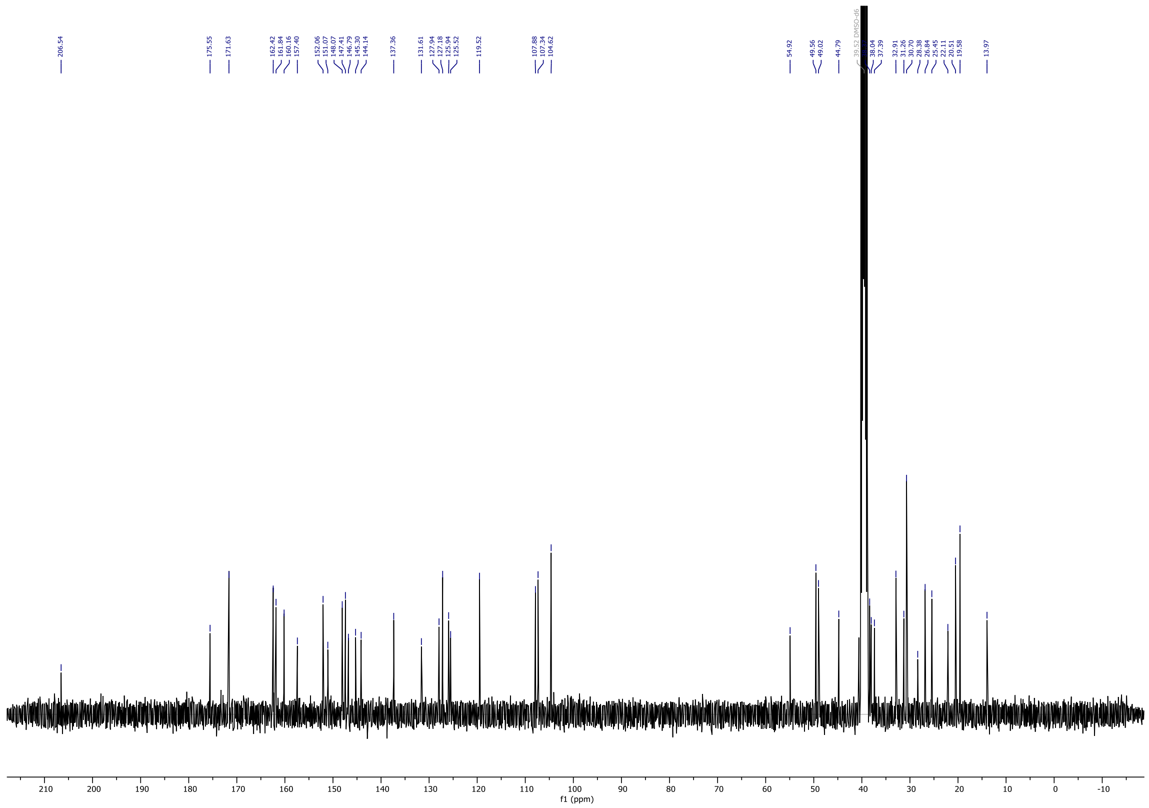

Figure S6: ^{13}C NMR Spectrum of **5a.**

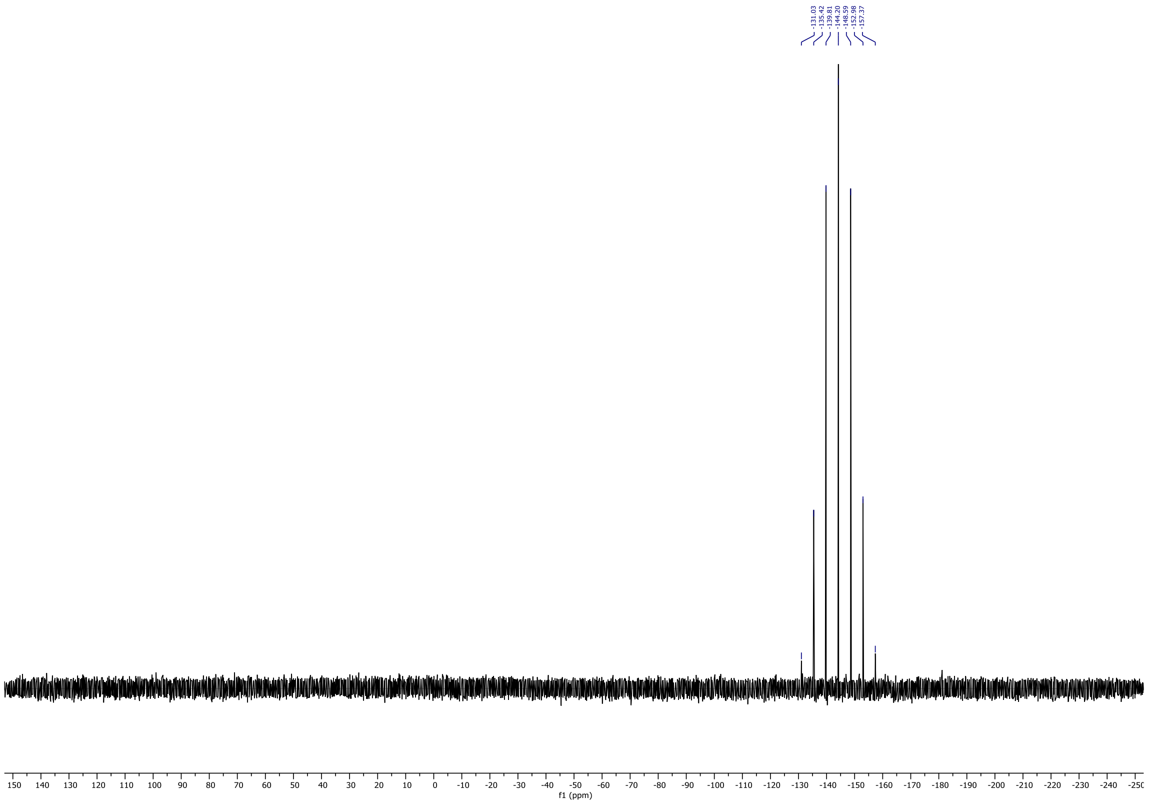

Figure S7: ^{31}P NMR Spectrum of **5a.**

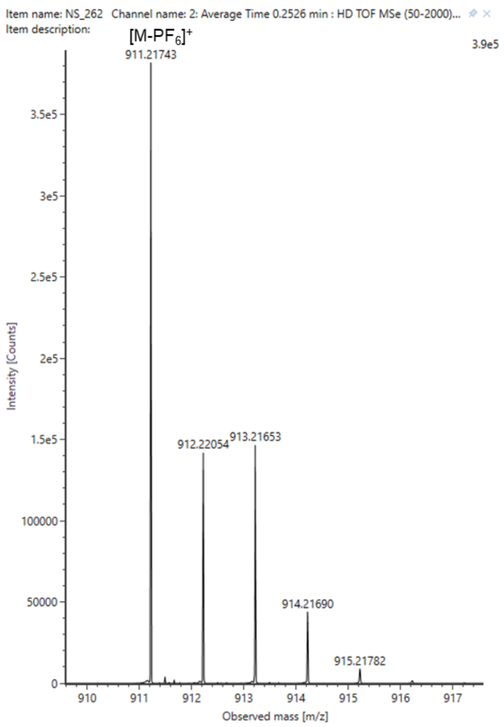

Figure S8: Zoomed-in HRMS of **5a.**

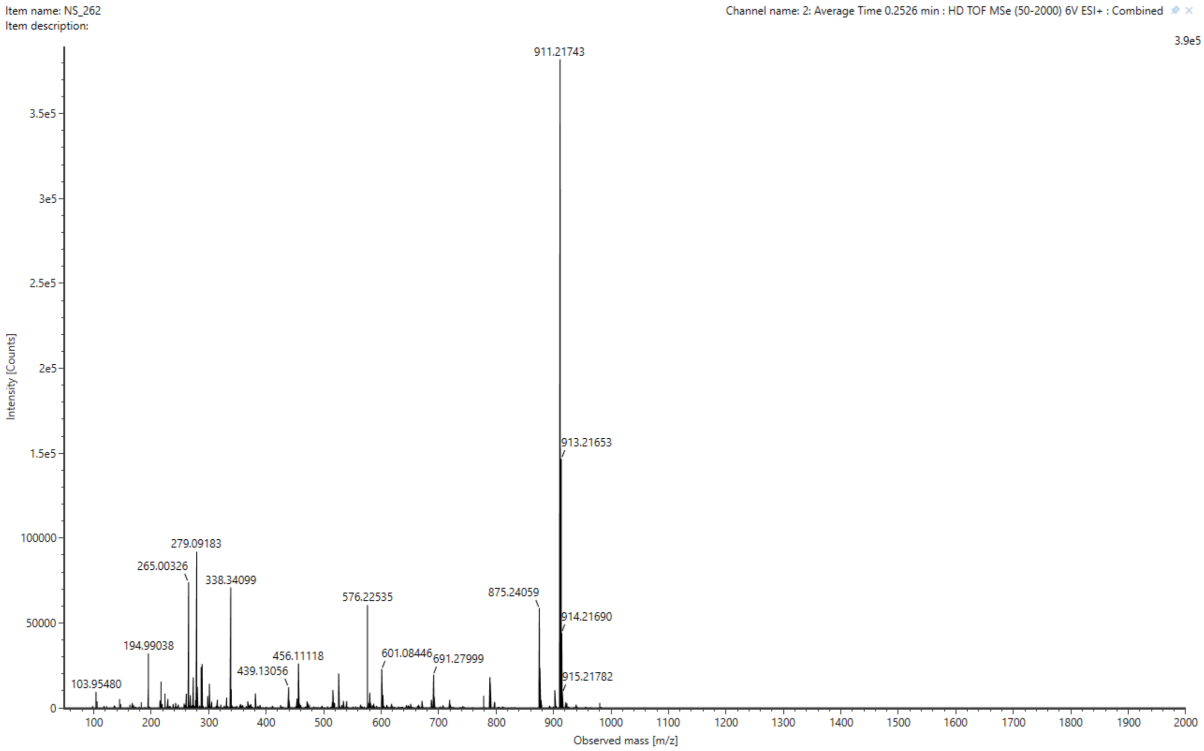

Figure S9: Full HRMS of **5a**.

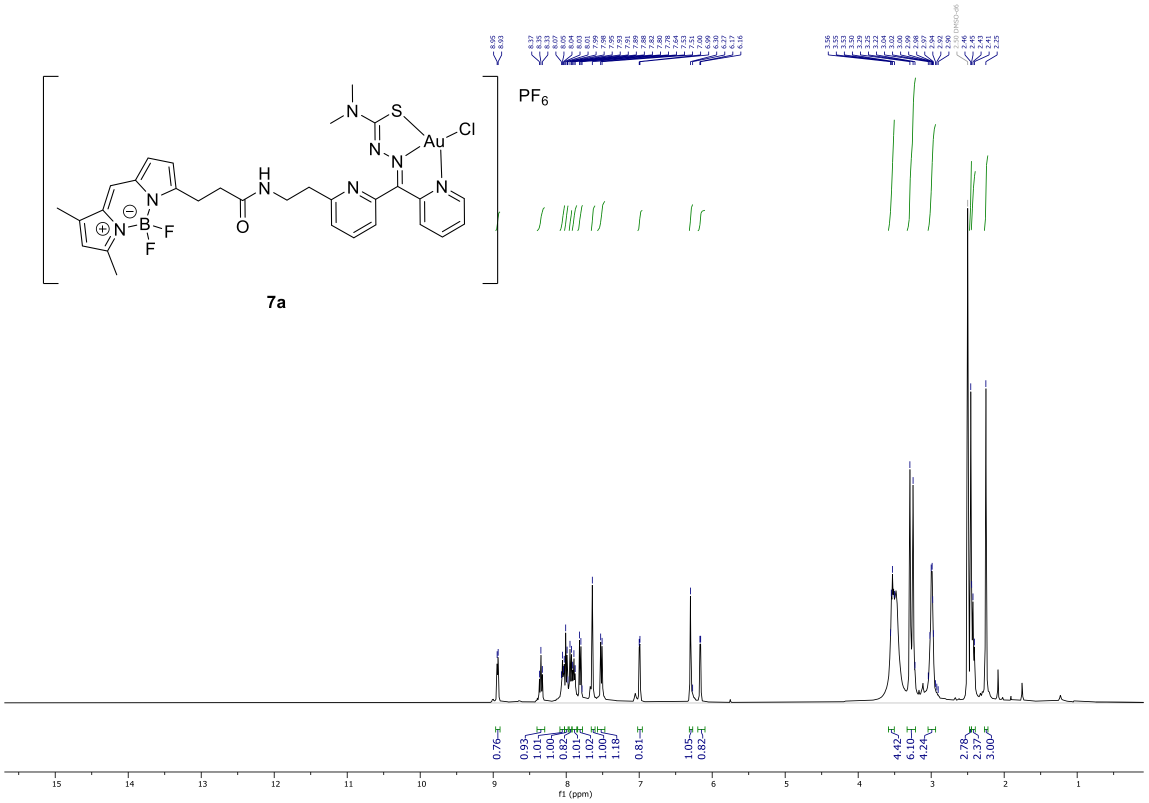

Figure S10: ^{1}H NMR Spectrum of **7a**.

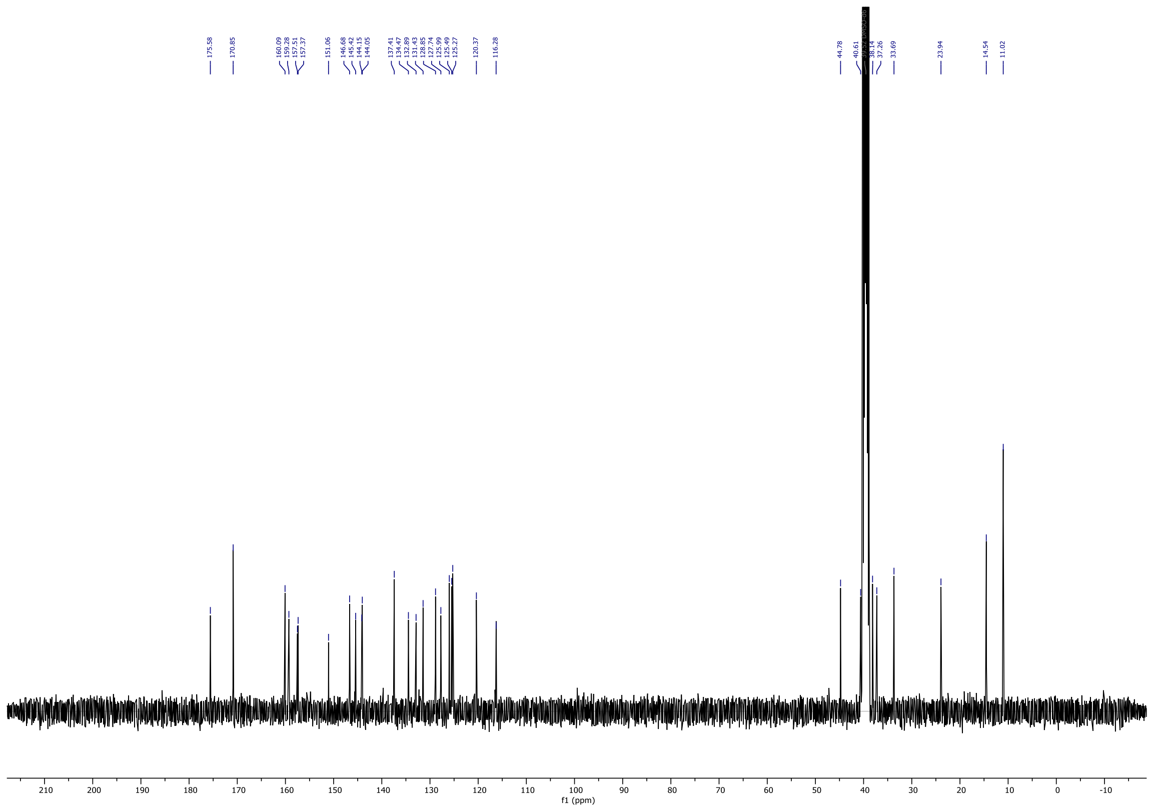

Figure S11: ^{13}C NMR Spectrum of **7a.**

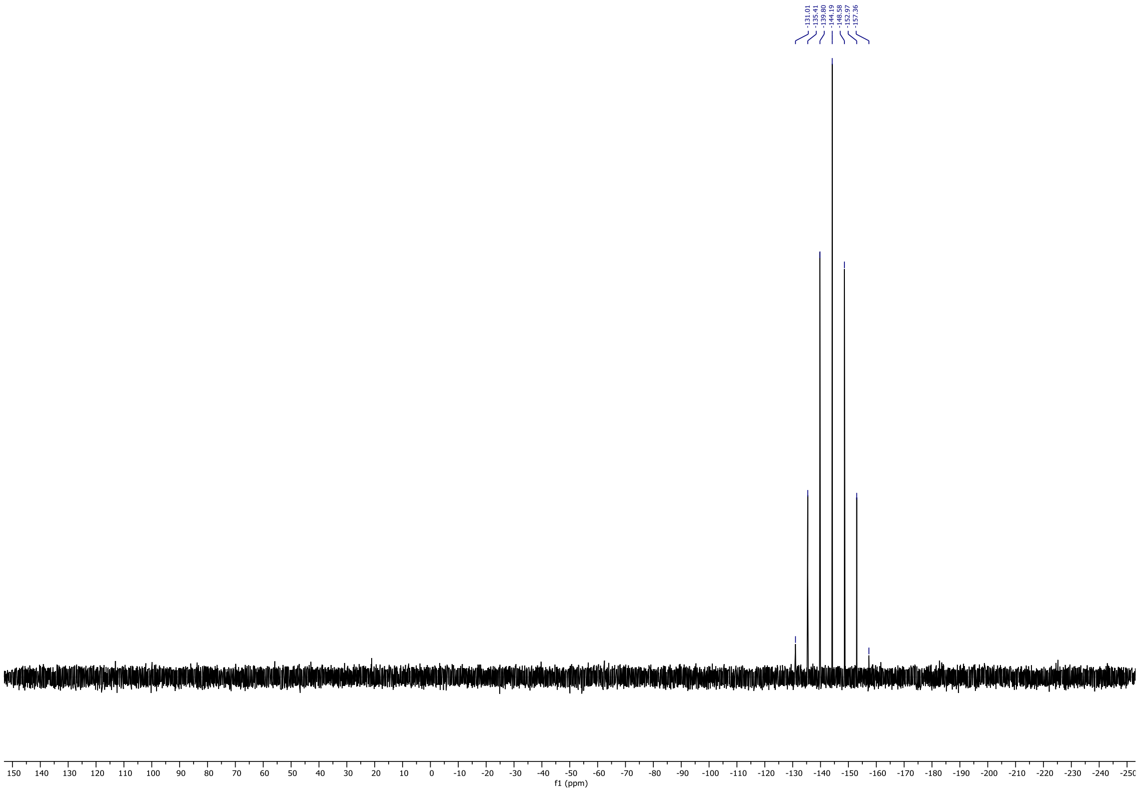

Figure S12: ^{31}P NMR Spectrum of **7a**.

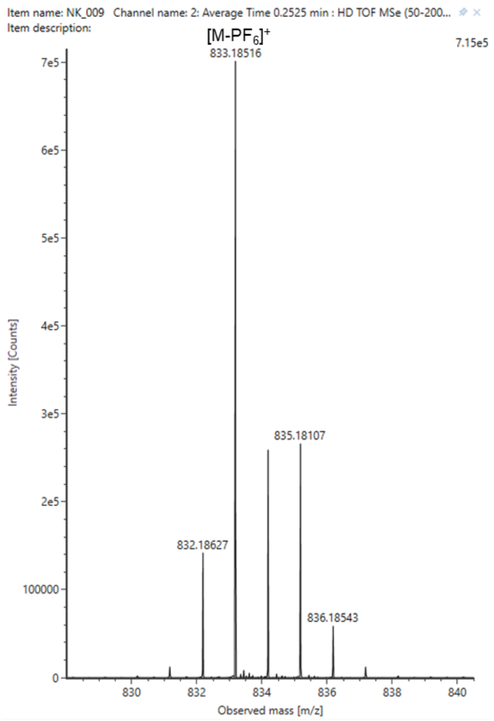

Figure S13: Zoomed-in HRMS of **7a.**

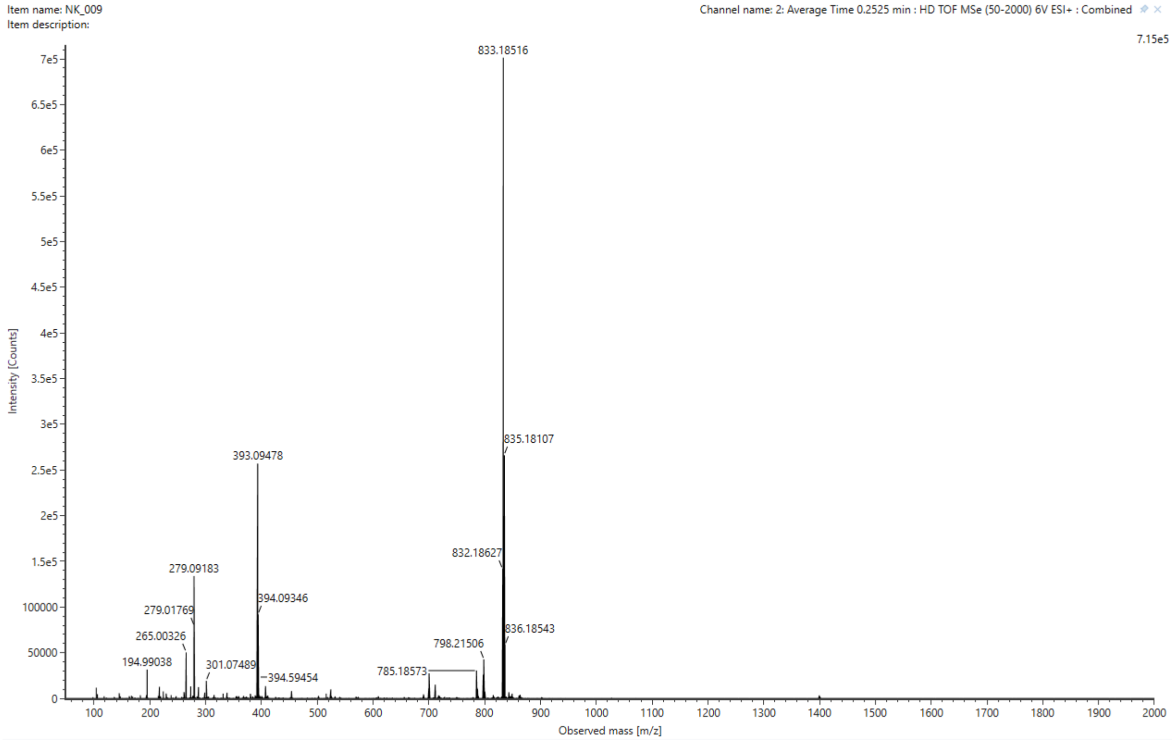

Figure S14: Full HRMS of **7a.**

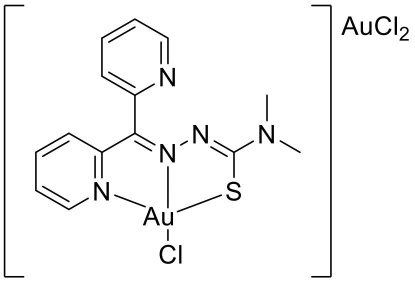

8a

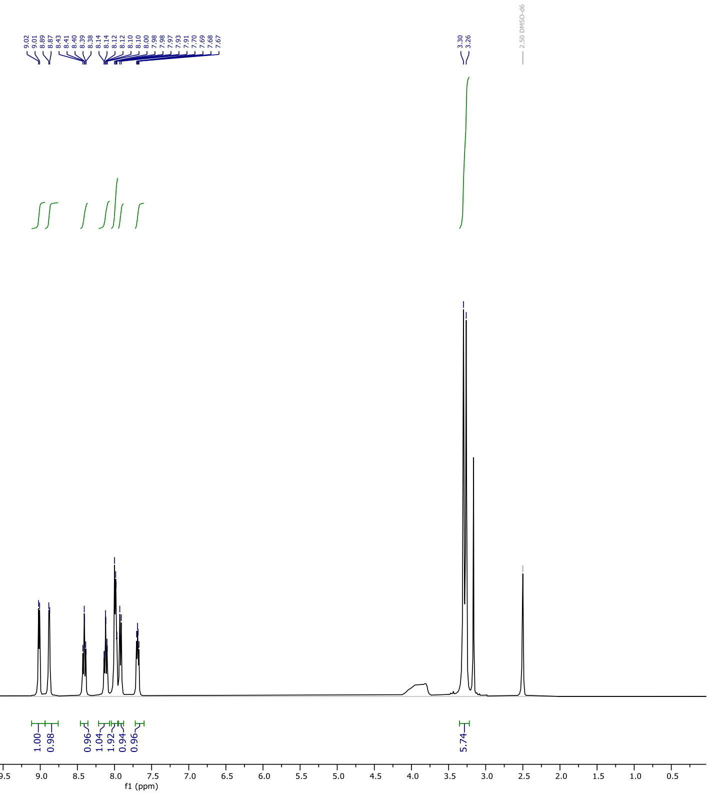

Figure S15: ^{1}H NMR Spectrum of **8a.**

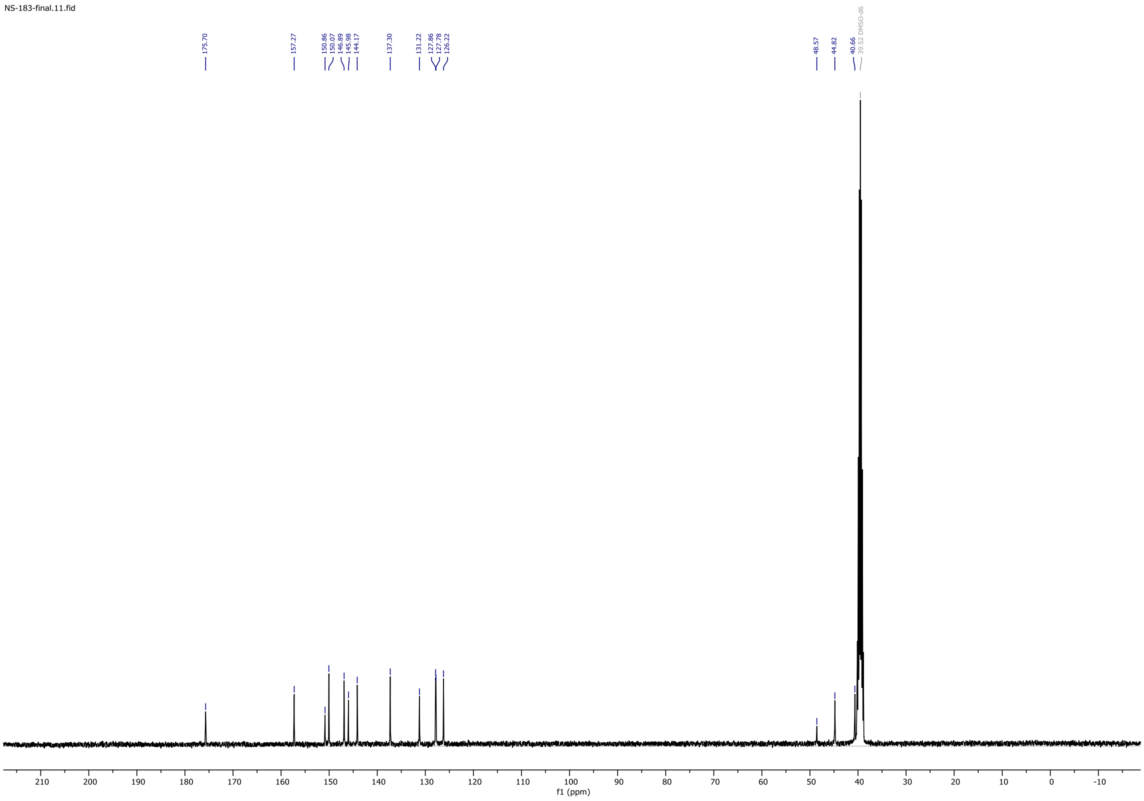

Figure S16: ^{13}C NMR Spectrum of **8a.**

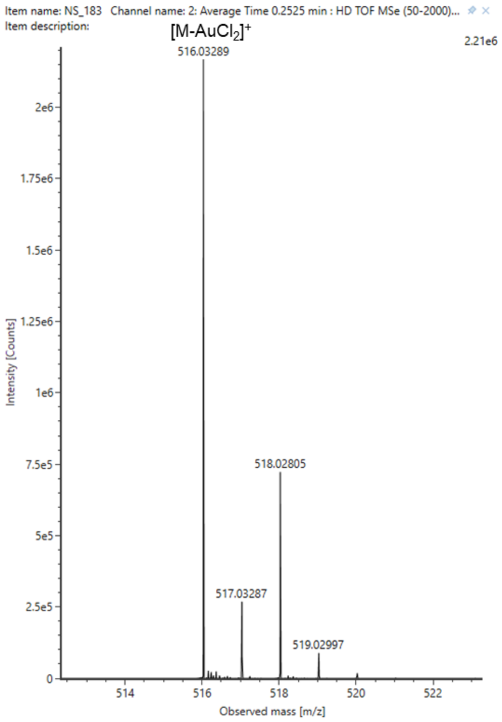

Figure S17: Zoomed-in HRMS of **8a.**

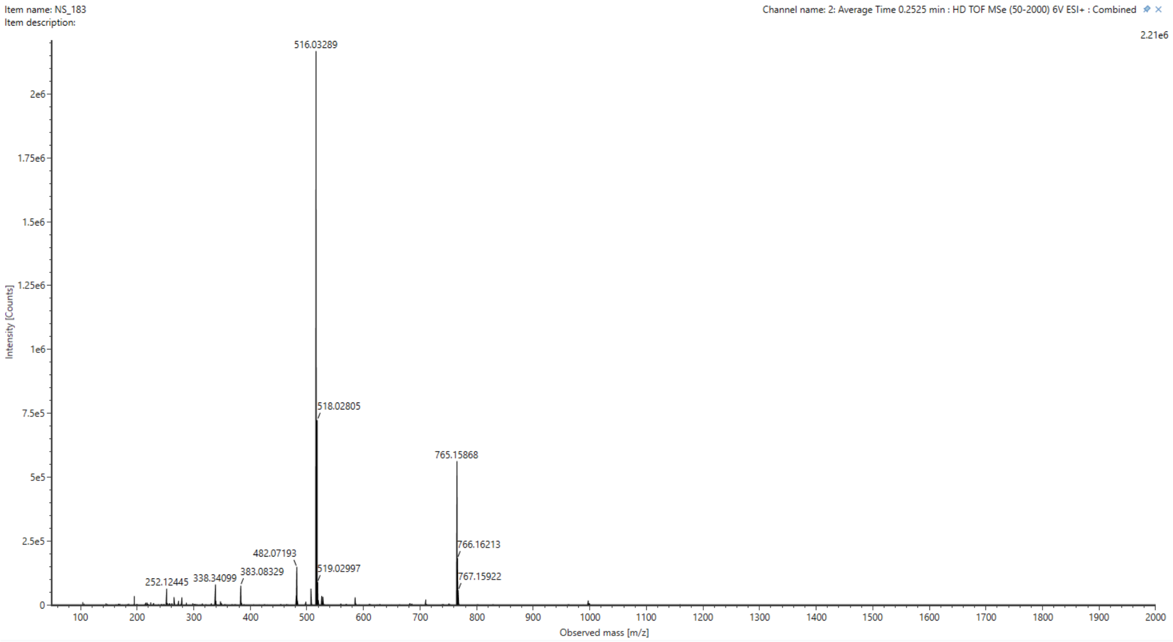

Figure S18: Full HRMS of **8a.**

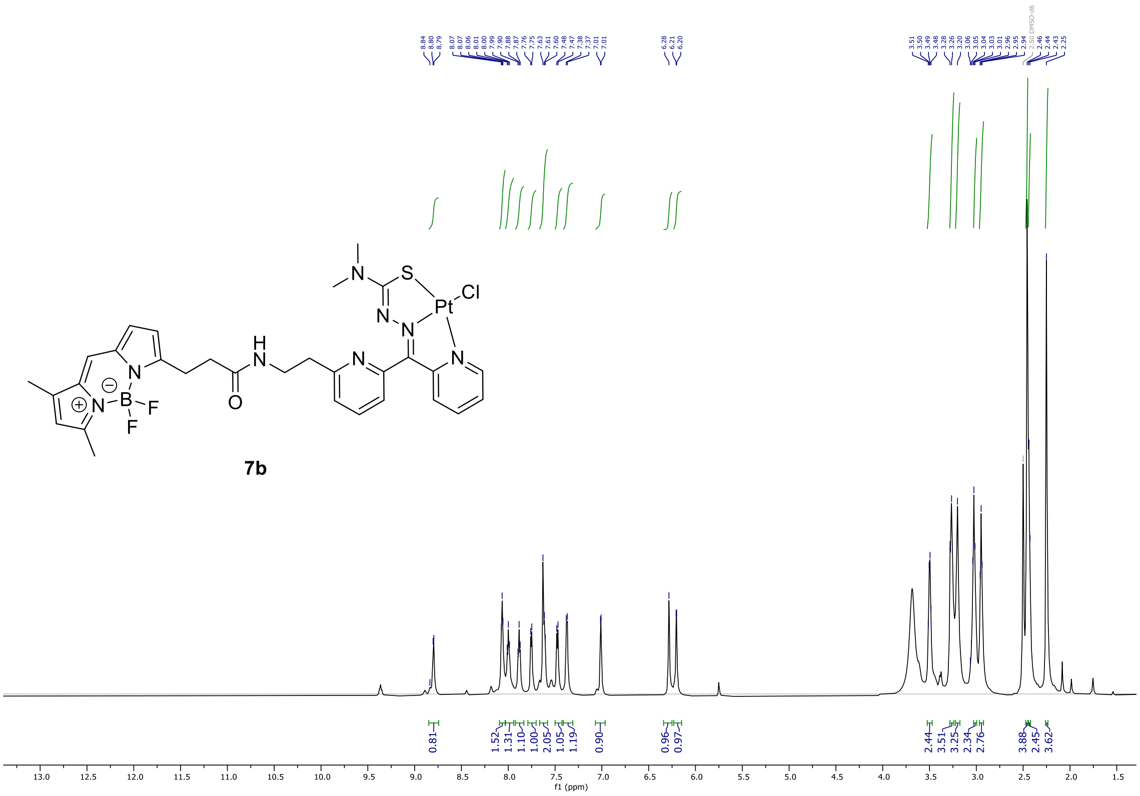

Figure S19: ^{1}H NMR Spectrum of **7b.**

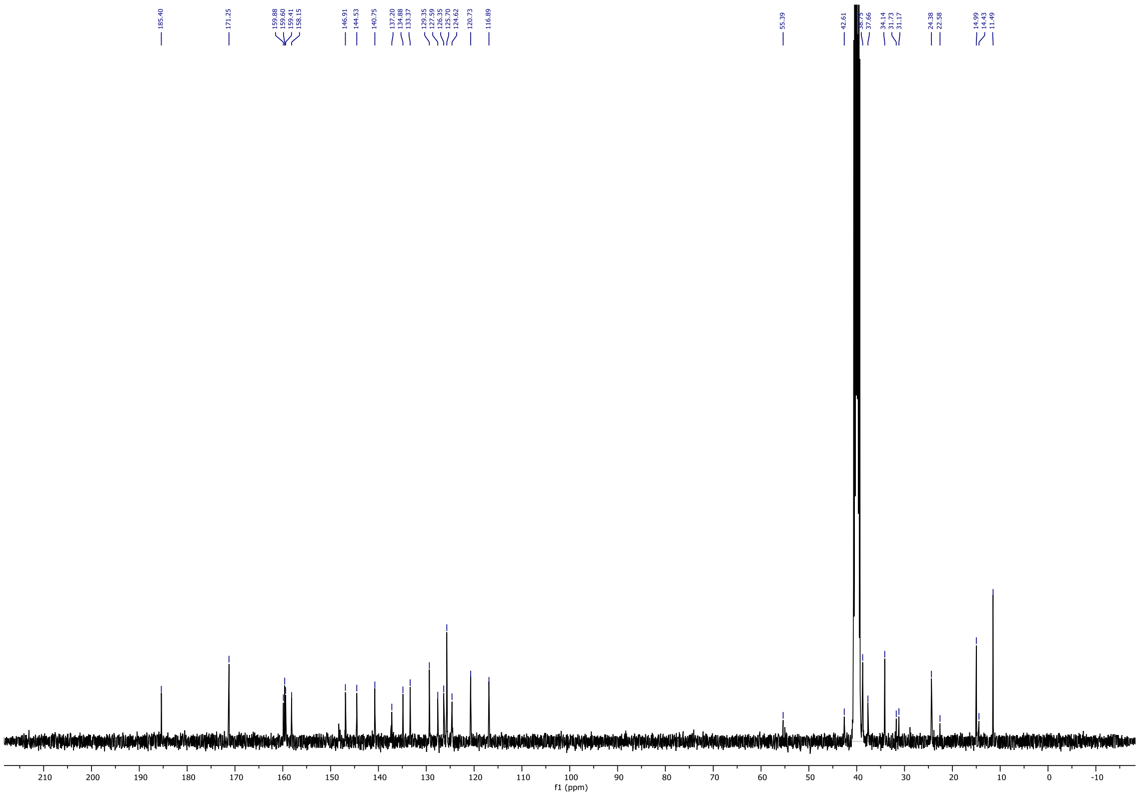

Figure S20: ^{13}C NMR Spectrum of **7b.**

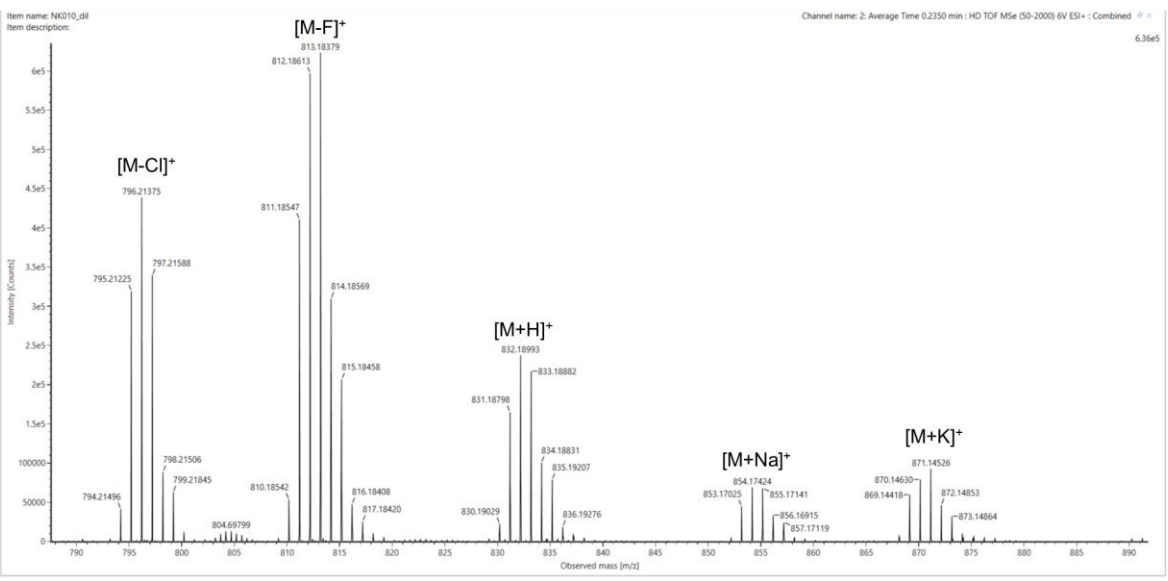

Figure S21: Zoomed-in HRMS of **7b.**

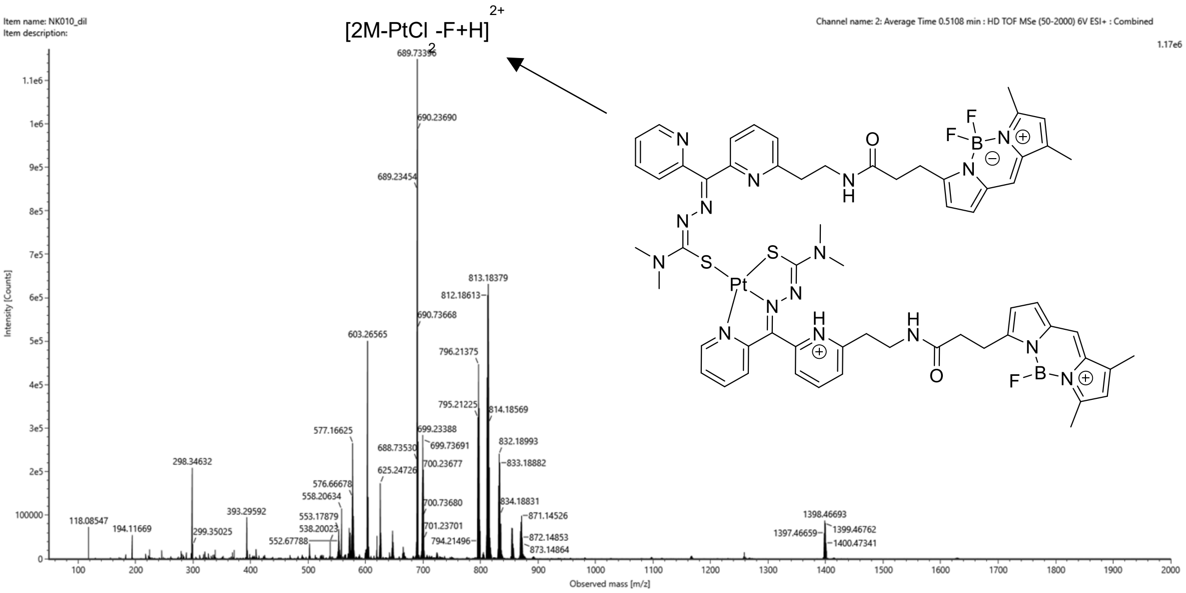

Figure S22: Full HRMS of **7b.**

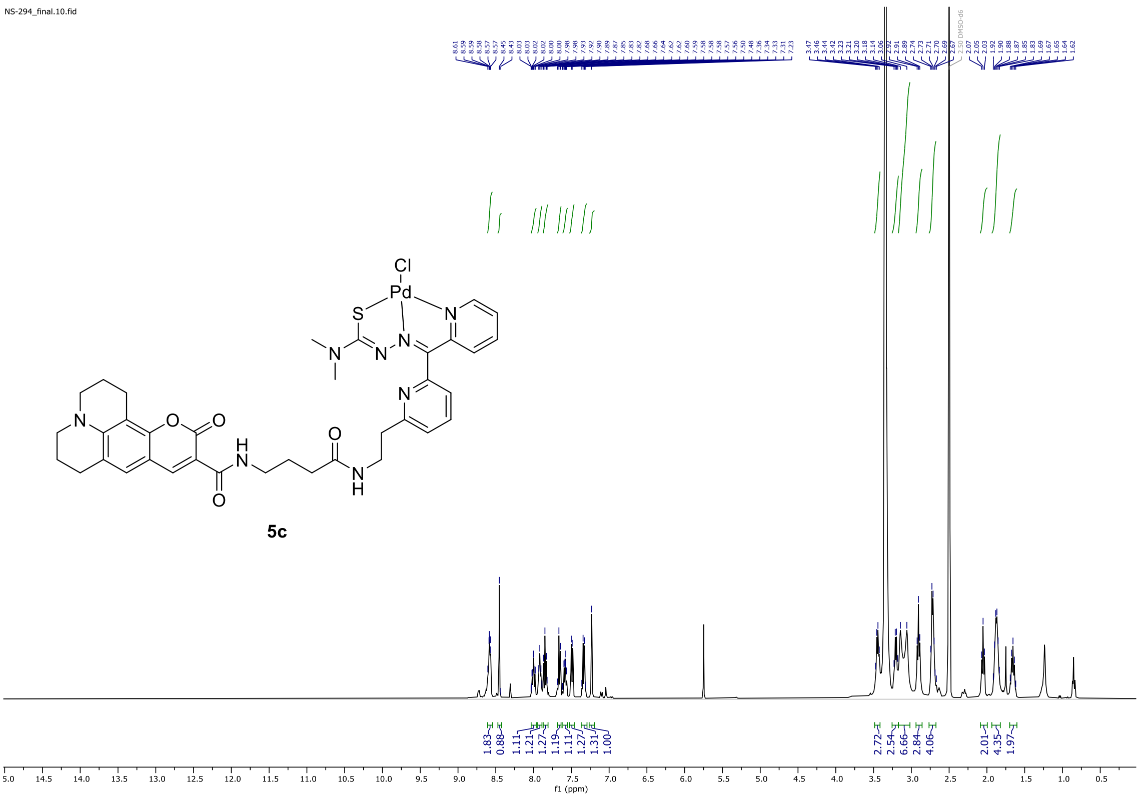

Figure S23: ^{1}H NMR Spectrum of **5c.**

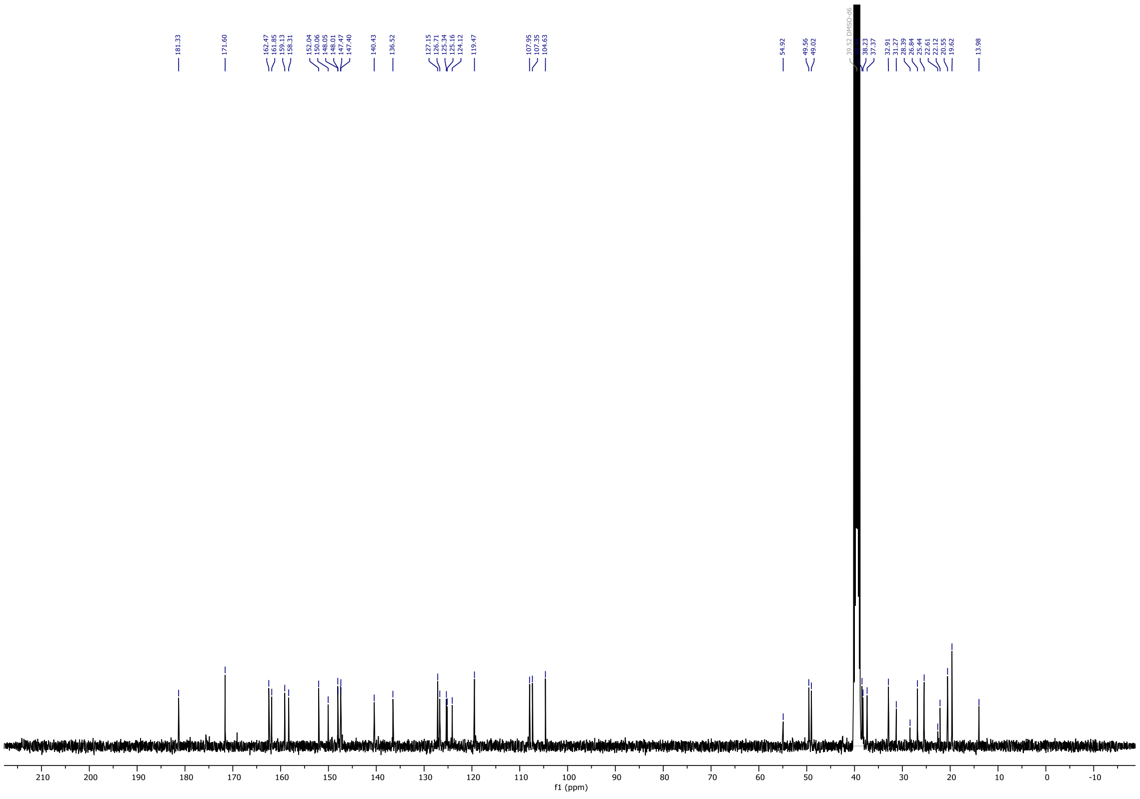

Figure S24: ^{13}C NMR Spectrum of **5c.**

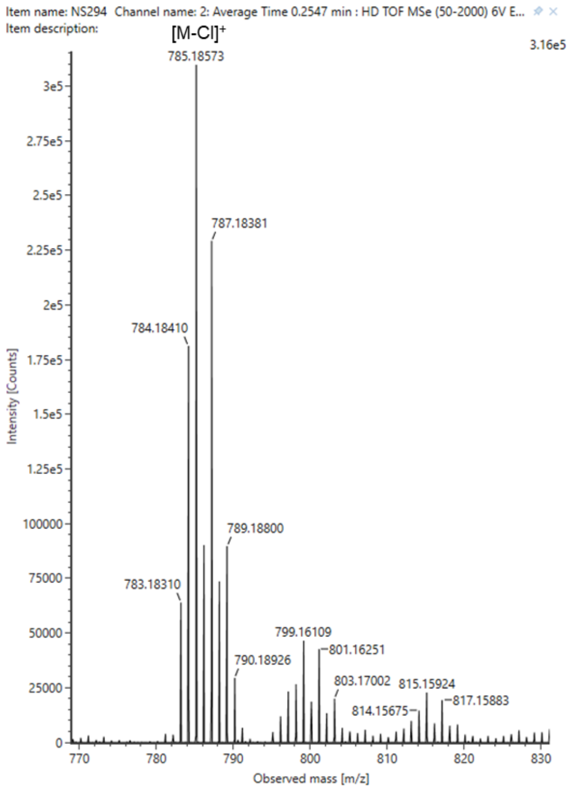

Figure S25: Zoomed-in HRMS of **5c.**

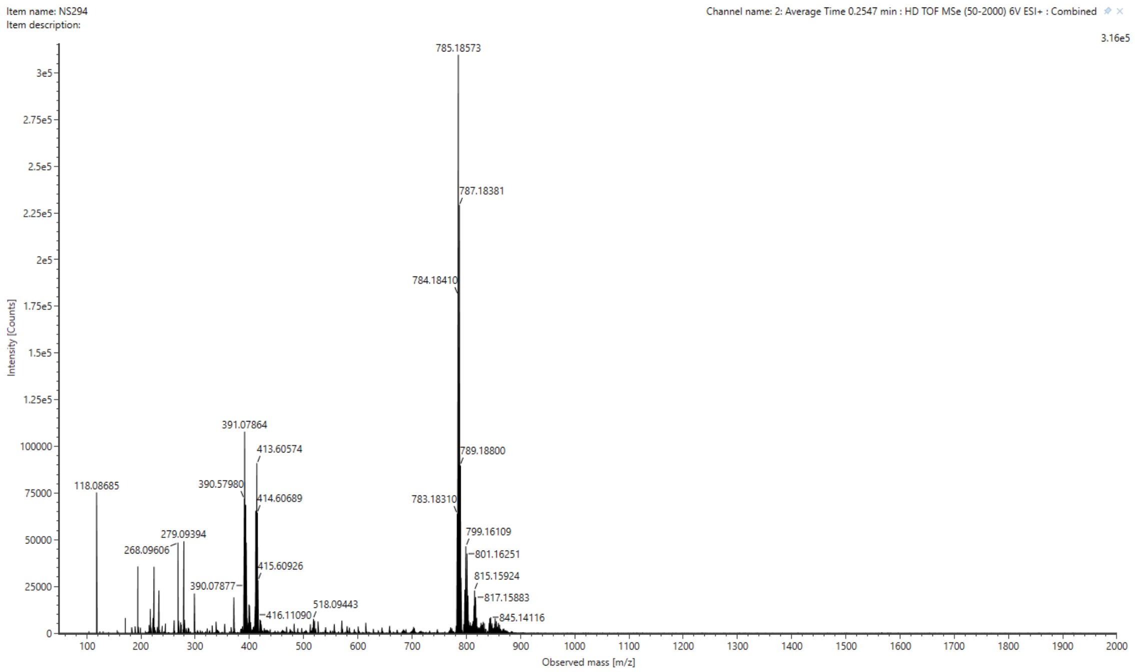

Figure S26: Full HRMS of **5c.**

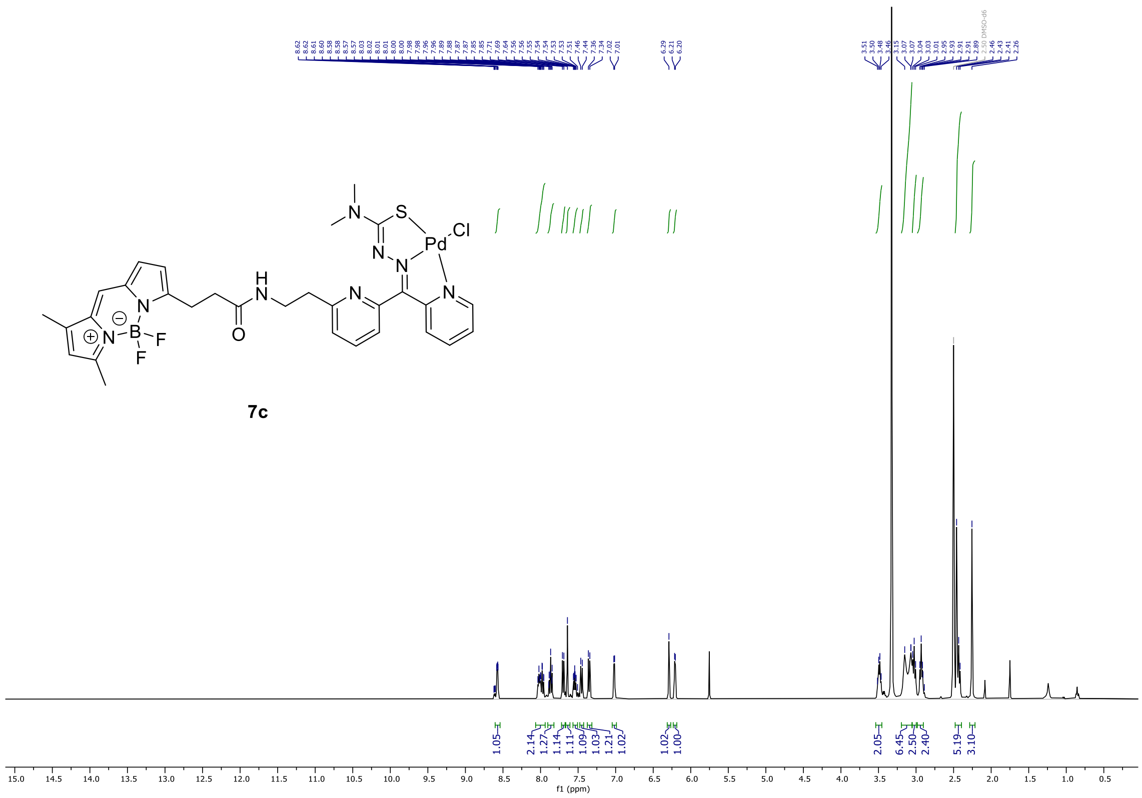

Figure S27: ^{1}H NMR Spectrum of **7c.**

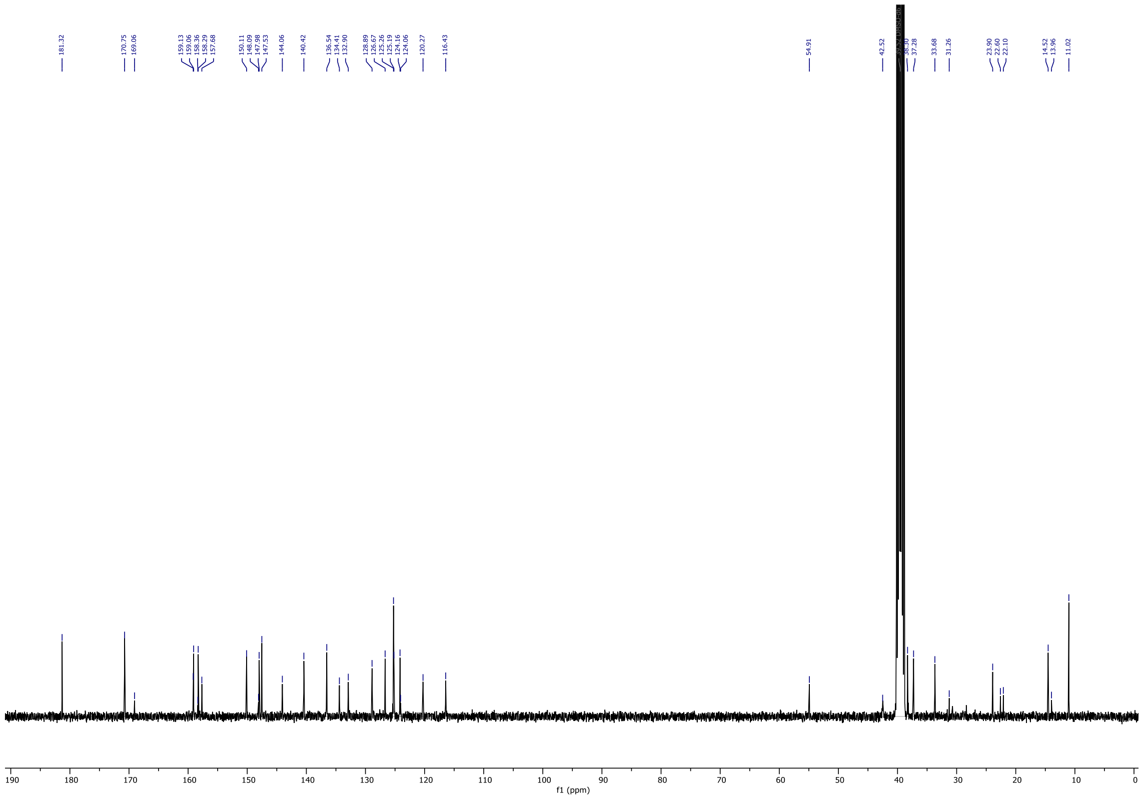

Figure S28: ^{13}C NMR Spectrum of **7c.**

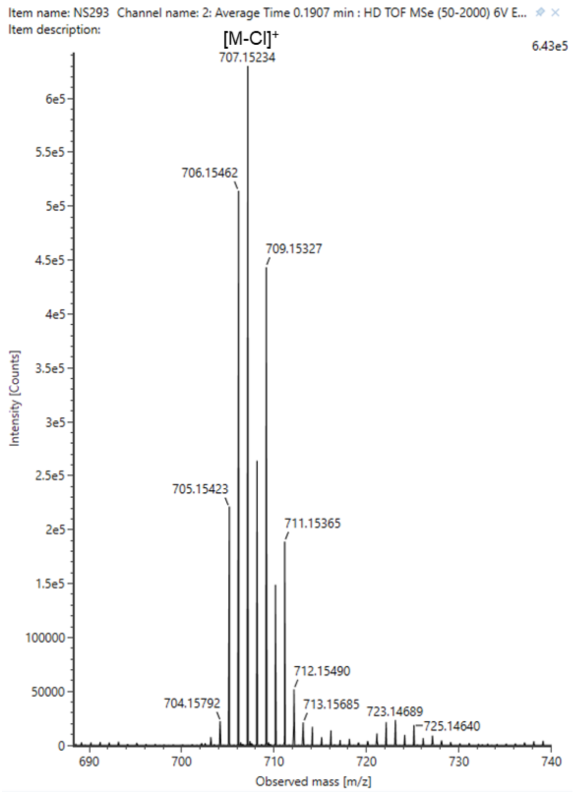

Figure S29: Zoomed-in HRMS of **7c.**

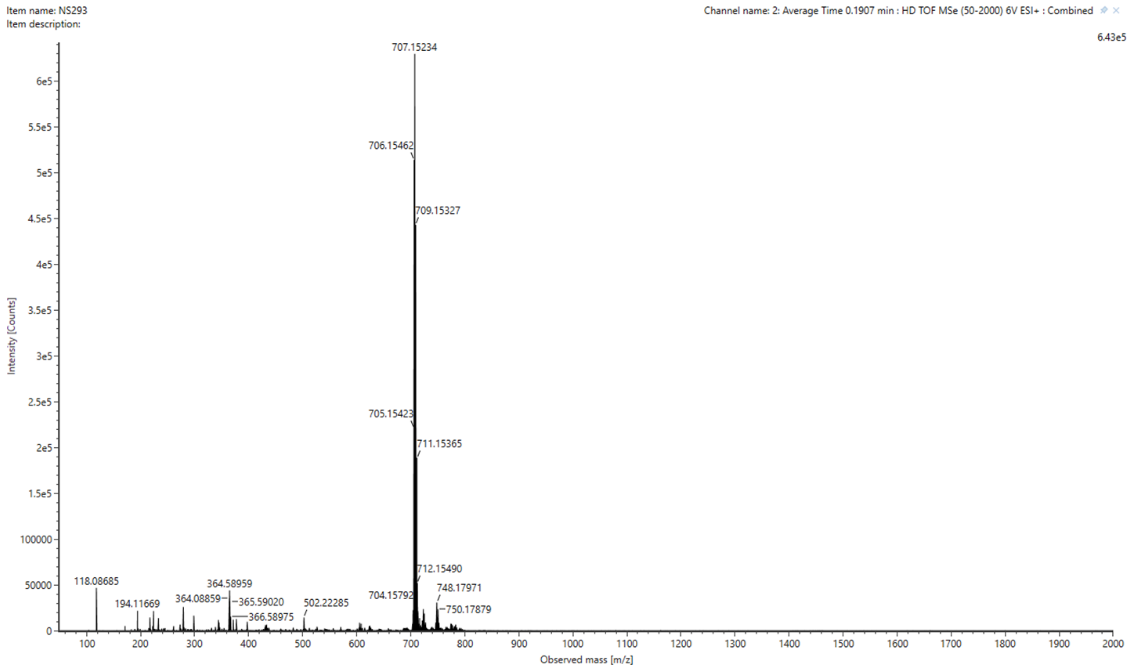

Figure S30: Full HRMS of **7c.**

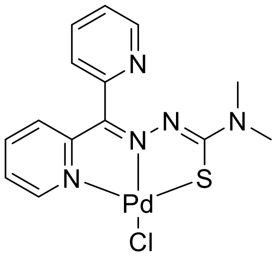

8c

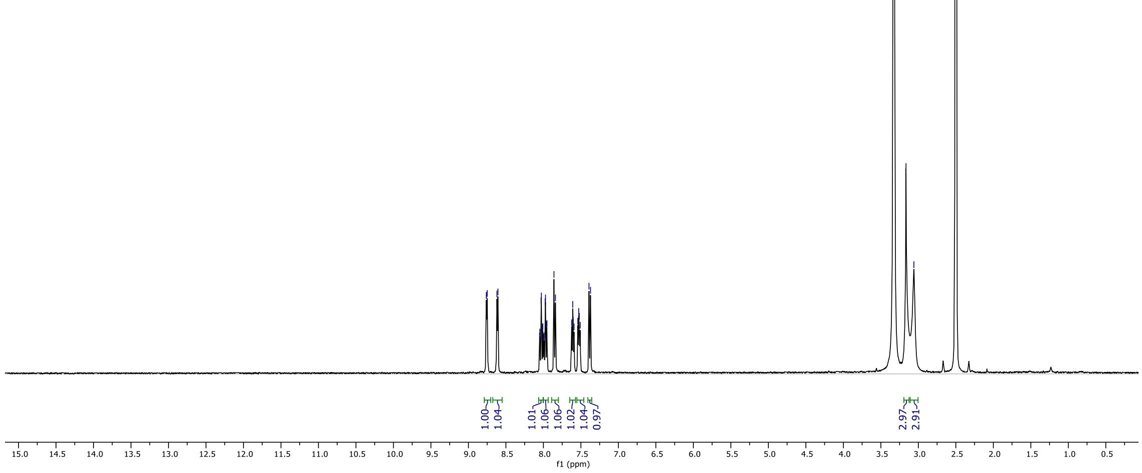

Figure S31: ^{1}H NMR Spectrum of **8c.**

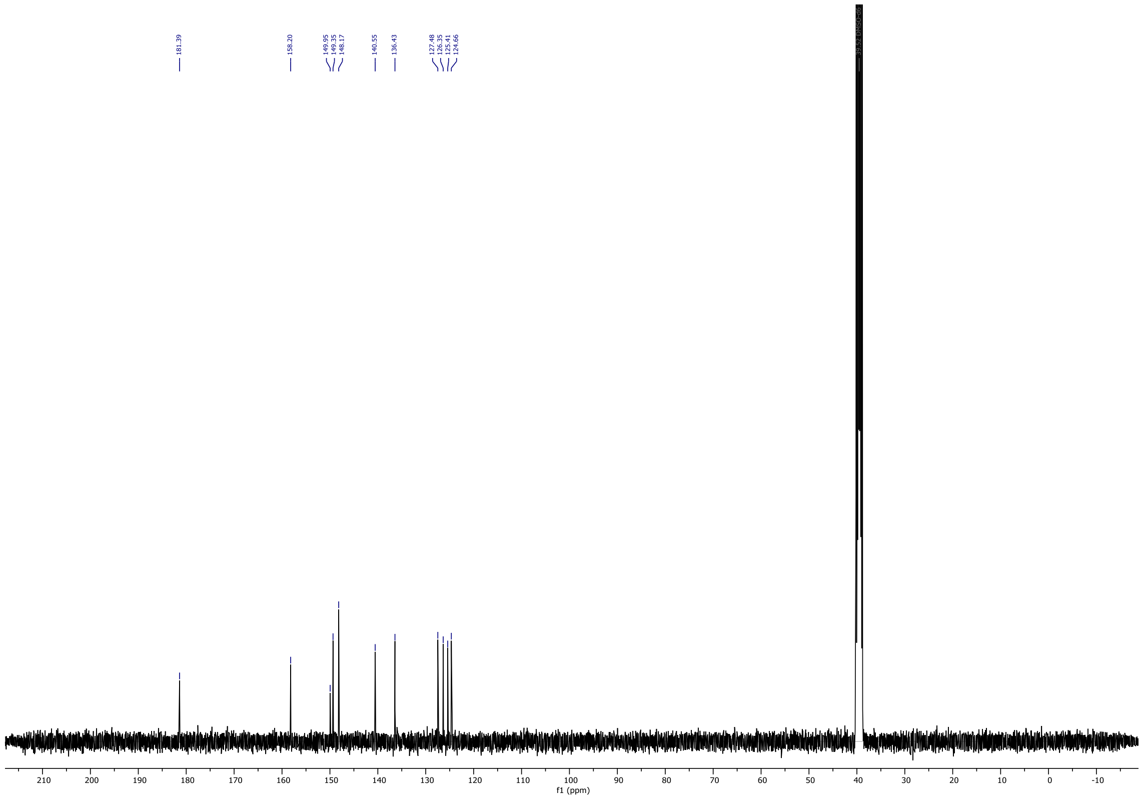

Figure S32: ^{13}C NMR Spectrum of **8c.**

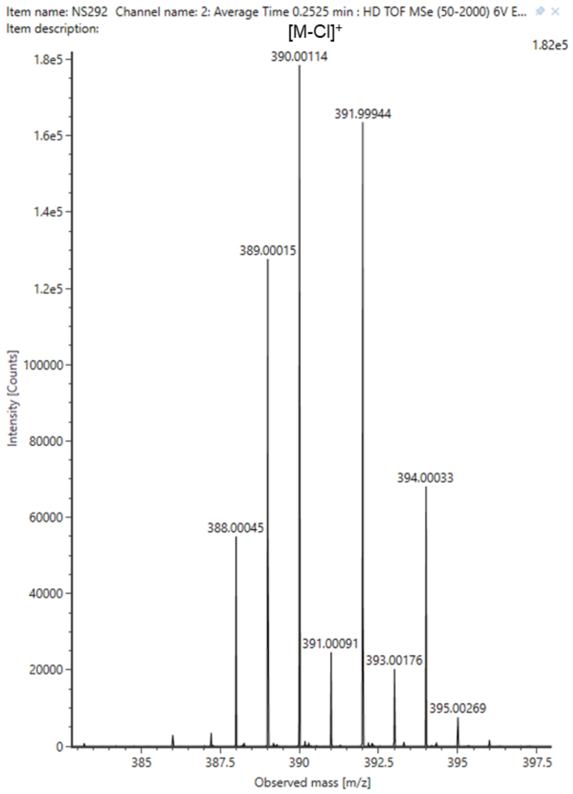

Figure S33: Zoomed-in HRMS of **8c.**

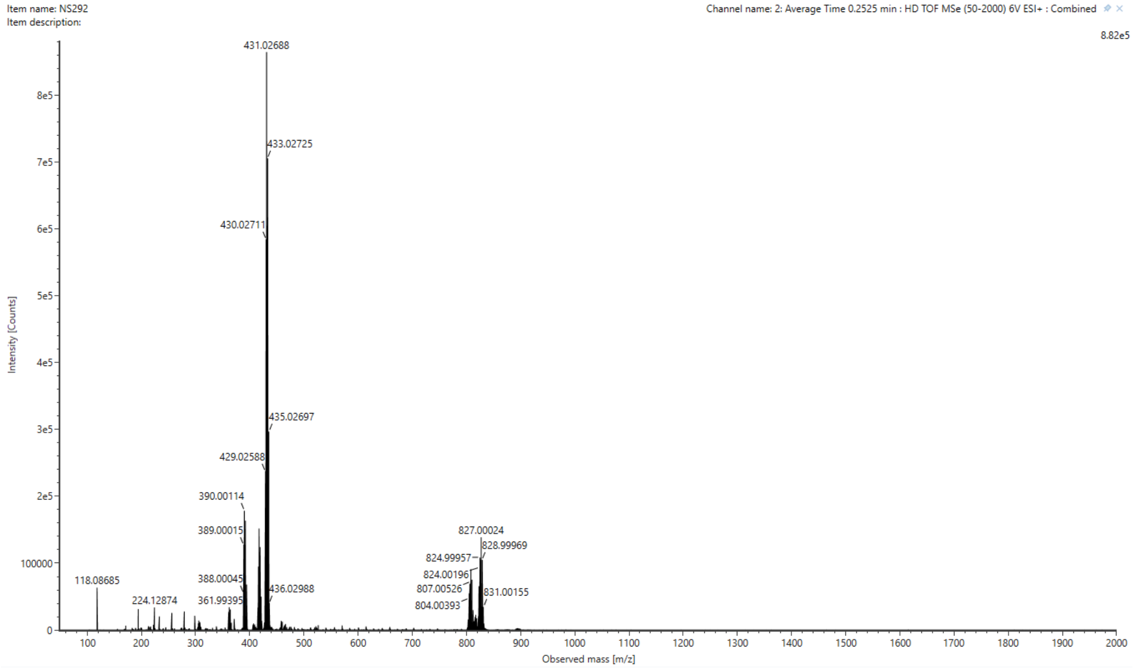

Figure S34: Full HRMS of **8c.**

5. Crystallographic data

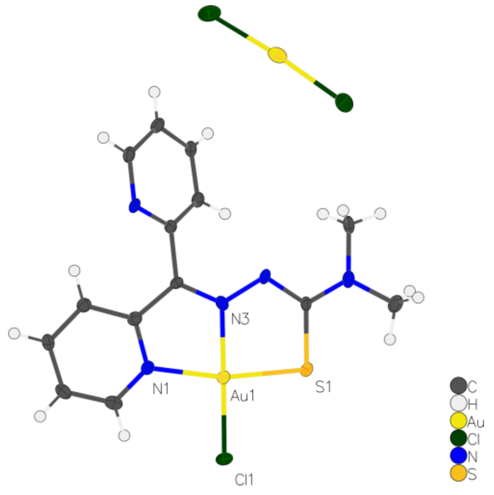

Figure S35: Structure of **8a** as determined by single crystal X-ray diffraction.

Compound	**6**
Empirical formula	$C_{14}H_{14}Au_2Cl_3N_5S$
Formula weight [g/mol]	785.658
Temperature [K]	99.98(10)
Wavelength [Å]	1.54
Crystal system	Monoclinic
Space group	$P\ 2_1/c$
Unit cell	a = 9.1509(1) α = 90<br>b = 28.7148(3) β = 91.175(1)<br>c = 7.2733(1) γ = 90
Volume [Å^3]	1910.78(4)
Z	4
Density (calculated) [Mg/m^3]	2.728
F (000)	1410.8
θ range [°]	6.16 to 153.32
Reflections collected / unique	12465
Completeness	95%
Data / restraints / parameters	3822 / 0 / 237
Goodness-of-fit on F^2	1.055
Final R indices [I>2σ(I)]	R_1 = 0.0320, wR_2 = 0.0814
R indices (all data)	R_1 = 0.0341, wR_2 = 0.0829
CCDC number	2464448

Table S1: Crystallographic data of **8a** crystallized by slow diffusion of hexane into acetonitrile.

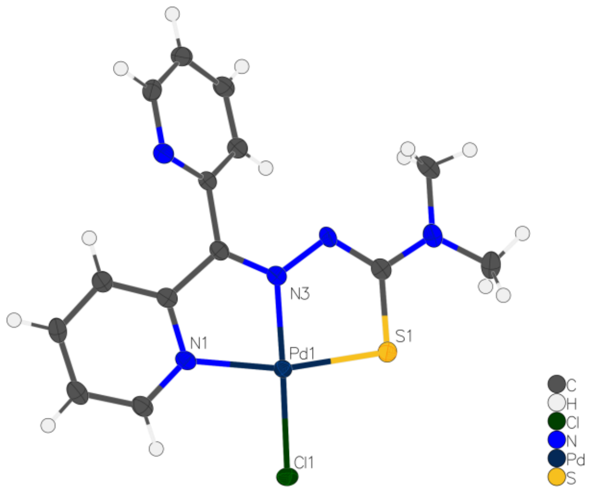

Figure S36: Structure of **8c** as determined by single crystal X-ray diffraction.

Compound	**8c**
Empirical formula	$C_{14}H_{14}ClN_5PdS$
Formula weight [g/mol]	426.21
Temperature [K]	100.00(3)
Wavelength [Å]	1.54
Crystal system	Monoclinic
Space group	P 2_1/n
Unit cell	a = 11.3065(10) α = 90<br>b = 8.71280(10) β = 101.8980(10)<br>c = 16.0160(2) γ = 90
Volume [Å^3]	1543.86(3)
Z	4
Density (calculated) [Mg/m^3]	1.834
F (000)	848.0
θ range [°]	8.78 to 153.19
Reflections collected / unique	10242
Completeness	95%
Data / restraints / parameters	3079 / 0 / 201
Goodness-of-fit on F^2	1.056
Final R indices [I>2σ(I)]	R_1 = 0.0308, wR_2 = 0.0841
R indices (all data)	R_1 = 0.0322, wR_2 = 0.0855
CCDC number	2464449

Table S2: Crystallographic data of **8c** crystallized by slow evaporation of methanol

6. Stability Studies

DMSO Stability

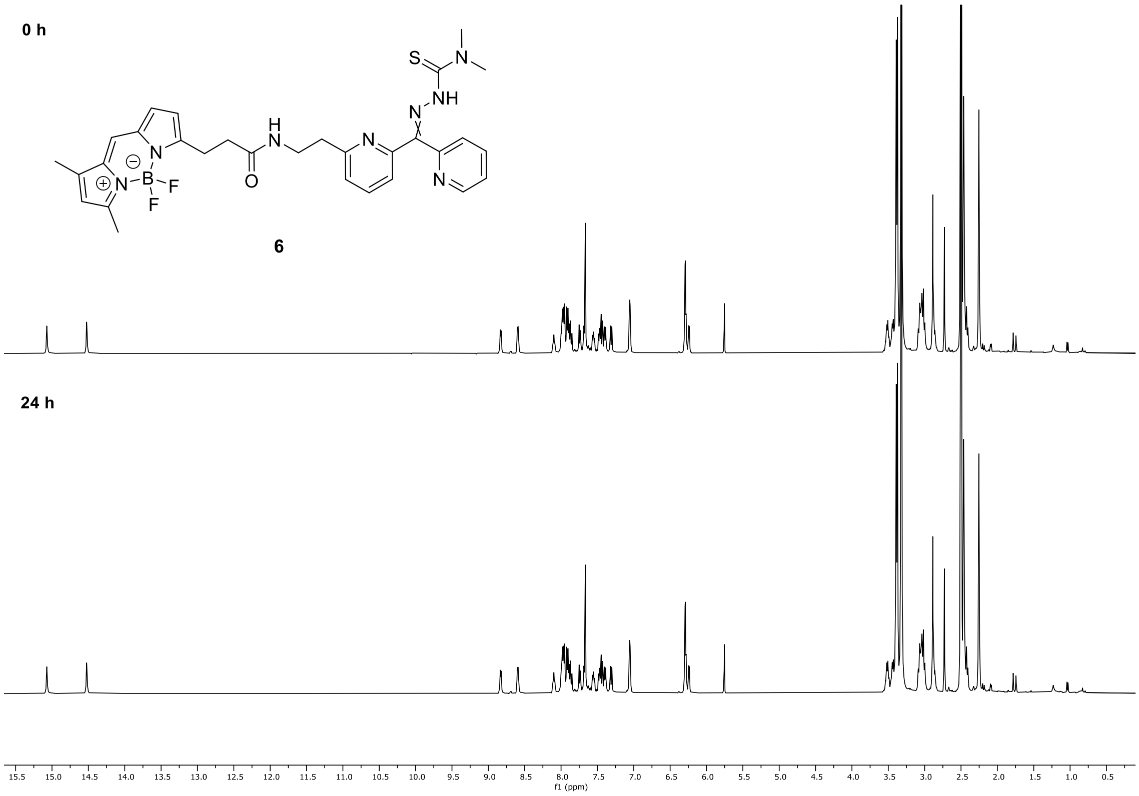

Figure S37: Stability studies of **6** in DMSO-d_6 over 24 h using ^{1}H NMR spectroscopy

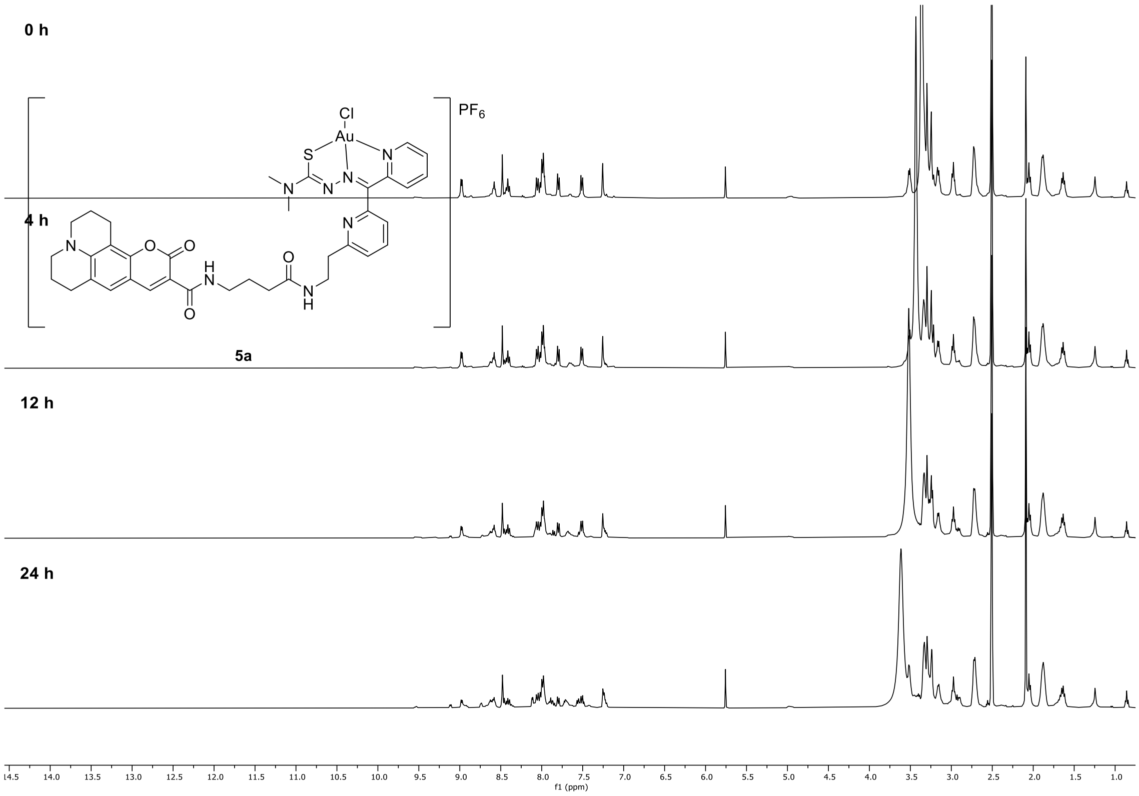

Figure S38: Stability studies of **5a** in DMSO-d_6 over 24 h using ^{1}H NMR spectroscopy

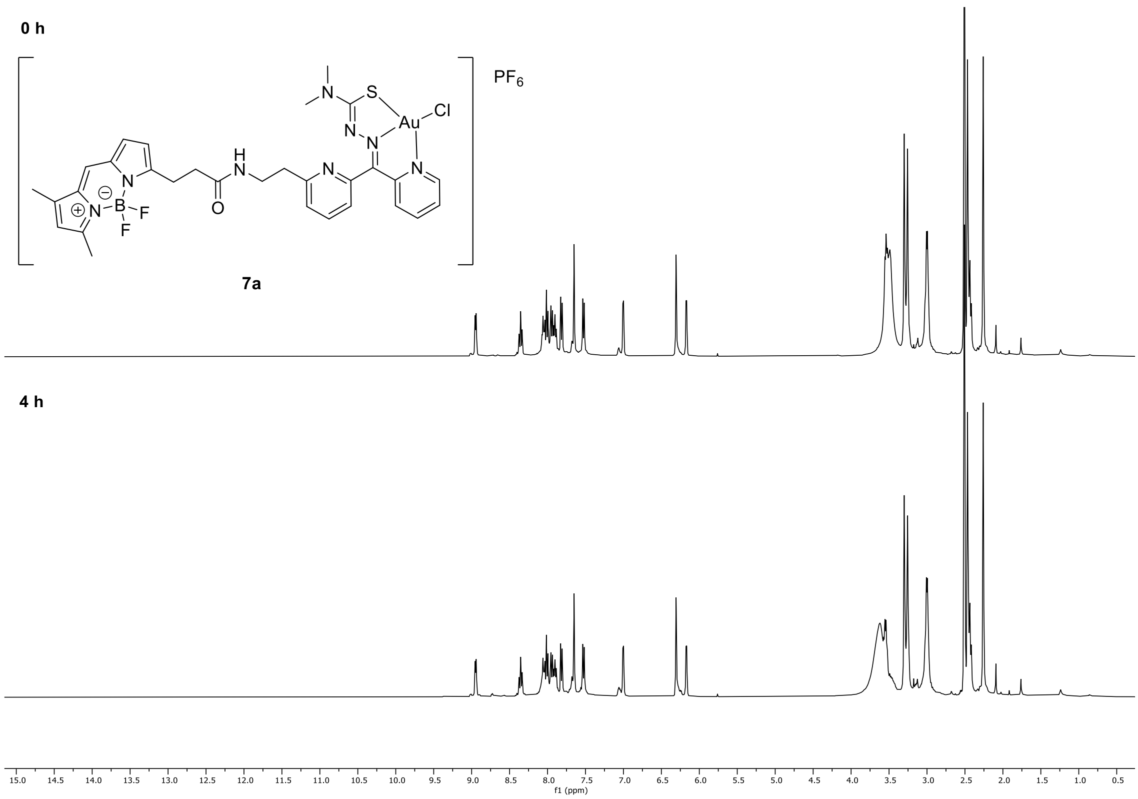

Figure S39: Stability studies of **7a** in DMSO-d$_6$ over 24 h using ^{1}H NMR spectroscopy

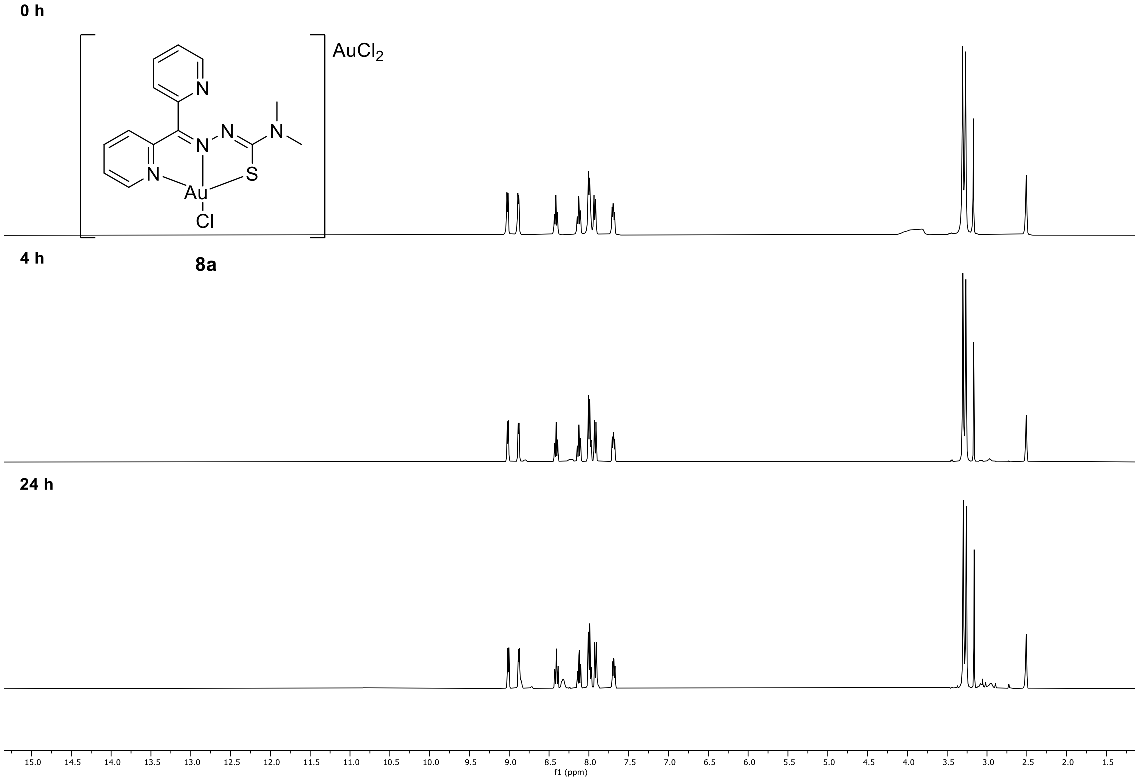

Figure S40: Stability studies of **8a** in DMSO-d$_6$ over 24 h using ^{1}H NMR spectroscopy

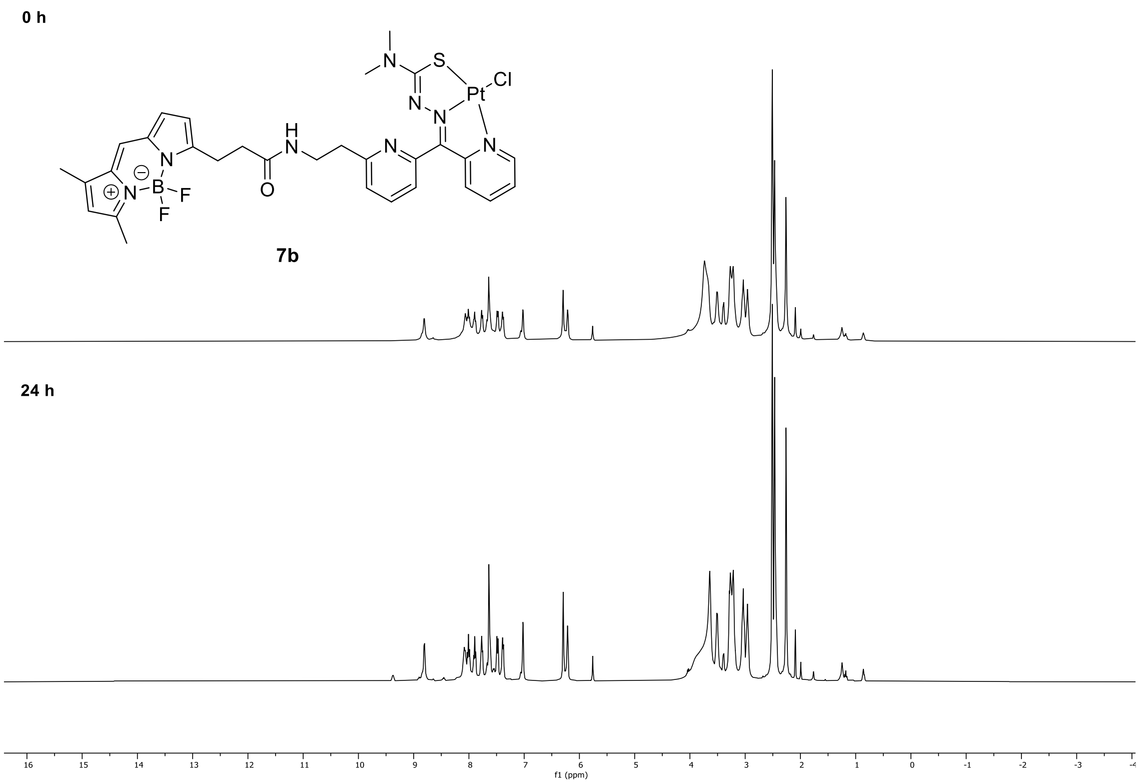

Figure S41: Stability studies of **7b** in DMSO-d_6 over 24 h using ^1H NMR spectroscopy

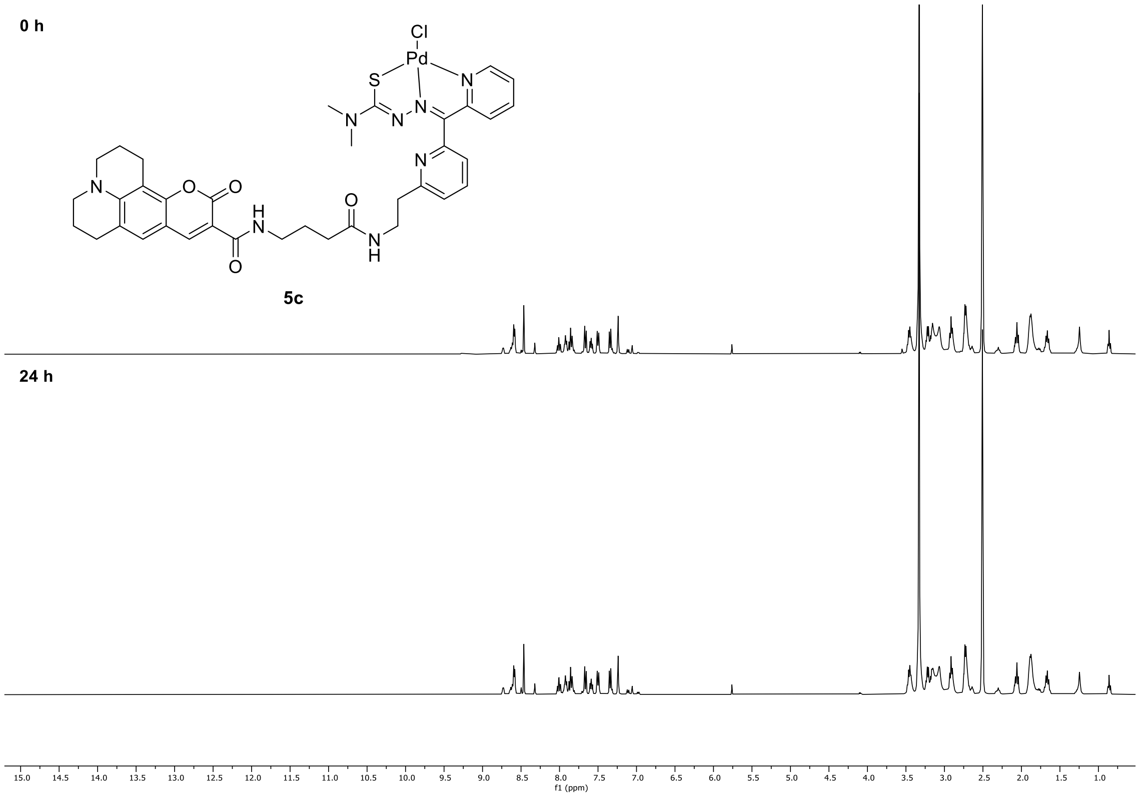

Figure S42: Stability studies of **5c** in DMSO-d_6 over 24 h using ^1H NMR spectroscopy

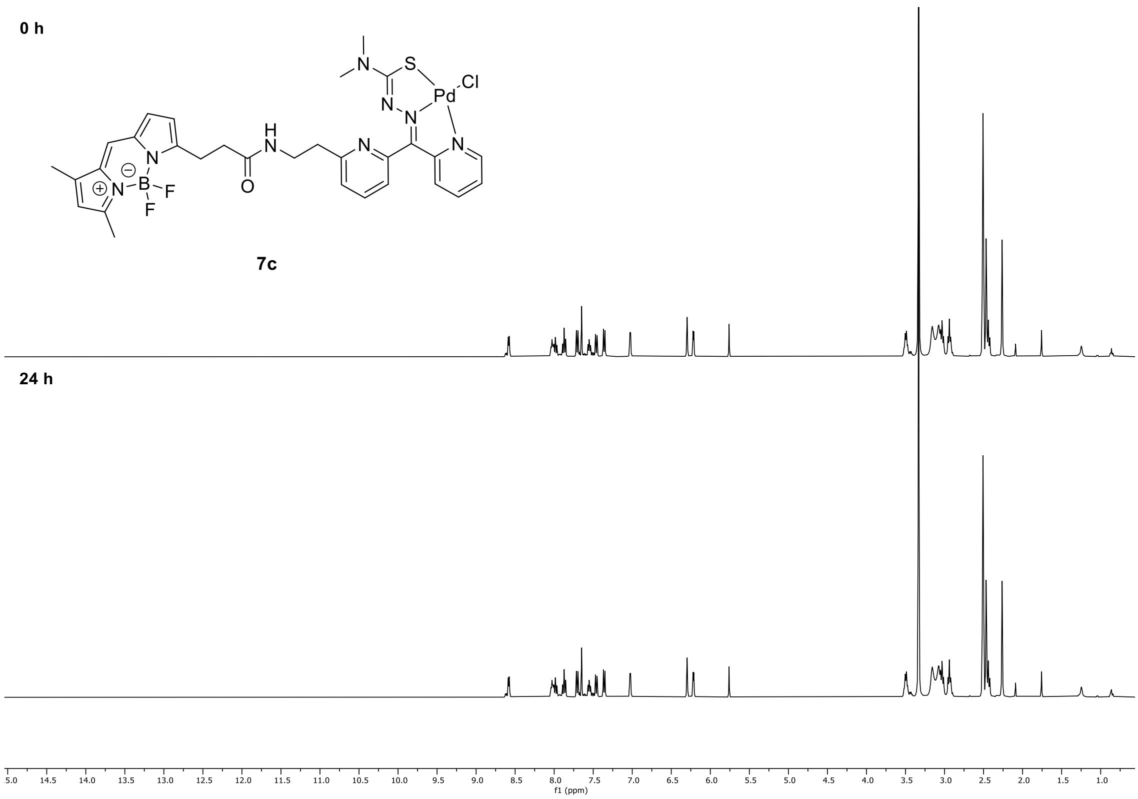

Figure S43: Stability studies of **7c** in DMSO-d_6 over 24 h using ^{1}H NMR spectroscopy

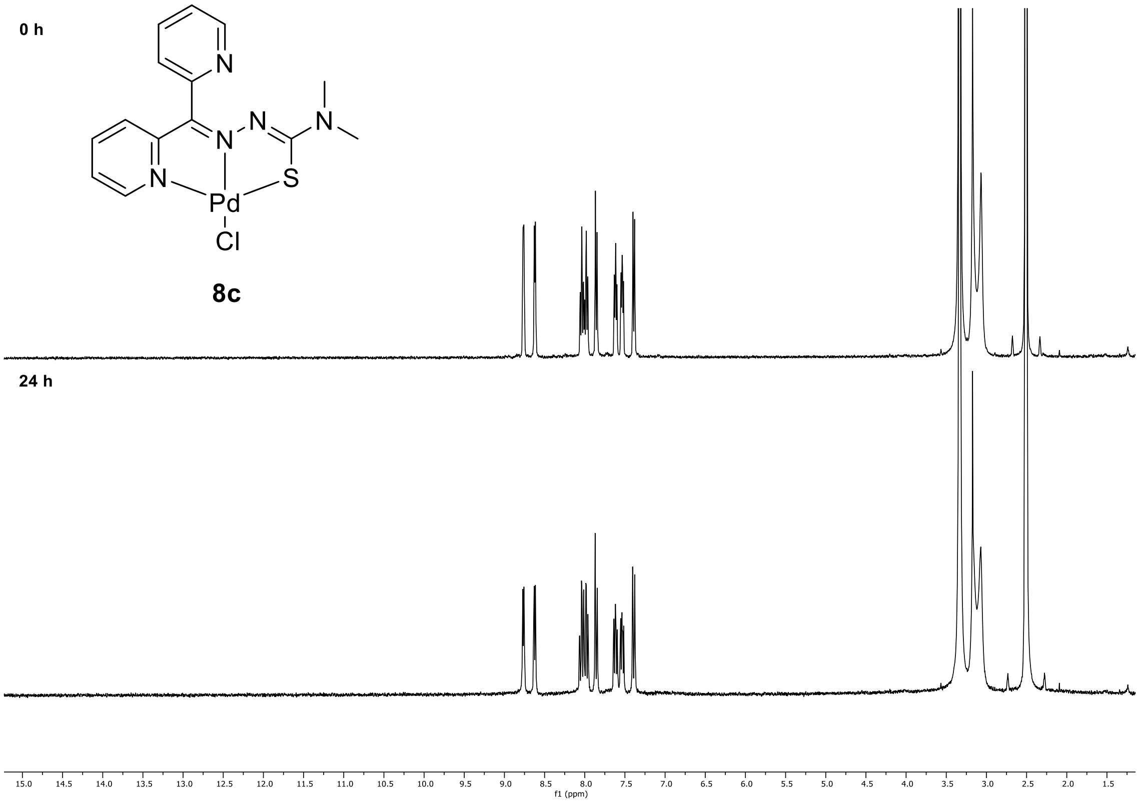

Figure S44: Stability studies of **8c** in DMSO-d_6 over 24 h using ^{1}H NMR spectroscopy

Stability in Cell Culture Medium and Water

5 mM stocks of the respective compounds in DMSO were diluted to produce a final concentration of 50 µM in water or Dulbecco's Modified Eagle's Medium (DMEM) containing 10% fetal bovine serum and 1% penicillin + streptomycin, respectively. The stability of the compounds was monitored via UV/Vis spectroscopy by recording the corresponding absorption profiles of the 50 µM solutions and comparing any changes to the profiles over time.

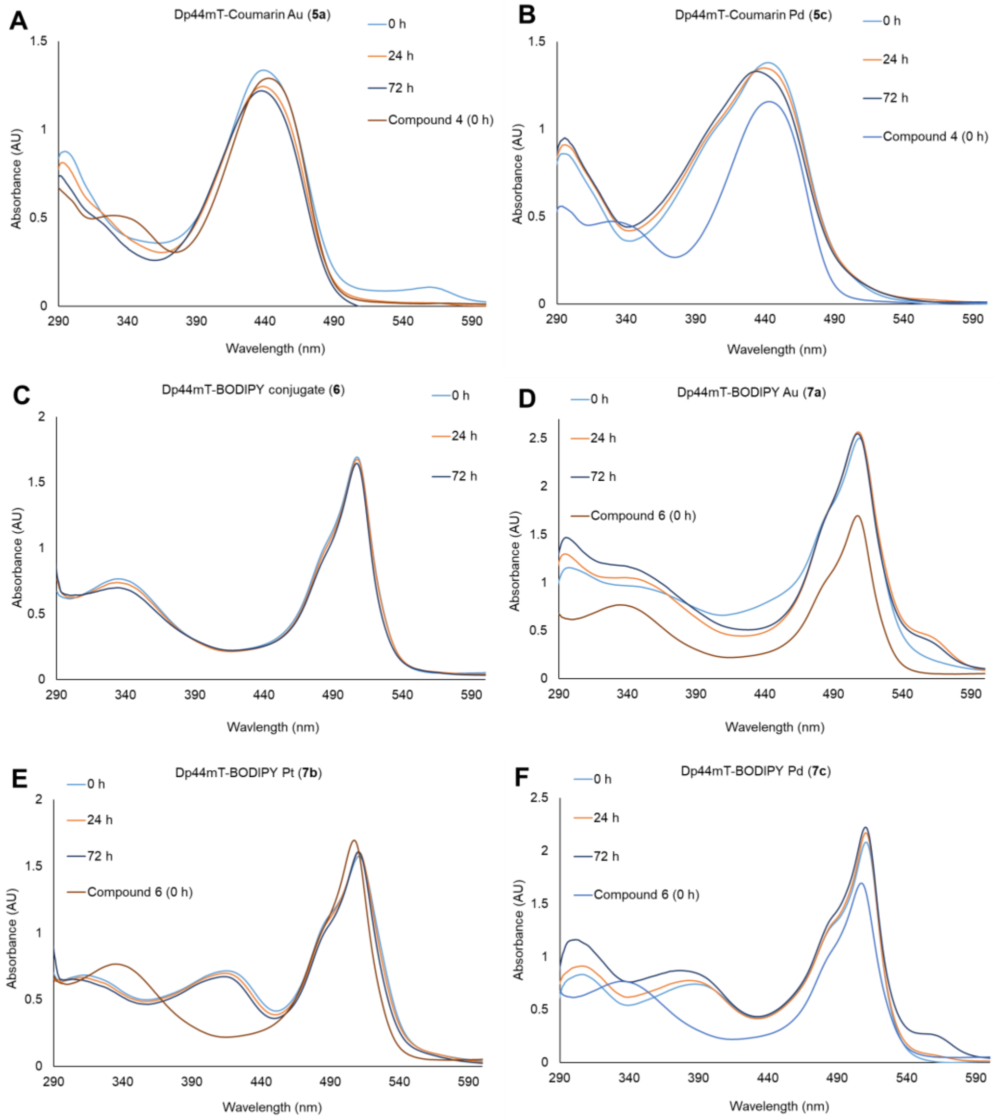

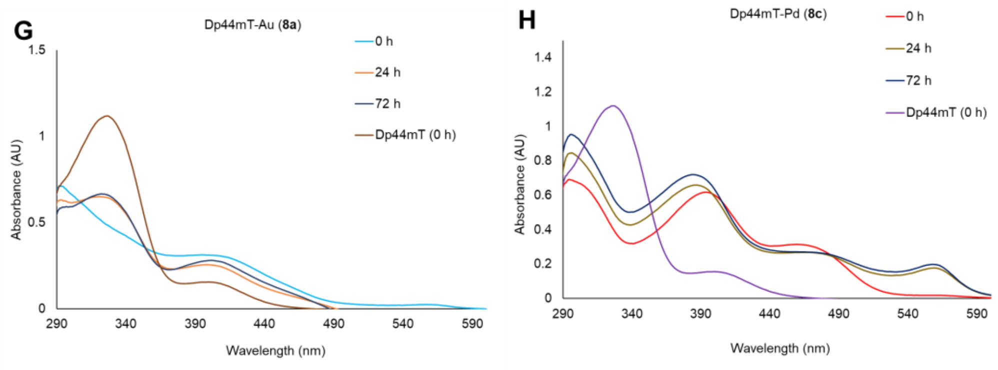

Figure S45: Stability studies of compounds in cell culture media over 72 h monitored using UV/Vis spectroscopy.

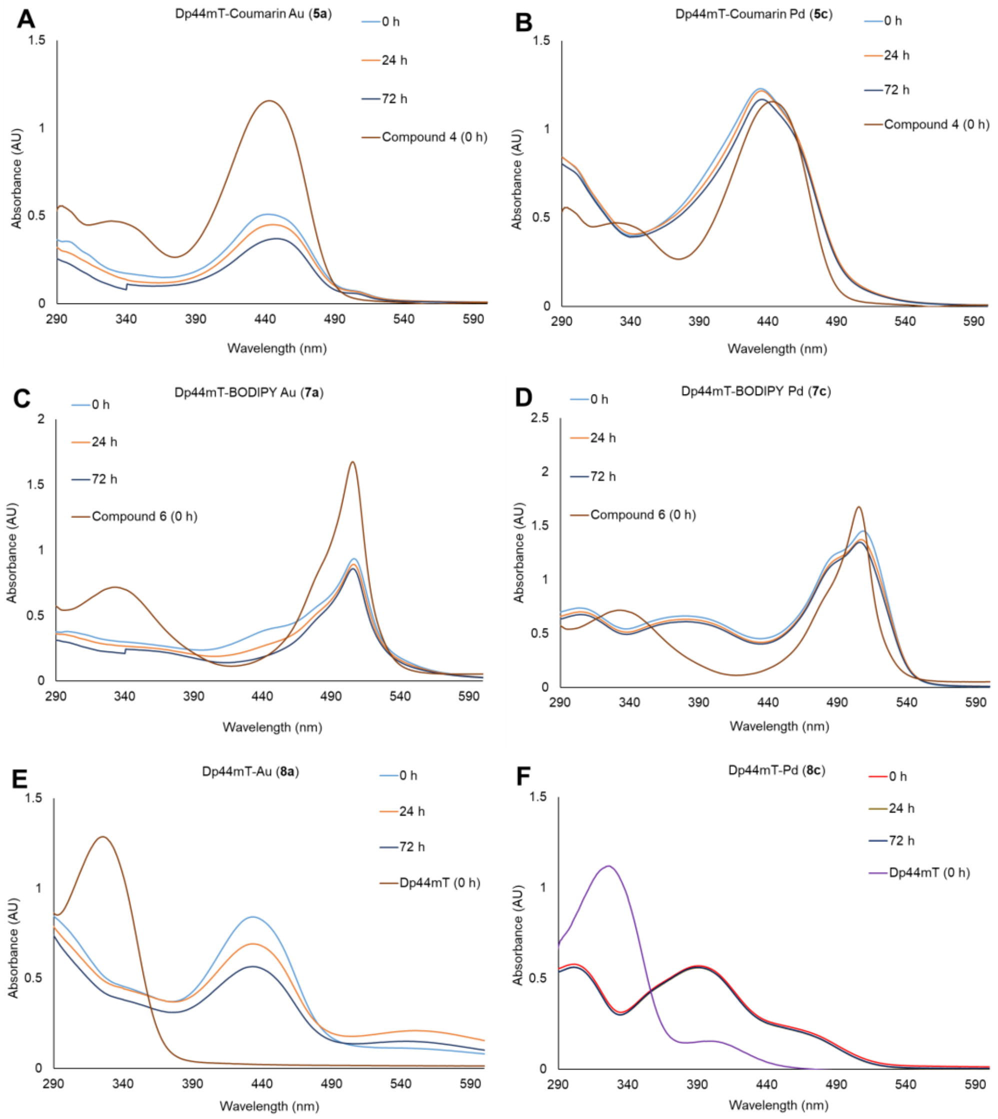

Figure S46: Stability studies of compounds in water over 72 h monitored using UV/Vis spectroscopy.

Stability at pH 5 in the presence of Cu(II)

5 mM stocks of the respective compounds in DMSO were diluted to reach a final concentration of 50 µM in acetate buffer (100 mM, pH = 5). 5 eq. of $CuCl_2$ was added wherever Cu(II) addition is indicated.

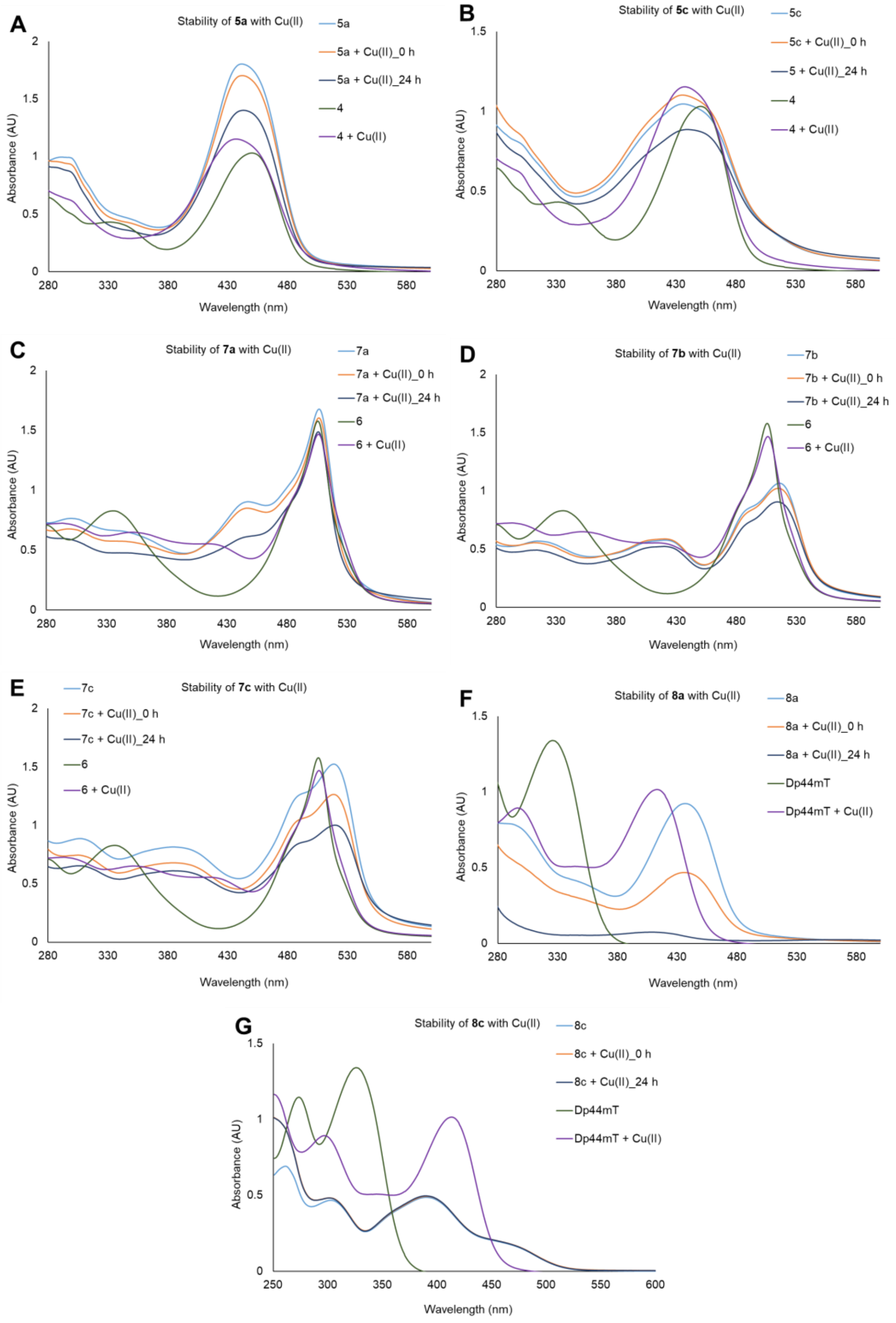

Figure S47: Stability studies of compounds in 100 mM acetate buffer at pH 5 in the presence of 5 eq. $CuCl_2$ over 24 h monitored using UV/Vis spectroscopy. Absorption profiles of the corresponding ligands have been included as references.

7. Lipophilicity (log$D_{7.2}$)

500 mL aliquots of purified octanol and PBS were thoroughly mixed in a 2 L separatory funnel and left overnight to saturate the two phases. For compound sets **5** and **7**, 20 µM standard solutions were prepared in PBS-saturated octanol, respectively. 5 mL standard solutions containing the compound in question were then pipetted into 15 mL centrifuge tubes, and a 5 mL aliquot of the octanol-saturated PBS was added. The resulting samples were shaken for 10 min and allowed to rest overnight for 18 hours. The samples were then centrifuged for 30 min (3000 g, 20 °C), and aliquots of the octanol phase were analyzed via UV-Vis spectroscopy. Experiments were conducted in triplicate. The concentrations of the compounds in the octanol phase were then quantified with respect to their $\lambda_{max.}$

log*D* was defined as:

$$logD = log\left(\frac{A_0}{A_0 - A_s}\right)$$

where A_0 = absorbance of 20 µM compound in PBS-saturated octanol

A_s= absorbance of compound in octanol phase after shaking with PBS

8. Cell viability studies

Solubility

The studied compounds showed excellent solubility in pure DMSO and low solubility in culture media. All biological experiments were thus conducted using 5 mM stock solutions in DMSO that were diluted with culture medium to produce a final concentration of 0.5% DMSO for all experiments unless specified otherwise.

Cell viability experiments

HeLa cells were harvested, pelleted by centrifugation, and resuspended in cell culture medium. The MTT assays were carried out in 96 well plates at a starting density of 1500 cells per well in 100 µL of culture medium. The next day, a dilution series of 7 different concentrations was prepared for each evaluated compound. After 24 h of incubation, the cells were treated with 100 µL of culture medium containing the prepared concentrations of the respective compounds to reach a final volume of 200 µL in each well. As a negative control, one series of cells was treated with the vehicle only (0.5% DMSO). The cells were then incubated with the compound for 72 hours, followed by an addition of 50 µL PBS containing MTT dye (2.5 mg/mL). After a further incubation of 2 h, the medium was aspired off, and 200 µL DMSO was added to solubilize the precipitated formazan dye. The formazan absorption was measured on a BMG LABTECH CLARIOstar Plus plate reader at 550 nm, using a reference wavelength of 620 nm. Using the mean absorbance of each set of identically treated wells, a dose-response curve was produced and normalized to the wells containing untreated cells to allow for plate-to-plate comparison. The dose-response curves were then subjected to non-linear regression analysis performed in Microsoft Excel to determine IC_{50} values. Data is shown as the mean of three replicate experiments, and error bars represent the standard deviation.

Proliferation profiles

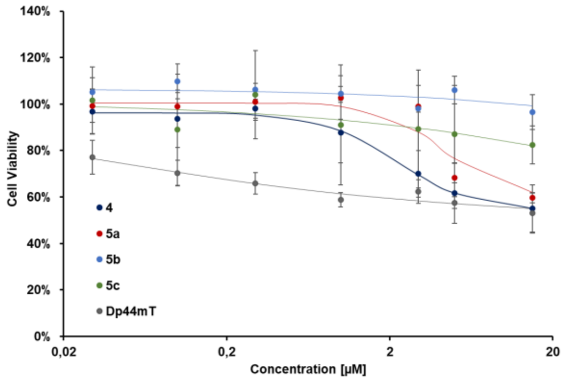

Figure S48: Proliferation profiles HeLa cells after 24 h incubation with compounds **4** and **5a–c**; Dp44mT included as positive control.

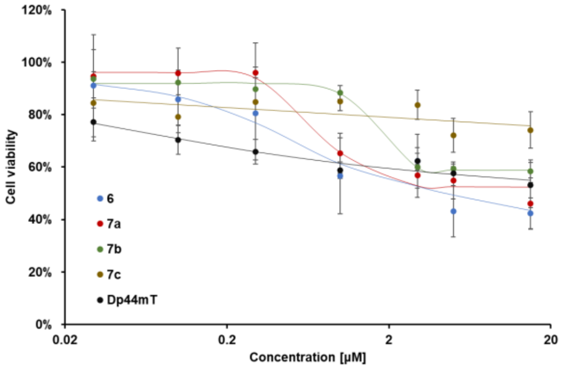

Figure S49: Proliferation profiles HeLa cells after 24 h incubation with compounds **6** and **7a–c**; Dp44mT included as positive control.

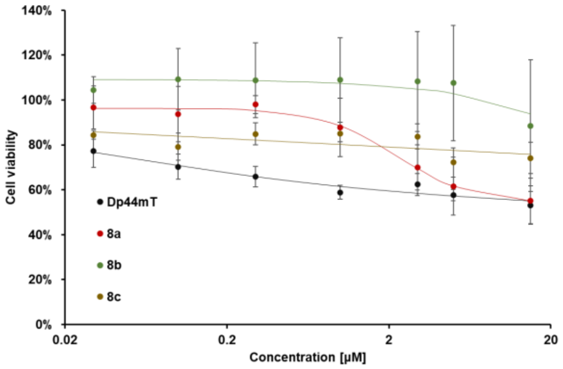

Figure S50: Proliferation profiles HeLa cells after 24 h incubation with compounds **8a–c**; Dp44mT included as positive control.

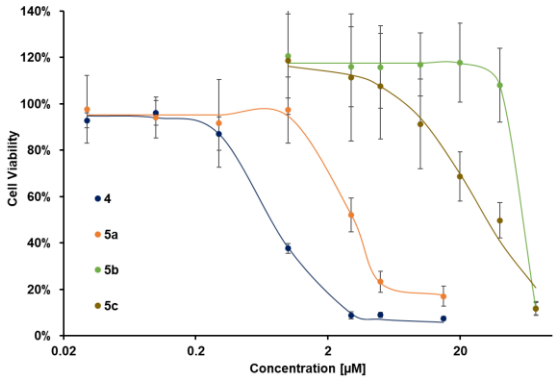

Figure S51: Proliferation profiles HeLa cells after 72 h incubation with compounds **4** and **5a–c**.

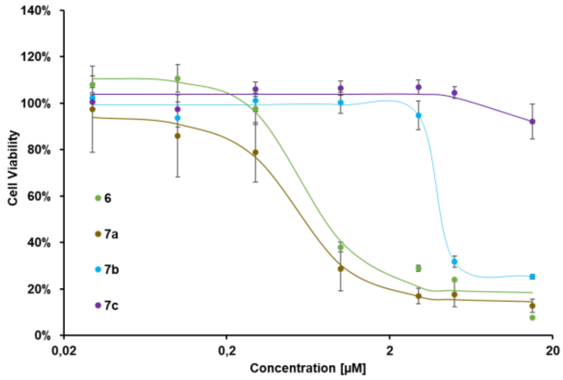

Figure S52: Proliferation profiles HeLa cells after 72 h incubation with compounds **6** and **7a–c.**

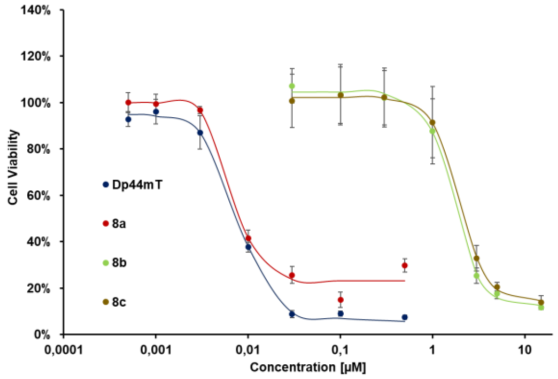

Figure S53: Proliferation profiles HeLa cells after 72 h incubation with **Dp44mT** and compounds **8a–c**. **Dp44mT** used as control for all 72h MTT assays as well.

9. Uptake assay

HeLa cells were harvested and seeded in 100 mm × 21 mm cell culture dishes at a density of 200,000 cells per dish in 10 mL of growth medium. After 96 hours of incubation, when the cells had reached 90% confluency, the growth medium was aspirated and replaced with fresh medium containing 10 µM of a compound belonging to sets **5** or **7**, respectively. The cells were incubated with the compound for 24 h, after which the medium was aspirated, and the cells were washed three times with 10 mL of PBS buffer. Subsequently, the washed cells were

trypsinized for 3 min, and the trypsin reaction was halted by the addition of 3 mL growth media. Cells were suspended in the mixture, and the total cell count was determined using a Bio-Rad TC20 cell counter. The cell suspension was centrifuged at 5000 rpm for 5 minutes, and the supernatant was removed. The cells were then digested using 2 mL of aqua regia (3:1 v/v, 37% HCl and 70% HNO_3, trace metal basis quality (Aldrich, purity ≥99.999%)) and sonicated at 60 °C for 16 h. The dissolved samples were then diluted with 13 mL of HPLC-grade water to reach a final volume of 15 mL. The total metal content of the samples was then determined via ICP-MS analysis (Thermo Scientific iCAP RQ, ASX-560) in Ar plasma in KED mode with He as collision gas. Reference to standards was made, and calibration curves were developed using commercial calibration standards (TraceCert®, Merck).

Uptake assay		
**Fluorophore**	**Compound**	**Metal content (pg/cell)**
**Coumarin**	**5a** (Au(III))	0.032 ± 0.016
	**5b** (Pt(II))	0.016 ± 0.005
	**5c** (Pd(II))	0.007 ± 0.002
**BODIPY**	**7a** (Au(III))	0.035 ± 0.007
	**7b** (Pt(II))	0.013 ± 0.002
	**7c** (Pd(II))	0.033 ± 0.004

Table S3: Whole cell accumulation of compound sets **5** and **7** analyzed using ICP-MS and expressed in terms of total metal content.

10. Fluorescence spectroscopy

5 mM stocks of the respective compounds in DMSO were diluted to produce a final concentration of 10 µM in water.

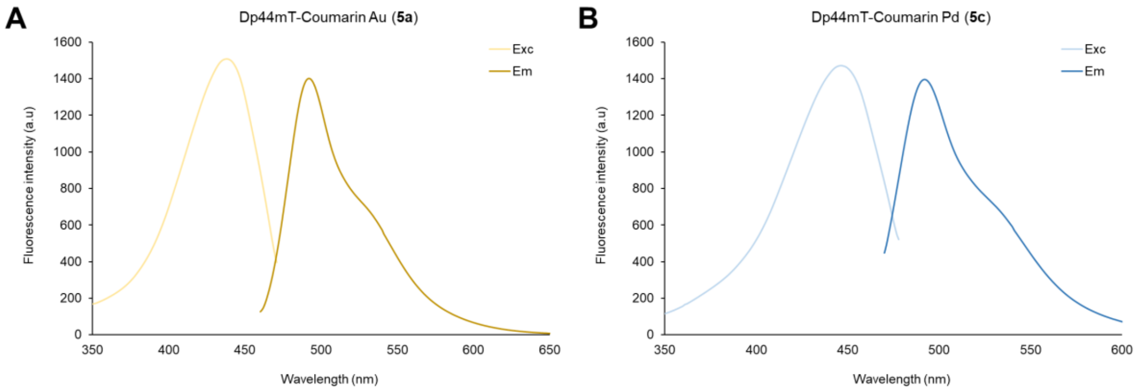

Figure S54: Excitation (Exc) and emission (Em) spectra of compounds (A) **5a** and (B) **5c** at 10 µM in water. For both compounds, λ_{ex} = 450 nm and λ_{em} = 492 nm; Slitwidth: 5 mm; Sensitivity: medium.

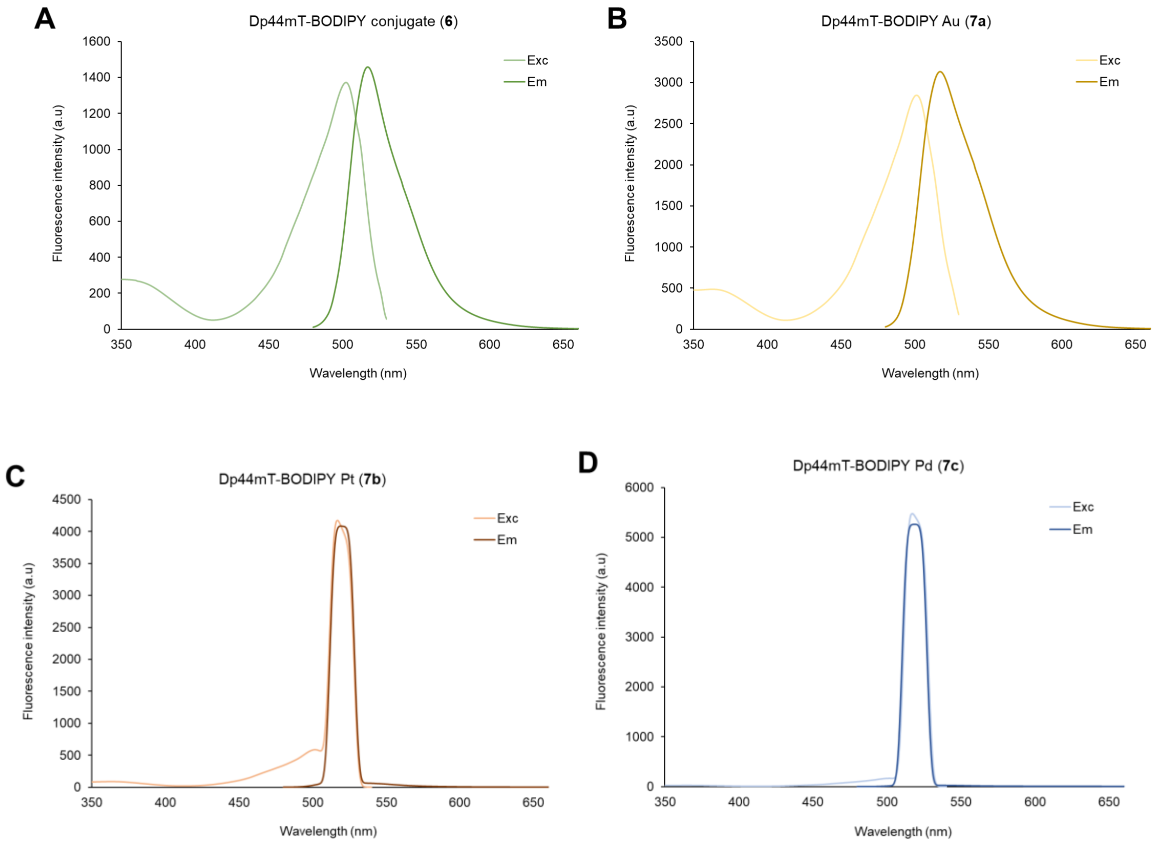

Figure S55: Excitation (Exc) and emission (Em) spectra of BODIPY conjugates **6** and **7** at 10 µM in water. For (A) **6** and (B) **7a** compounds, λ_{ex} = 502 nm and λ_{em} = 519 nm; Slitwidth: 5 mm; Sensitivity: low and medium, respectively. For (C) **7b** and (D) **7c**, λ_{ex} = 519 nm and λ_{em} = 519 nm; Slitwidth: 5 mm; Sensitivity: medium

11. Confocal microscopy

Live-cell imaging was carried out in 8-well cell culture chamber slides from ibidi at a starting density of 20000 cells per well in 200 µL of culture medium. The cells were incubated for 24 h at 37 °C under a 10% CO_2 atmosphere. Then, 100 µL media containing the respective compounds **4**, **5**, **6**, or **7** was added to reach a final volume of 300 µL per well with a final compound concentration of 10 µM. As controls, untreated HeLa cells were prepared under the same conditions but without the active compounds. After 6 h or 24 h of incubation, as appropriate, the media was removed, and the cells were washed twice with DMEM medium without phenol red (clear medium). A clear media solution of 100 nM LysoTracker™ Deep-Red (Invitrogen™) ($\lambda_{exc}/\lambda_{em}$ = 650/660 nm) or 200 nM MitoTracker™ Deep-Red FM (Invitrogen™) ($\lambda_{exc}/\lambda_{em}$ = 644/665 nm) or 750 nM ER-Tracker™ Red (Invitrogen™) ($\lambda_{exc}/\lambda_{em}$ = 587/615 nm) was then appropriately added to the cells, and they were incubated for another 30 min at 37 °C. Afterwards, the cells were washed twice with fresh clear medium and then covered with 200 µL clear medium for the duration of the microscopic investigations. The imaging was carried out at 37 °C using a Leica TCS-SP8 confocal laser scanning microscope equipped with an unpulsed 442 nm laser and a pulsed white light laser (WLL) (470 nm – 670 nm) using hybrid PMT or SMD detectors. Compounds **4** and **5** were excited using the 442 nm laser, and the emission was collected between 470 and 490 nm. Compounds **6** and **7** were excited at 502 nm using the WLL, and the emission was collected between 515 and 530 nm. The tracker dyes were excited at the indicated wavelengths using the WLL, and the emissions were

collected at the appropriate emission wavelengths. Images were acquired at 1024 x 1024 pixels and processed using LASX office program. The corresponding Pearson's Correlation Coefficients (PCCs) were calculated using Fiji (version 1.54f).[7] All experiments were repeated twice to prove reproducibility.

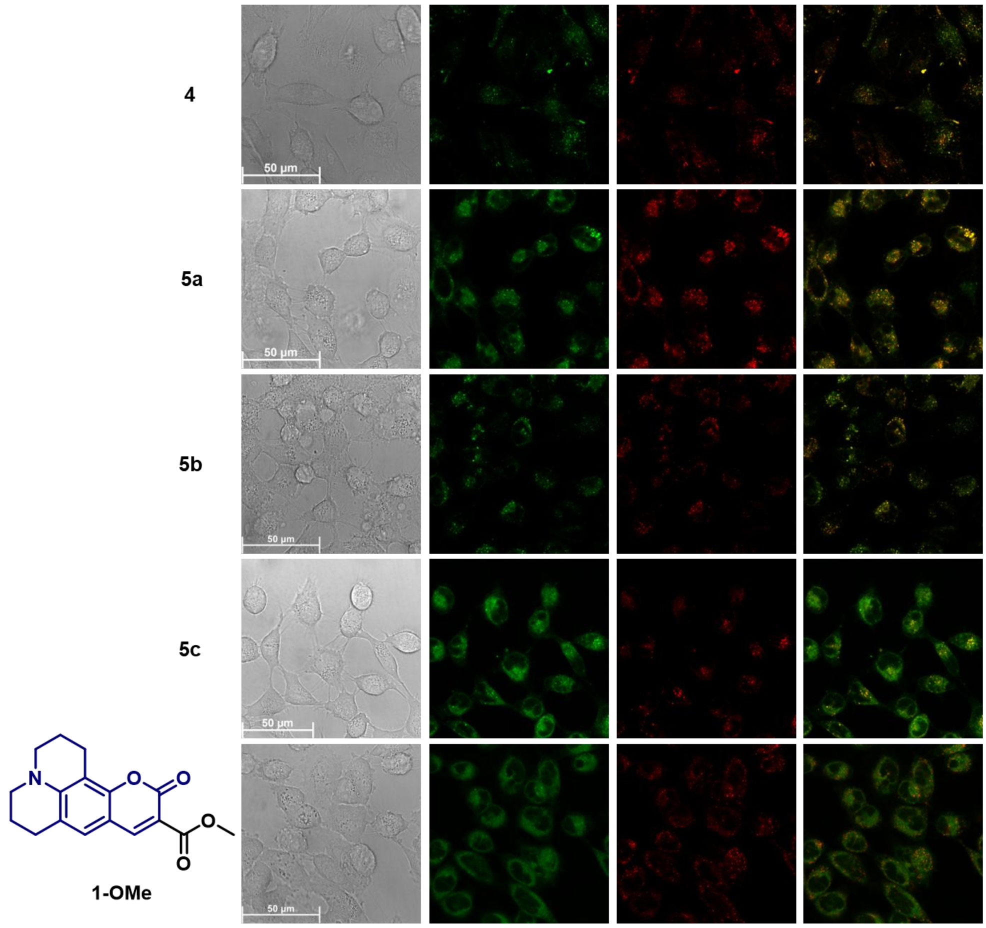

Figure S56: Live-cell images acquired after incubating compounds **4**–**5** and the control **1-OMe** with HeLa cells for 6 h, respectively, at 10 µM. The green channel shows the respective compound and the red channel LysoTracker deep red. The merged channels and the corresponding Pearson Correlation Coefficients (PCC) ± standard deviation are shown on the right of each column.

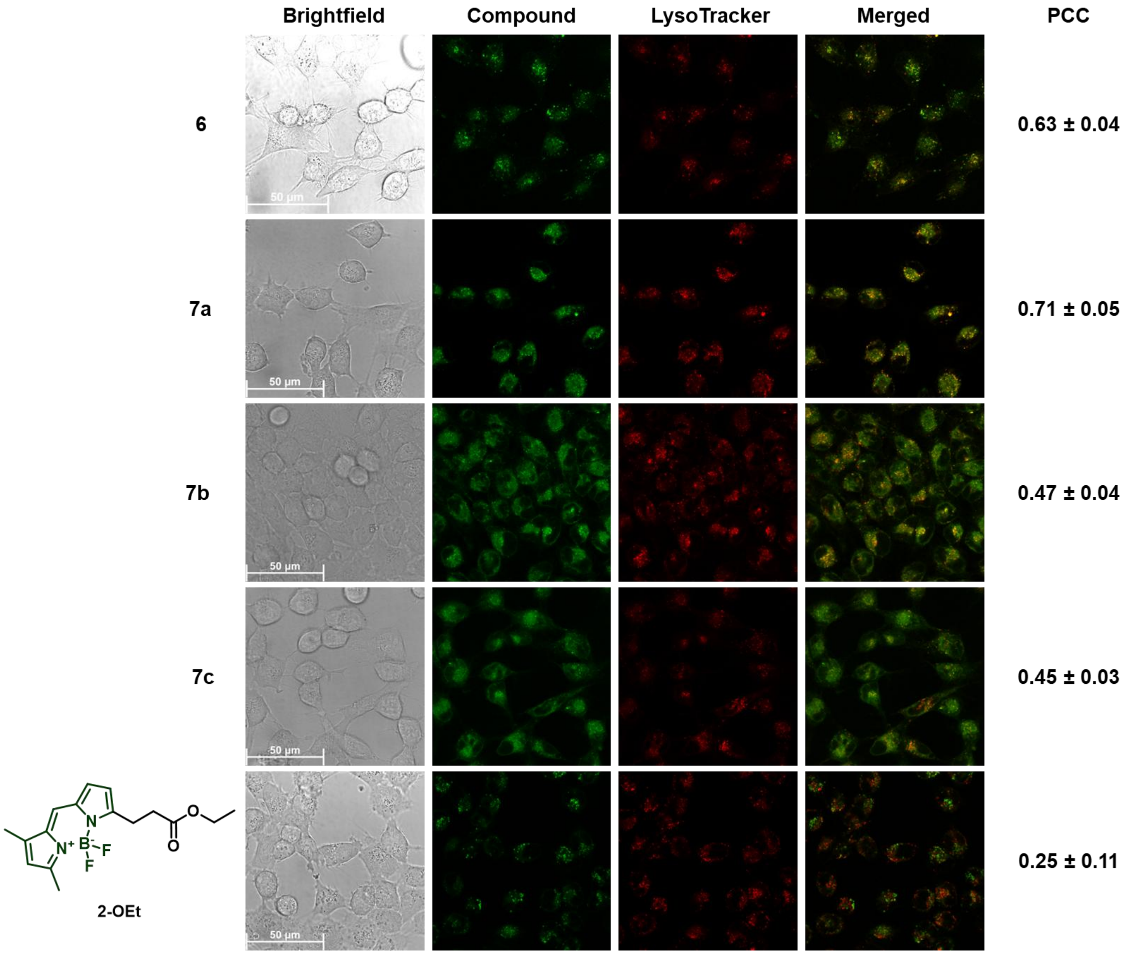

Figure S57: Live-cell images acquired after incubating compounds **6**–**7** and the control **2-OEt** with HeLa cells for 24 h, respectively, at 10 µM. The green channel shows the respective compound and the red channel LysoTracker deep red. The merged channels and the corresponding Pearson Correlation Coefficients (PCC) ± standard deviation are shown on the right of each column.

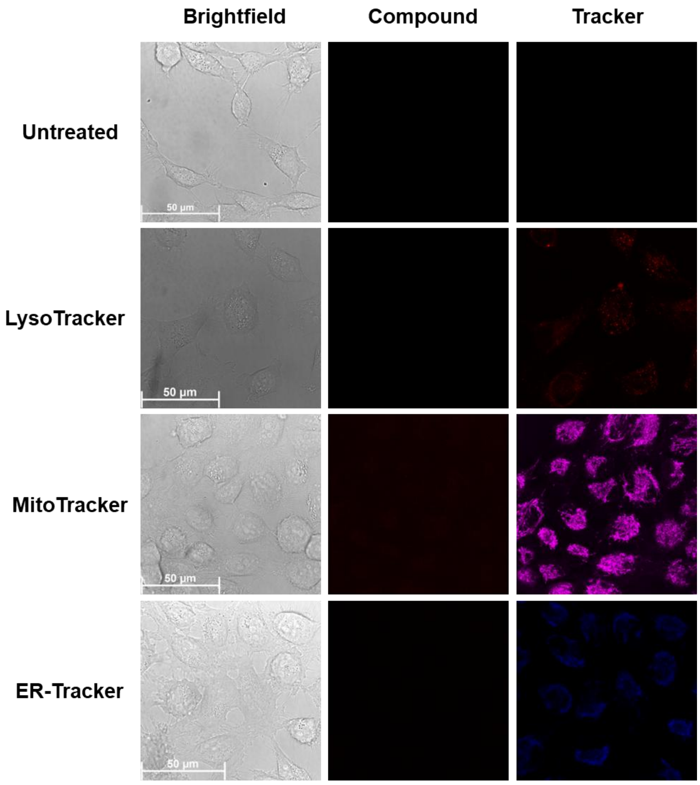

Figure S58: Control images from live-cell confocal microscopy. This includes HeLa cells treated with DMSO-containing media as the negative control, as well as those treated with just the respective organelle trackers.

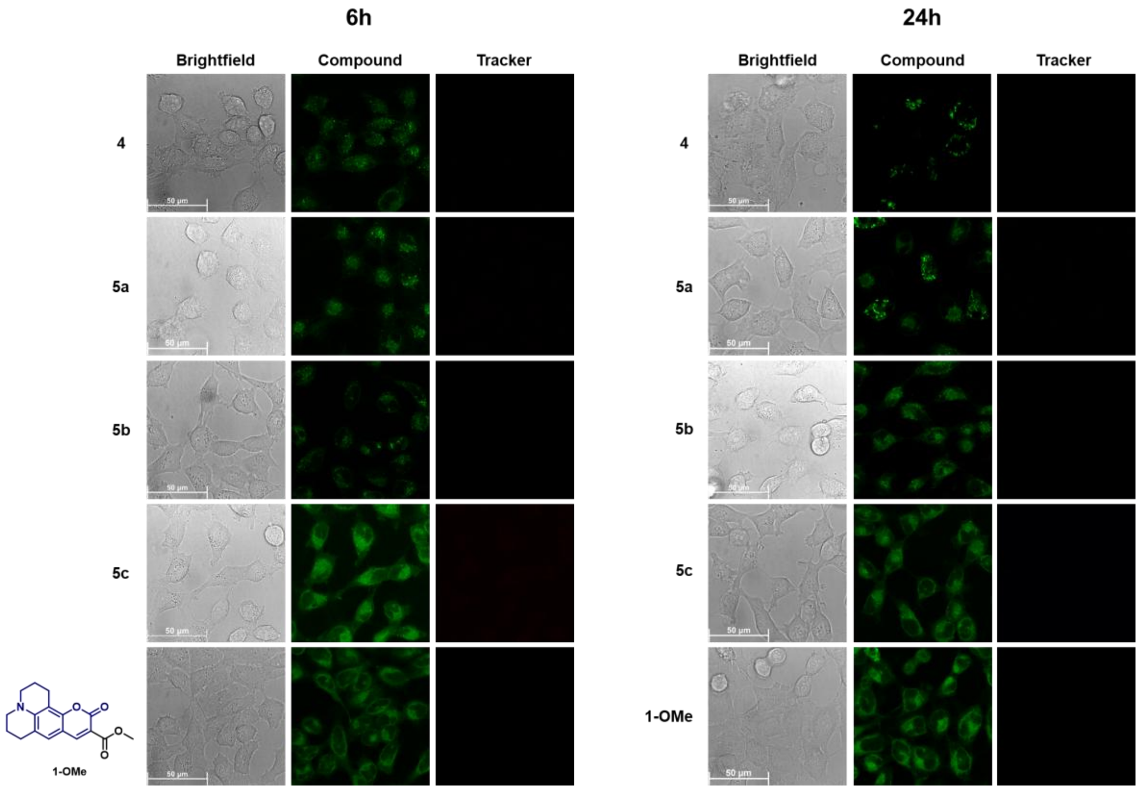

Figure S59: Control images from confocal microscopy of HeLa cells treated with the coumarin conjugates **4** and **5**. The cells were prepared according to the procedure described above while skipping tracker coincubation and images were similarly acquired.

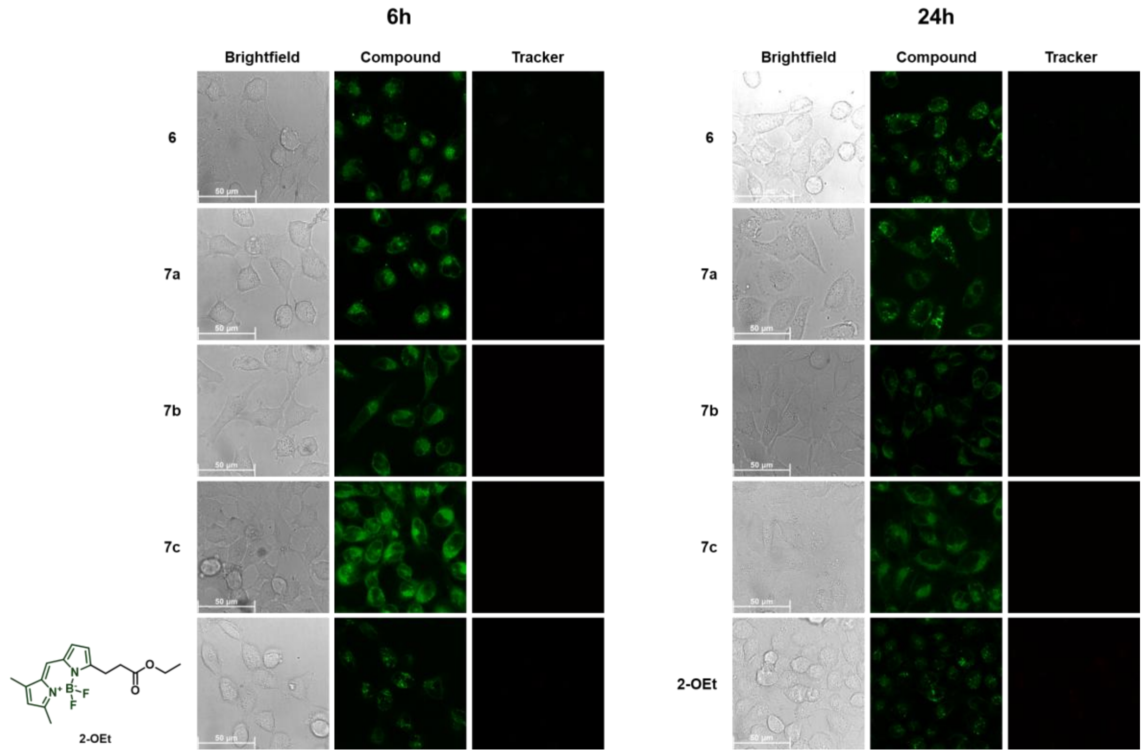

Figure S60: Control images from confocal microscopy of HeLa cells treated with the BODIPY conjugates **6** and **7**. The cells were prepared according to the procedure described above while skipping tracker coincubation and images were similarly acquired.

12. X-ray fluorescence imaging (HeLa)

HeLa cells were prepared in Ibidi 8-well plates, with each well containing a sterilized SiN membrane (Silson Ltd) onto which cells were allowed to adhere. LysoTracker^{TM} Deep Red (Invitrogen^{TM}) was used as organelle stain. In the final step, the cells were washed twice and then covered with PBS buffer solution for light microscopy imaging.

The imaging and cryofixation were accomplished following a previously published procedure,[8,9] which will be briefly described here: First, the cells on the SiN membranes were imaged with a Nikon Eclipse Ti fluorescence microscope. After imaging, the SiN membranes were blotted using cellulose paper and the samples were immersed quickly into a liquid ethane/propane mixture at liquid nitrogen temperature. After cryofixation, the samples were stored in liquid nitrogen until the X-ray fluorescence measurements.

The XRF measurements and data analysis have been previously described[10,11]; in brief, XRF measurements were carried out at the Hard X-ray Micro/Nano-Probe Beamline P06, DESY (Hamburg).[12] A custom cryo-vacuum chamber was used for the measurement, and the samples were measured at a base pressure of $\approx 10^{-7}$ mbar at a sample temperature of 120 K. The synchrotron beam (17 keV) was focused to a size of 580 nm x 560 nm with a flux of 2×10^{10} photons/s. The fluorescence signal was detected by a Rococo 2 detector (PN detectors, München, Germany). The detector was positioned in a backscatter geometry, 6 mm upstream of the sample. The spectra of the four active elements of the Rococo 2 detector were summed up and normalized to the intensity of the excitation beam. All spectra were analyzed by peak deconvolution and fitting using the fast XRF stacking function of the software PyMCA (European Synchrotron Radiation Facility, ESRF).[13] Spatial mass densities were

calculated using a thin film XRF reference sample "RF17-200-S4218-41 "(AXO Dresden, Germany).

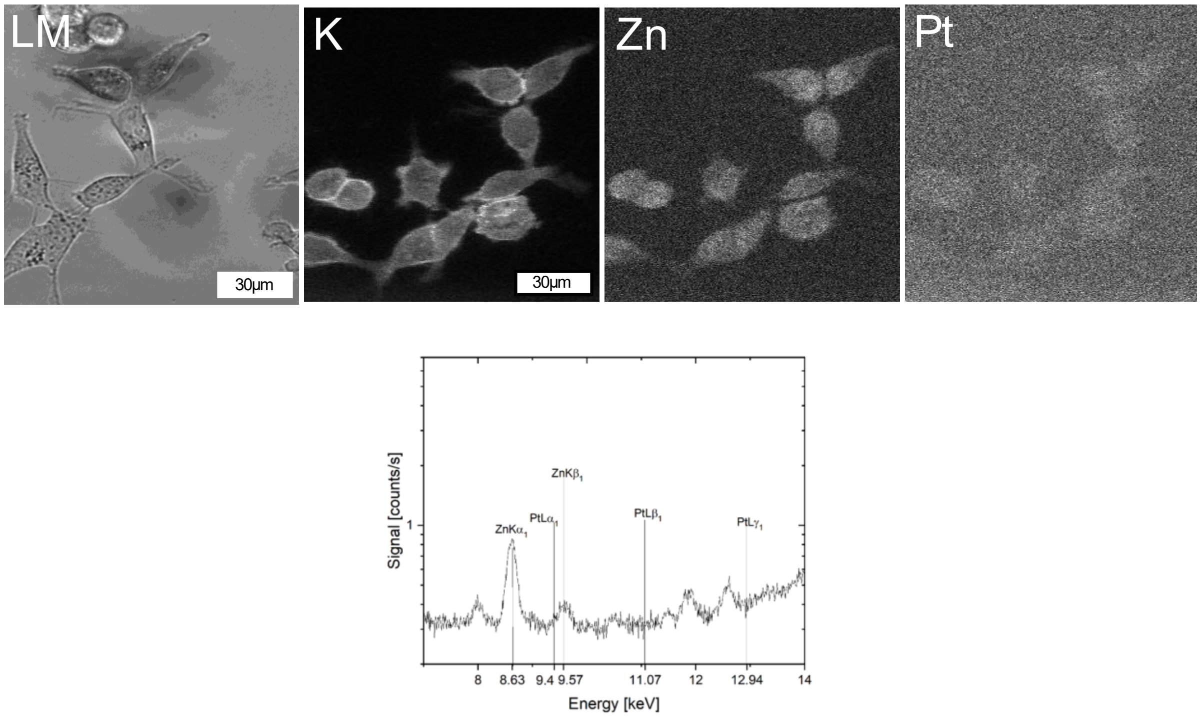

Figure S61: XRF imaging of untreated HeLa cells. From left to right: brightfield/light microscope (LM), XRF images showing the elemental distribution of potassium (K – Kα_1), zinc (Zn– Kα_1), and platinum (Pt – Lβ_1), respectively. Also shown at the bottom is the X-ray fluorescence spectrum, which confirms that the sample is devoid of Pt.

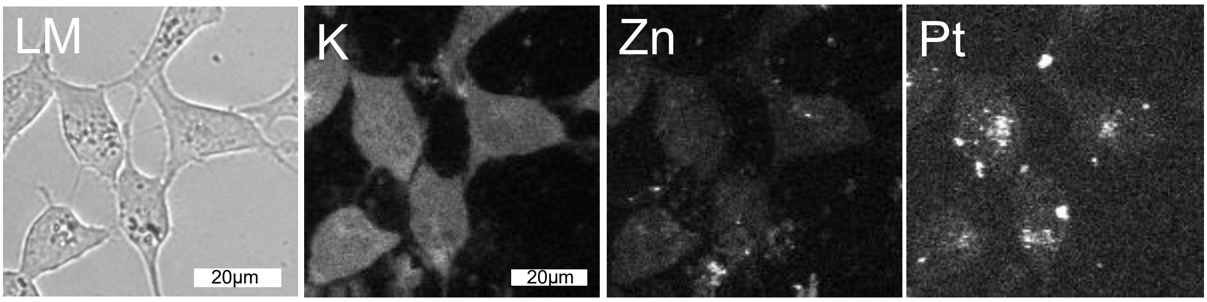

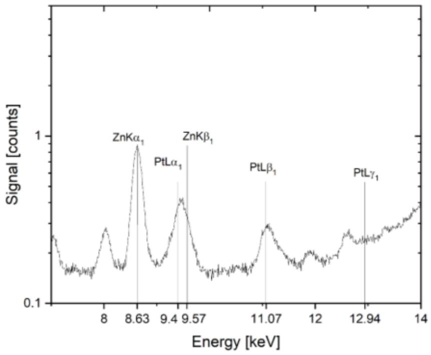

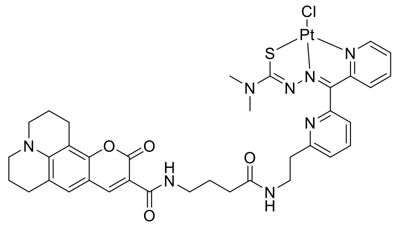

5b

Figure S62: XRF imaging of HeLa cells incubated with 30 µM of **5b**. From left to right: brightfield/light microscope (LM) XRF images showing the elemental distribution of potassium (K – Kα_1), zinc (Zn– Kα_1), and platinum (Pt – Lβ_1), respectively. Also shown at the bottom is the X-ray fluorescence spectrum, which confirms the presence of Pt in the sample.

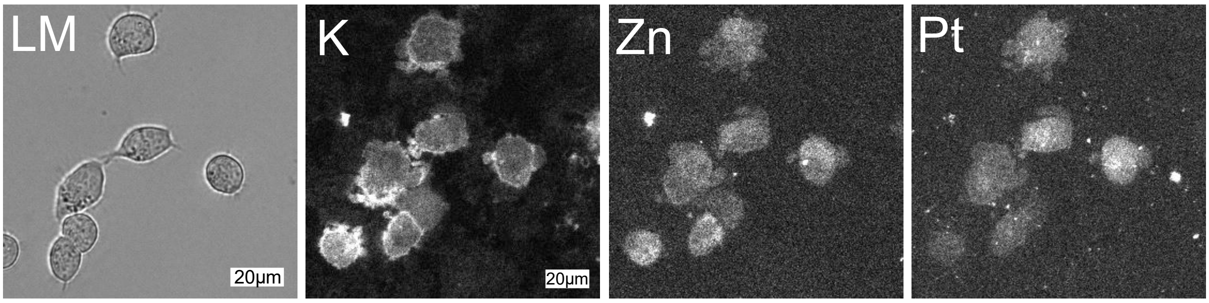

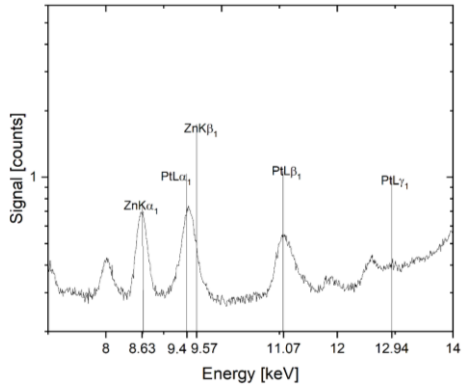

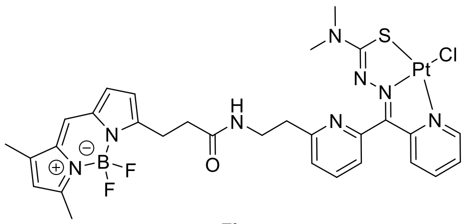

7b

Figure S63: XRF imaging of HeLa cells incubated with 30 μM of **7b**. From left to right: brightfield/light microscope (LM) XRF images showing the elemental distribution of potassium (K – Kα_1), zinc (Zn– Kα_1), and platinum (Pt – Lβ_1), respectively. Also shown at the bottom is the X-ray fluorescence spectrum, which confirms the presence of Pt in the sample.

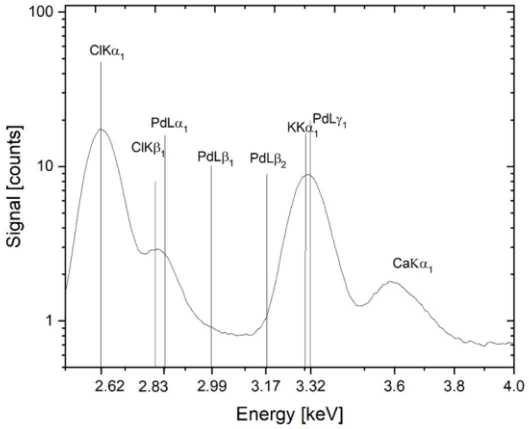

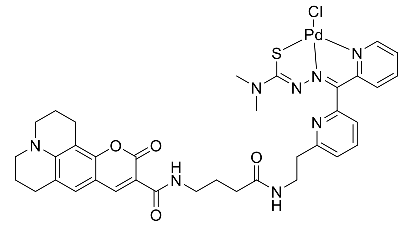

5c

Figure S64: X-ray fluorescence spectrum of **5c** which shows the overlap between the palladium (Pd – Lα_1) and chlorine (Cl – Kβ_1) X-ray emission energies.

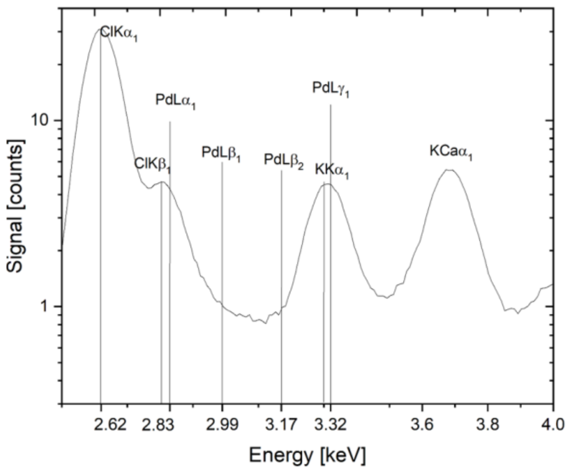

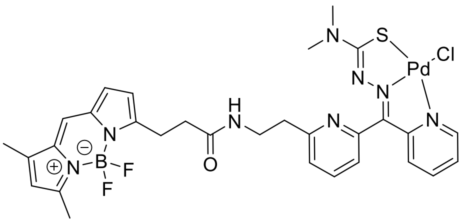

7c

Figure S65: X-ray fluorescence spectrum of **7c** which shows the overlap between the palladium (Pd – Lα_1) and chlorine (Cl – Kβ_1) X-ray emission energies.

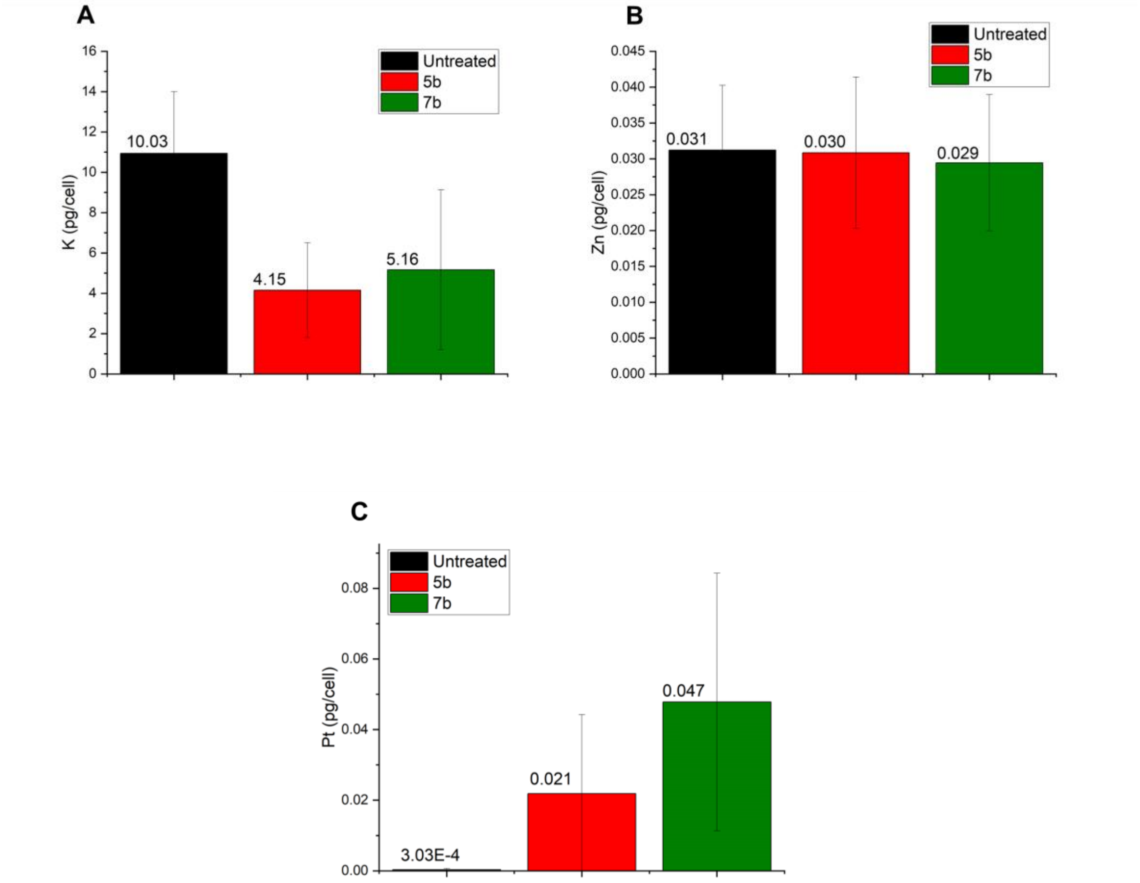

Figure S66: Average intracellular concentrations (in pg/cell) of (A) potassium (K – Kα_1), (B) Zn (Zn– Kα_1), and (C) platinum (Pt – Lβ_1) determined from cell area and area concentration measurements for untreated, **5b**-treated, and **7b**-treated HeLa cells, respectively.

13. References

[1] G. M. Sheldrick, ""SHELXT – Integrated space-group and crystal-structure determination,"" *Acta Crystallogr. Sect. Found. Adv.* **2015**, *71*, 3–8.

[2] G. M. Sheldrick, ""Crystal structure refinement with SHELXL,"" *Acta Crystallogr. Sect. C Struct. Chem.* **2015**, *71*, 3–8.

[3] O. V. Dolomanov, L. J. Bourhis, R. J. Gildea, J. a. K. Howard, H. Puschmann, ""OLEX2: a complete structure solution, refinement and analysis program,"" *J. Appl. Crystallogr.* **2009**, *42*, 339–341.

[4] N. Sheernaly, A. Steinbrueck, J. Ochs, F. Peeters, R. Fiedler, F. Narberhaus, J. Heinen-Weiler, N. Metzler-Nolte, ""Design and application of a non-toxic platinum derivative of the metal chelator Dp44mT for use as a long-term stable lysosome tracker,"" *Chem. Commun.* **2025**, *61*, 9964–9967.

[5] S. Krajcovicova, J. Stankova, P. Dzubak, M. Hajduch, M. Soural, M. Urban, ""A Synthetic Approach for the Rapid Preparation of BODIPY Conjugates and their use in Imaging of Cellular Drug Uptake and Distribution,"" *Chem. – Eur. J.* **2018**, *24*, 4957–4966.

[6] N. Sheernaly, I. Shajan, A. Steinbrueck, B. Albada, N. Metzler-Nolte, ""Synthesis and biological evaluation of thiosemicarbazone-based antibody–drug conjugates,"" *RSC Med. Chem.* **2025**, *16*, 3512–3521.

[7] J. Schindelin, I. Arganda-Carreras, E. Frise, V. Kaynig, M. Longair, T. Pietzsch, S. Preibisch, C. Rueden, S. Saalfeld, B. Schmid, J.-Y. Tinevez, D. J. White, V. Hartenstein, K. Eliceiri, P. Tomancak, A. Cardona, ""Fiji: an open-source platform for biological-image analysis,"" *Nat. Methods* **2012**, *9*, 676–682.

[8] C. Rumancev, A. Gräfenstein, T. Vöpel, S. Stuhr, A. R. von Gundlach, T. Senkbeil, J. Garrevoet, L. Jolmes, B. König, G. Falkenberg, S. Ebbinghaus, W. H. Schroeder, A. Rosenhahn, ""X-ray fluorescence analysis of metal distributions in cryogenic biological samples using large-acceptance-angle SDD detection and continuous scanning at the Hard X-ray Micro/Nano-Probe beamline P06 at PETRA III,"" *J. Synchrotron Radiat.* **2020**, *27*, 60–66.

[9] C. Rumancev, T. Vöpel, S. Stuhr, A. von Gundlach, T. Senkbeil, S. Ebbinghaus, J. Garrevoet, G. Falkenberg, B. De Samber, L. Vincze, A. Rosenhahn, W. Schroeder, ""Micro x-ray fluorescence analysis of trace element distribution in frozen hydrated HeLa cells at the P06 beamline at Petra III,"" *Biointerphases* **2021**, *16*, 011004.

[10] A. Gräfenstein, D. Brückner, C. Rumancev, J. Garrevoet, V. Galbierz, W. H. Schroeder, C. G. Schroer, G. Falkenberg, A. Rosenhahn, ""Single-Slice XRF Mapping of Light Elements in Frozen-Hydrated Allium schoenoprasum via a Self-Absorption-Corrected Hyperspectral Tomographic Reconstruction Approach,"" *Anal. Chem.* **2023**, *95*, 10186–10195.

[11] A. Gräfenstein, C. Rumancev, R. Pollak, B. Hämisch, V. Galbierz, W. H. Schroeder, J. Garrevoet, G. Falkenberg, T. Vöpel, K. Huber, S. Ebbinghaus, A. Rosenhahn, ""Spatial Distribution of Intracellular Ion Concentrations in Aggregate-Forming HeLa Cells Analyzed by μ-XRF Imaging,"" *ChemistryOpen* **2022**, *11*, e202200024.

[12] C. G. Schroer, P. Boye, J. M. Feldkamp, J. Patommel, D. Samberg, A. Schropp, A. Schwab, S. Stephan, G. Falkenberg, G. Wellenreuther, N. Reimers, ""Hard X-ray nanoprobe at beamline P06 at PETRA III,"" *Nucl. Instrum. Methods Phys. Res. Sect. Accel. Spectrometers Detect. Assoc. Equip.* **2010**, *616*, 93–97.

[13] V. A. Solé, E. Papillon, M. Cotte, Ph. Walter, J. Susini, ""A multiplatform code for the analysis of energy-dispersive X-ray fluorescence spectra,"" *Spectrochim. Acta Part B At. Spectrosc.* **2007**, *62*, 63–68.
